# Disruptive behavior and emotional problems in children screened in routine health care: prevalence and effectiveness of indicated prevention

**DOI:** 10.1186/s13034-025-00949-7

**Published:** 2025-08-14

**Authors:** Cornelia Beate Siegmund, Julia Zink, Patricia Theresa Porst, Max Weniger, Susanne Knappe, Maria McDonald, Veit Roessner, Katja Beesdo-Baum

**Affiliations:** 1 Behavioral Epidemiology, Institute of Clinical Psychology and Psychotherapy, TUD Dresden University of Technology, Chemnitzer Straße 46, 01187 Dresden, Germany; 2 Evangelische Hochschule Dresden (ehs), University of Applied Sciences for Social Work, Education and Nursing, Dresden, Germany; 3 Department of Child and Adolescent Psychiatry, Faculty of Medicine, TUD Dresden University of Technology, German Center for Child and Adolescent Health (DZKJ), partner site, Dresden, Germany

**Keywords:** Children, Disruptive behavior, Emotional problems, Indicated prevention, Quality of life

## Abstract

**Background:**

Disruptive behavior and emotional problems are common in children and often reduce quality of life. This study aimed to screen for these problems and to examine the effectiveness of child-based indicated prevention.

**Methods:**

*N* = 3231 children`s disruptive behavior and emotional problems were screened using the Strengths and Difficulties Questionnaire (SDQ) during routine pediatric health check-ups for usually 5- to 10-year old’s. We examined the prevalences of disruptive behavior and emotional problems (*n* = 2825) and its association with quality of life (KINDL; *n* = 1104). If indicated, children were recommended to participate in the prevention program “Baghira training” (nine 90 min group sessions and one parents’ evening) or “Tiger training” (two one-on-one and nine group sessions of 60 min each). To evaluate the training effectiveness of the two indicated prevention programs, SDQ and KINDL scores were followed-up for 6 and 12 months post screening and compared between the Training group (SDQ *n* = 337; KINDL *n* = 334; additionally divided into Baghira and Tiger), children not participating despite indication (NoTraining; SDQ *n* = 595; KINDL *n* = 146; additionally divided into NoBaghira and NoTiger), healthy children (SDQ *n* = 1928; KINDL *n* = 907), and children with clinical symptom levels (SDQ *n* = 85; KINDL *n* = 54) using mixed effect models.

**Results:**

37.0% of the children exhibited disruptive behavior or emotional problems, which were associated with impaired quality of life. The Training group perceived greater symptom reduction in emotional problems than NoTraining, and quality of life increases compared to decreases in NoTraining. The Tiger group showed improvement in symptomatology and quality of life compared to deterioration in NoTiger. The Baghira group also improved, though improvement was similar to NoBaghira apart from symptom reduction in emotional problems in Baghira compared to a symptom increase in NoBaghira. Effects sizes were predominantly small to medium.

**Conclusions:**

Disruptive behavior and emotional problems in children are frequent and impair quality of life. Indicated prevention may improve symptomatology and quality of life. Specifically, the Tiger training is verifiably effective; for the Baghira training, effectiveness is implicated but needs further empirical evaluations.

**Supplementary Information:**

The online version contains supplementary material available at 10.1186/s13034-025-00949-7.

## Introduction

Disruptive behavior and emotional problems are common mental health problems during childhood [22, 26]. Hölling et al. [22] for example demonstrated that salient disruptive behavior and emotional problems are present in 6.6% respectively 15.8% of the 3–6 year olds, and in 10.4% respectively 15.1% of the 7–10 year olds. This is of significance as subclinical problems increase the risk for further mental health problems and progression into full-blown mental disorders [8, 15]. Furthermore, childhood mental disorders increase the risk for mental disorders in adulthood [13, 25] and are associated with a negative impact on the further development, physical health, interpersonal relations, and professional life and cause high costs in health and social services [3, 12, 27]. Additionally, mental health problems are associated with an impaired quality of life [42]. Moreover, many disruptive behavior (e.g. oppositional defiant disorder, conduct disorder, attention deficit hyperactivity disorder) and emotional disorders (e.g. anxiety) firstly manifest during childhood or early adolescence [24, 28]. Thus, prevention measures for disruptive behavior and emotional problems as early as childhood are crucial to promote mental health, to counteract an adverse development over the lifespan and to reduce the high societal costs of mental disorders. Research demonstrates that different prevention programs aiming at disruptive behavior and emotional problems are cost effective [27, 29, 30] and are able to reduce symptom severity and even the incidence of new onset cases [14, 32, 43]. However, not all programs appear to be equally effective, as there is evidence that indicated prevention which targets individuals with emerging problems is more effective than universal prevention measures aimed at the whole community because the latter includes participants without need of prevention who continue a healthy development with or without intervention [6, 7]. Furthermore, effectiveness differs between programs [16, 32, 41, 46]. For example, Neil and Christensen [32] reviewed school-based anxiety prevention programs and of the eight identified studies only four showed greater post-intervention reductions in anxiety symptoms compared with the control group, albeit, of the six trials that examined follow-up differences, five found significant positive effects. Smedler et al. [43] conducted a review on prevention programs aiming at disruptive behavior assessed in randomized controlled trials and found medium effects for reduced disruptive behavior symptoms up to 12 months for the family support program Family Check-Up and the school- and parenting program Coping Power. However, other indicated programs and long-term effects exhibited inconsistent results [43]. Unfortunately, in contrast to such school- and parent-based programs, research on the effectiveness of different child-based indicated prevention programs is insufficient. Although for emotional problems, there is some evidence on the effectiveness of indicated child-based prevention programs [46, 48], regarding disruptive behavior problems such literature is lacking. Furthermore, to our knowledge there is no study examining the effectiveness of indicated prevention programs on participants’ quality of life. Hence, this topic needs further evaluation and may shed light on well-being and daily functioning as relevant outcomes for the evaluation of preventive interventions, apart from symptom change.

Based on the mentioned fact that indicated prevention programs show greater effects than universal programs, it seems useful to implement screenings and indicated prevention programs in general health care in order to identify and refer potentially affected children from the general population. Thus, we initiated the PROMPt project conducted in the area of Dresden, Germany, to implement an innovative care chain where routine pediatric health check-ups were supplemented with a brief screening questionnaire to identify children with emerging disruptive behavior and emotional problems in order to allow the pediatrician to recommend an indicated prevention program (for detailed information see methods section and Weniger et al., [50]. Following this innovative care chain, the current paper aims 1) to report the prevalence of disruptive behavior and emotional problems among children in the general population and associations to quality of life, and 2) to examine the effectiveness of indicated child-based group prevention programs for disruptive behavior and emotional problems. We hypothesize reduced quality of life in children with disruptive behavior and/or emotional problems compared to unaffected children and improvement of symptomatology and quality of life in children participating in an indicated prevention program. For children who have been screened at normal or abnormal symptom levels and children not participating in a prevention program despite indication, we do not expect any changes in their symptomatology and quality of life scores at the respective goup level. That is because children with mental health problems show both stable as well as waxing and waning patterns of symptoms [5].

## Methods

### Study procedure

#### Screening and recruitment

The present study uses data from the PROMPt project, a prospective screening and indicated prevention implementation study [50]. It was conducted in the municipal area of Dresden, Germany, from 10/2018 until 09/2022. Children were screened for disruptive behavior and emotional problems during their regular health check-ups at their pediatrician (U9-U11 for usually 5 to 10 year olds) using the parent report of the Strengths and Difficulties Questionnaire [18, 19]. Based on the SDQ results and the pediatrician’s clinical expertise, parents received feedback and – if indicated – a recommendation for further action from their pediatrician. For children with disruptive behavior problems, the participation in the indicated prevention program adapted from the Baghira group training for children with oppositional and aggressive behavior by Aebi et al. [1] [short: Baghira training] was recommended. The indicated prevention program “Mutig werden mit Til Tiger” (Becoming brave with Til Tiger, short: Tiger training, Ahrens-Eipper et al., [2] was recommended for children screened positively for emotional problems. Children with abnormal (i.e. clinically relevant) levels of mental health problems were advised to contact counseling or therapeutic institutions for further diagnostics and, if necessary, treatment.

#### Process for training participation

Families interested in participating in the recommended indicated prevention program were to contact the study team directly. Families that did not do so within 3–4 weeks despite a prevention recommendation were attempted to be contacted by the study team up to five times to determine their interest in participating, provided that consent to contact has been given. Additionally to the children screened during pediatric health check-ups, other accession routes to the indicated prevention programs were possible and observed in PROMPt, e.g. self-assignment, recommendation by friends or acquaintances if no regular health check-up was coming up soon or if the child had private health insurance.

All interested families were invited to an on-site initial interview with a psychologist (project team member) at the TUD Dresden University of Technology (TUD) to evaluate if the child would benefit from the indicated prevention program and inclusion criteria were fulfilled. Exclusion criteria for training participation were a diagnosed disruptive behavior or emotional ICD-10 mental disorder or unstable medication in the last 6 months, a current psychotherapeutic treatment, and acute endangerment of self or others to ensure that children’s symptomatology was within the prevention spectrum and in line with clinical guidelines. In case of accession through an additional route, families also filled in the SDQ during the interview. Children evaluated as eligible for participating in the Baghira or Tiger training were assigned to a similar aged training group (± 1 year) of three to five children (M = 4.24, SD = 0.50; ideal group size of six was reduced due to the Covid-19 pandemic) by the study team. If children were eligible for participating in both trainings due to co-occurring disruptive behavior and emotional problems, a participative decision was derived between study team member, parents and child, if applicable, as to which training was more appropriate. In six cases, both trainings were conducted consecutively (three each with Baghira or Tiger training first). For training participation, all legal guardians gave their written informed consent and children their verbal consent.

#### Assessments

Questionnaire assessments took place at four time points: screening at the pediatrician’s office or during initial interview by the study team, T0 shortly after screening or before begin of training, T1 approximately 6 months after screening or shortly after end of training, and T2 approximately 12 months after screening (i.e. 6 months after T1 or end of training). At screening, the SDQ and a short project specific questionnaire including socio-demographic data were completed in paper-pencil. For training participants, T0 and T1 were administered via tablet at the TUD and T2 via online questionnaire. Participating families received 10€ for each fully completed assessment. For families not participating in a training, all questionnaires at T0, T1 and T2 were filled in online. These families could win family games in a raffle for each fully completed assessment. All families gave their written informed consent for participation in the questionnaire assessments.

### Measures

#### Strengths and Difficulties Questionnaire (SDQ)

The German version of the SDQ for 4 to 17 year olds [18, 19] was administered as a screening instrument at screening, T1 and T2. It consists of 25 items with 5 items each for the sub-scales emotional problems, conduct problems, hyperactivity/inattention, peer relationship problems and prosocial behavior. The questions referred to the past six months and were rated on a 0- to 2-point response scale (0 = not true, 1 = somewhat true, 2 = certainly true). For the project flow and the current analyses, the subscales conduct problems (to determine disruptive behavior) and emotional problems were considered, because they were the basis for the decision on the prevention indication at screening. When calculating the sub-scales’ sum scores, a maximum of two missing values each were imputed by the child’s mean score of the respective sub-scale (corresponding to Ravens-Sieberer et al., [37]. Higher scores indicated greater problems. For the PROMPt project, the usual cut-offs for categorization [18] into normal, borderline and abnormal scores (conduct problem scale 0–2, 3, 4–10; emotional problem scale 0–3, 4, 5–10) were slightly modified in order to reach more children with potential prevention indication (conduct problem scale 0–2, 3–5, 6–10; emotional problem scale 0–3, 4–6, 7–10). In the analysis sample, the total SDQ showed good internal consistency (α = 0.85 − 0.86 depending on measurement time point). In previous work, the SDQ sub-scales of the parent version were found to have moderate to satisfactory test-retest reliability and concurrent validity [45].

#### KINDL-R

We administered the German parent versions of the KINDL-R Quality of Life Questionnaire for Children, using the Kiddy-KINDL-R version for children not attending school and the Kid-/Kiddo-KINDL-R version for schoolchildren [34, 35]. Each version consists of six sub-scales – physical well-being, emotional well-being, self-esteem, family, friends and everyday functioning – with four items each. Items are rated on a 5-point response scale (never, seldom, sometimes, often, all the time). Both versions are identical besides the sub-scale everyday functioning, which contains questions either about nursery school/kindergarten or school. For analyses we combined both versions (referring to as “KINDL”), because many of the nursery school/kindergarten children at T0 started school until T2 and therefore switched between versions and score calculation was identical. Sum scores were calculated with a maximum of 30% missing values being imputed by the child’s mean score of the respective sub-scale according to the manual. Higher scores indicated greater quality of life. In the analysis sample, the Kiddy-KINDL-R and Kid-/Kiddo-KINDL-R showed good internal consistency (α = 0.86 − 0.89 depending on version and measurement time point). The KINDL has been shown to have moderate to satisfactory test-retest reliability and convergent validity [9, 34, 49].

### Sample

**Fig. 1:**
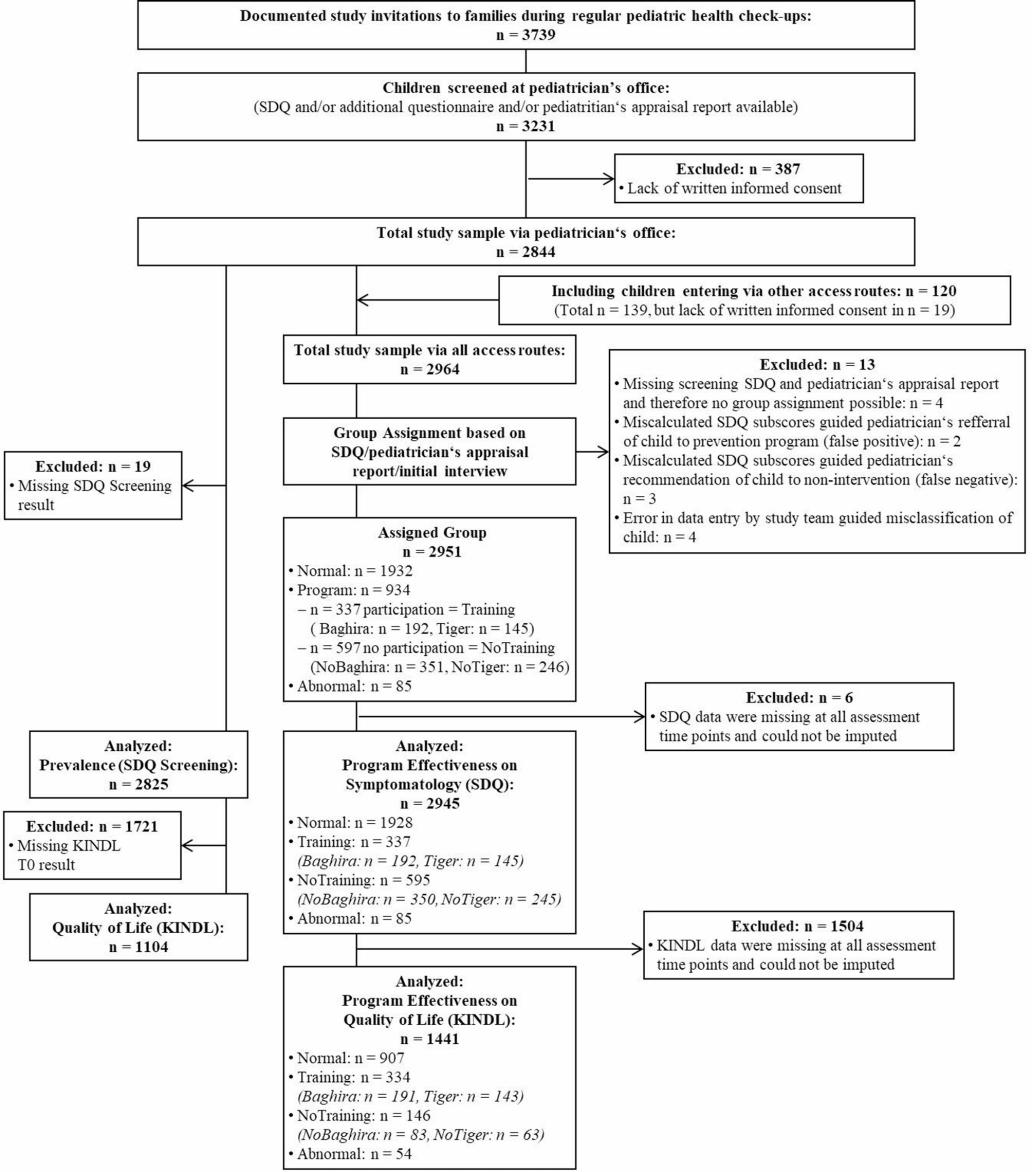
Flowchart of the analysis samples. n = number of participants; SDQ = Strengths and Difficulties Questionnaire; KINDL = Kiddy-KINDL-R and Kid-/Kiddo-KINDL-R Quality of Life Questionnaire for Children; Normal = children evaluated as normal with no recommendation for indicated prevention participation; Training = children who participated in an indicated prevention program after recommendation; NoTraining = children who did not participate in an indicated prevention program despite recommendation; Abnormal = children with abnormal or clinically significant disruptive behavior or emotional problems or that did not fullfill inclusion criteria for participation in an indicated prevention program; Baghira = children who participated in the Baghira training; Tiger = children who participated in the Tiger training; NoBaghira = children who did not participate in the Baghira training despite a recommendation including children with a recommendation for both trainings and a higher or equal SDQ conduct problems than emotional problems score; NoTiger = children who did not participate in the Tiger training despite a recommendation including children with a recommendation for both trainings and a higher SDQ emotional problems than conduct problems score

A flowchart of the analysis samples is illustrated in Fig. 1. Further details on the full study sample of the PROMPt project can be found in Weniger et al. [51]. Briefly, *n* = 3739 study invitations to families during the regular pediatric health check-ups were documented. Of these, *n* = 3231 children were screened at the pediatrician’s office (response rate based on documented study invitations: 86.4%), of which *n* = 387 had to be excluded due to a lack of written informed consent, resulting in a total study sample of *n* = 2844. As the SDQ screening result was missing, another *n* = 19 subjects had to be excluded from the total sample to build the screening sample for analyzing the prevalence (SDQ screening), which finally included *n* = 2816 children. For the analysis of quality of life (KINDL at T0), *n* = 1104 children were assessed, as the KINDL T0 result was missing for a further *n* = 1721 subjects. For the analyses of program effectiveness, a separate sample was composed based on the group assignment following the SDQ screening, pediatricians’ appraisal and initial interview. For this, the total sample was extended by *n* = 120 children entering the project via other access routes. *N* = 13 subjects had to be excluded because group assignment was not possible, resulting in an assigned group sample of *n* = 2951. Of these, *n* = 1932 were evaluated as normal with no recommendation for prevention participation (Normal). *N* = 934 were recommended to participate in one of the two indicated prevention programs offered by the study team, of which *n* = 337 participated (Training): *n* = 192 in the Baghira training (Baghira) and *n* = 145 in the Tiger training (Tiger). Families of *n* = 597 children refused training participation despite recommendation (NoTraining) of which *n* = 330 received a Baghira training recommendation (NoBaghira), *n* = 207 received a Tiger training recommendation (NoTiger) and *n* = 60 received a recommendation for both Baghira and Tiger training. The latter were categorized based on their screening SDQ score as NoTiger (*n* = 39) if they had higher emotional than conduct problems scores, and as NoBaghira (*n* = 21) if their conduct problems score was higher than or equal to their emotional problems score. This is similar to the training decision in the initial interview, according to which children took part in the respective training appropriate for their main problem and the Baghira training was chosen more frequently in cases of uncertainty. This resulted in a total of *n* = 351 in the NoBaghira and *n* = 246 in the NoTiger group. Finally, *n* = 85 children were evaluated to have abnormal or clinically significant disruptive behavior or emotional problems or did not fulfill inclusion criteria for participation (Abnormal). As many families dropped out of the study after screening, children were excluded from the analyses if the respective questionnaire data for at least one sub-scale was missing at all measurement time points. Due to the study design, where only the SDQ was filled in during the screening and all other questionnaires were firstly administered at T1, the number of participants with data missing at all time points differed strongly for the SDQ (*n* = 6) and KINDL (*n* = 1504). Therefore, separate analyses were run for the SDQ (*n* = 2945 with *n* = 1928 Normal, *n* = 337 Training (*n* = 192 Baghira, *n* = 145 Tiger), *n* = 595 NoTraining (*n* = 350 NoBaghira, *n* = 245 NoTiger), *n* = 85 Abnormal) and KINDL data (*n* = 1441 with *n* = 907 Normal, *n* = 334 Training (*n* = 191 Baghira, *n* = 143 Tiger), *n* = 146 NoTraining (*n* = 83 NoBaghira, *n* = 63 NoTiger), *n* = 54 Abnormal). Due to the design of the PROMPt project with the screenings tied to the regular health check-ups at the pediatricians it happened that the same child entered the project twice as distinct cases in the data set. In this study’s analysis samples this occurred to four children: two children categorized once as Training and once as NoTraining and two children categorized twice as Normal. All four of them were included in the SDQ assigned group sample with both data entries. In the KINDL assigned group sample only one of the once Training and once NoTraining children was included with both entries and the other three with only one entry each.

### Prevention programs

The indicated prevention programs Baghira training and Tiger training were conducted at the TUD by certified trainers (psychologists or psychology master students).

#### Baghira training

The Baghira training (adapted from Aebi et al., [1] consisted of nine weekly 90 min group sessions for learning strategies for anger control and resolving conflicts appropriately. The different sessions covered content about emotion and self-awareness, dealing with anger and aggression, impulse control, conflict- and problem-solving, empathy, change of perspective and giving feedback. After introducing the topic of the session, the trainer helped the children practice the desired behavior in role plays. Further, the training included a reward program for encouraging the use of appropriate behavior. Additionally, children were to practice the learned strategies during the week. Adaptations to the original Baghira training contained slightly modified material to fit the younger target group (5–10 year olds instead of 8–13 year olds), reduction from 120 min to 90 min per session, inclusion of a short break and an added 90-minute information evening for parents. The latter was conducted concurrently to one of the training sessions to give parents information and advice on how to appropriately react in everyday situations and to help their child to handle anger and frustration.

#### Tiger training

The Tiger training [2] consisted of two one-on-one appointments with the child followed by nine weekly group sessions of 60 min each. In the former, the child got acquainted with the trainer and the shy hand puppet Til Tiger, who learned to become brave together with the child during the training. In the latter, the trainer and Til Tiger introduced different topics, e.g. doing something in front of others, rejecting something, making a justified demand, defending themselves against teasing without violence, which were then practiced in role plays. Furthermore, children were encouraged to practice learned strategies during the week. Additionally, a short version of progressive muscle relaxation was applied at the end of each session.

### Data analysis

Statistical analyses were carried out using the software STATA version 17.0 [44]. Descriptive statistics (number of participants, n; percent, %; mean, M; standard deviation, SD) were calculated for the SDQ screening results and the KINDL T0 data to describe the current status of disruptive behavior and emotional problems as well as quality of life among children from the general population using the pediatrician screening sample. To assess the association of disruptive behavior and emotional problems and quality of life, linear regression models were calculated for the KINDL score and the categorized SDQ result, adjusted for child’s sex and age. For both, the descriptive statistics of the SDQ and KINDL as well as the regression models, subjects who entered the project via other access routes than the screening at the pediatrician were excluded from these analyses, as they contacted the study team themselves with the need for prevention and might therefore bias the results. However, the results including these subjects can be found in the supplementary material. Further descriptive statistics were provided regarding socio-demographic and clinical characteristics of the analysis samples, separately for the assigned groups Normal, Training, NoTraining and Abnormal (based on the recommendation by the pediatrician after the screening with the SDQ and if any the initial interview) and the training groups Baghira, Tiger, NoBaghira and NoTiger. Baseline characteristics were compared using Chi-squared tests for categorical and t-tests or Kruskal-Wallis tests for metric variables. Post hoc Bonferroni-corrected Dunn’s tests of pairwise comparisons were carried out following significant Kruskal-Wallis tests.

To assess the effectiveness of indicated prevention programs, an intention-to-treat approach was applied. Linear mixed effect model regressions combined with the robust Huber/White/sandwich estimator were calculated for SDQ conduct and emotional problem scores and KINDL sub-scores focusing on the interactions measurement time point x assigned group in the total sample (pediatrician sample and other accession routes). Two models were calculated, using T0 and Normal (model 1) or Training (model 2) as references. Subjects were included as random effects, and childrens’ age and sex were taken into account as covariates. For disentangling possible effects by training group, equivalent models for the interactions measurement time point x training group were calculated. For better comparability of the outcomes and interpretability of beta coefficients as effect sizes regarding Cohen’s specification (≥ 0.20 small, ≥ 0.50 medium, ≥ 0.80 large effect; Cohen [11], , questionnaire scores were standardized by the pooled standard deviation by assigned group or training group at Screening (SDQ) or T0 (KINDL). The alpha level for all analyses was a priori set at 0.05. No correction for multiple testing regarding program effectiveness was applied. We decided to accept a 5% Type 1 error rate for each single test in exchange for a lower Type 2 error rate, thus favor sensitivity over robustness of findings in our observational study according with [39].

### Description and handling of missing data

Mixed effect model regressions are robust against missing data [47]. However, subjects with missing baseline values are omitted from calculation. In the current study, this would further reduce the number of subjects in the KINDL analyses due to missing data at T0 despite available data at T1 or T2. An overview of available data separately for the total sample and analyses samples by assigned group and training group (Table S1, S2, S3 and S4) as well as a comparison between children excluded vs. included in the KINDL analysis sample (Table S5) can be found in the supplementary material. As the T0 and T1 questionnaire assessments in the Training group took place at the TUD in combination with the training, dropouts were rare in this group. In contrast, families in the NoTraining and Abnormal group decided against training or sought help elsewhere, which often resulted in little interest in the further assessments and higher dropouts. Thus, we assumed that the missing pattern depended on the assigned group but not the KINDL baseline level and was therefore not assumed to be missing completely at random (MCAR), but rather missing at random (MAR). The performed Little’s Chi-squared test for the assumption MCAR was significant (Chi2 distance (919) = 1148.47, *p* < .001) and Little’s Chi-squared test for the assumption covariate-dependent missingness with overall group (assigned group and training group) as auxiliary variable was not significant (Chi2 distance (5514) = 2375.46, *p* = 1.00). This confirms that the missing pattern was not MCAR but MAR. Thus, we performed multiple imputation prior to the mixed model analyses. A comparison of sample characteristics between completer (data available at all three measurement time points) vs. non-completer (data available at least at one but not all three measurement time points) can be found in the supplementary material in Table S6 (SDQ analysis sample) and Table S7 (KINDL analysis sample). The multiple imputation model included SDQ emotional problems and conduct problems scores and all KINDL sub-scores at all measurement time points, child’s sex and age, as well as overall group as an auxiliary variable. The seed was randomly set to 7492. A-priori we selected 15 as number of imputations and conducted sensitivity analyses with up to 50 imputations. As these did not differ substantially, the number of imputations was retained for reasons of efficiency. Further, we also calculated the analyses without prior multiple imputation, which can be found in the supplementary material.

## Results

### Prevalence and quality of life

#### Sample characteristics

The sample of children screened at their pediatrician (without subjects who entered the project via other access routes) consisted of approximately equal proportions of boys (*n* = 1360, 48.1%) and girls (*n* = 1403, 49.7%) [sex of 62 children unknown] as well as younger children (3–6 year olds, *n* = 1439, 50.9%) and older children (7–11 year olds, *n* = 1386, 49.1%). Detailed characteristics of the whole PROMPt sample including the children with other accession routes can be found in Weniger et al. [51].

#### Prevalence of disruptive behavior and emotional problems

The prevalence of disruptive behavior and emotional problems in children from the general population as screened with the SDQ during pediatric health check-ups can be found in Table 1. Using SDQ cut-offs by Goodman [18], 13.0% showed borderline and 14.2% abnormal conduct problems scores with higher prevalence in boys and in younger children. Emotional problems with a borderline and abnormal score were found in 7.2% and 10.8%, respectively, with no significant sex differences but higher prevalence in older children. Overall, 37.0% of the children exhibited (borderline or abnormal) disruptive behavior and/or emotional problems; 63.0% of the children had normal scores in both SDQ sub-scales. Further, borderline emotional problems only were more prevalent in female than in male children, whereas borderline disruptive behavior problems only and abnormal scores were more frequent in male than in female children. Borderline disruptive behavior problems only were more common in younger children. In contrast, borderline emotional problems only, combined disruptive behavior and emotional problems and abnormal scores were more often exhibited in older children.

**Table 1:** Six-month prevalence of disruptive behavior and emotional problems in children from the general population^1^ using SDQ cut-offs by Goodman [18]

	Total		By child’s sex^2^				By child’s age			
	(*n* = 2825)		Female (*n* = 1403)		Male (*n* = 1360)		3–6 years (*n* = 1439)		7–11 years (*n* = 1386)	
	**n**	**%**	**n**	**%**	**n**	**%**	**n**	**%**	**n**	**%**
**SDQ conduct problems**										
Normal (score 0–2)	2055	72.7	1096	78.1	906	66.6	1016	70.6	1039	75.0
Borderline (score 3)	368	13.0	157	11.2	206	15.1	209	14.5	159	11.5
Abnormal (score 4–10)	402	14.2	150	10.7	248	18.2	214	14.9	188	13.6
			Chi2(2) = 48.12, *p* < .001				Chi2(2) = 7.74, *p* = .021			
**SDQ emotional problems**										
Normal (score 0–3)	2316	82.0	1126	80.3	1136	83.5	1232	85.6	1084	78.2
Borderline (score 4)	203	7.2	111	7.9	87	6.4	82	5.7	121	8.7
Abnormal (score 5–10)	306	10.8	166	11.8	137	10.1	125	8.7	181	13.1
			Chi2(2) = 5.06, *p* = .080				Chi2(2) = 26.21, *p* < .001			
**SDQ conduct problems and emotional problems combined**										
Normal (= normal in both scores)	1781	63.0	929	66.2	805	59.2	893	62.1	888	64.1
Borderline disruptive behavior problems only (= borderline conduct problems score and normal emotional problems score)	277	9.8	113	8.1	160	11.8	179	12.4	98	7.1
Borderline emotional problems only (= borderline emotional problems score and normal conduct problems score)	122	4.3	74	5.3	45	3.3	56	3.9	66	4.8
Borderline disruptive behavior & emotional problems (= borderline in both scores)	46	1.6	21	1.5	24	1.8	12	0.8	34	2.5
Abnormal (= abnormal in either one of the scores)	599	21.2	266	19.0	326	24.0	299	20.8	300	21.6
			Chi2(4) = 29.65, *p* < .001				Chi2(4) = 34.06, *p* < .001			

Note. ^1^Subjects who entered the project via other access routes than the screening at the pediatrician are excluded. ^2^Sex of 62 children unknown. SDQ = Strengths and Difficulties Questionnaire; n = number of participants; p = p-value

#### Quality of life

The KINDL mean scores by demographics and SDQ can be found in Table 2. Results of the regression models for the association of SDQ and KINDL scores adjusted for child’s sex and age are provided in the supplementary material. There were sex differences, with female children showing higher scores in self-esteem, family, friends and everyday functioning compared to male children, whereas the opposite was present for physical well-being. In contrast, emotional well-being scores did not differ between sexes. Additionally, younger children revealed higher quality of life scores than older children in all KINDL sub-scales except family, where scores did not differ by age group. Compared to children with normal SDQ scores, children with disruptive behavior and/or emotional problems exhibited lower quality of life scores in all KINDL sub-scales with two exceptions (see Table S14 in supplementary material): First, for the KINDL friends score, there were no differences between children with normal SDQ scores and borderline emotional problems only. Second, for the KINDL physical well-being, there were no differences between children with normal SDQ scores and children with both borderline disruptive behavior and emotional problems. Lowest quality of life scores were found in children with both borderline disruptive behavior and emotional problems and in children with abnormal SDQ scores. Except for the KINDL physical well-being and self-esteem scores, children with both borderline disruptive behavior and emotional problems exhibited even stronger negative associations with the KINDL scores than children with abnormal SDQ scores.

**Table 2:** Quality of life in children from the general population^1^

	Physical well-being		Emotional well-being		Self-esteem		Family		Friends		Everyday functioning	
	*n*	M (SD)	*n*	M (SD)	*n*	M (SD)	*n*	M (SD)	*n*	M (SD)	*n*	M (SD)
**Total sample**	1104	17.6 (2.17)	1102	17.0 (2.05)	1102	16.1 (2.30)	1102	16.8 (2.11)	1101	16.5 (2.39)	1092	17.0 (2.40)
**By child’s sex**												
Female	555	17.5 (2.26)	554	17.1 (2.07)	554	16.4 (2.24)	554	17.0 (2.04)	554	16.8 (2.24)	550	17.2 (2.39)
Male	544	17.8 (2.07)	543	16.9 (2.02)	543	15.8 (2.33)	543	16.6 (2.16)	542	16.2 (2.52)	537	16.7 (2.37)
	t(1097) = -2.67 *p* = .008		t(1095) = 1.29 *p* = .196		t(1095) = 4.33 *p* < .001		t(1095) = 3.49 *p* < .001		t(1094) = 3.93 *p* < .001		t(1085) = 3.82 *p* < .001	
**By age group**												
3–6 years	557	17.8 (2.08)	556	17.3 (1.90)	556	16.4 (2.02)	556	16.8 (2.03)	556	16.7 (2.16)	549	17.1 (2.41)
7–11 years	547	17.5 (2.25)	546	16.7 (2.15)	546	15.8 (2.52)	546	16.8 (2.20)	545	16.3 (2.60)	543	16.8 (2.37)
	t(1102) = 2.82 *p* = .005		t(1100) = 4.54 *p* < .001		t(1100) = 4.60 *p* < .001		t(1100) = -0.11 *p* = .911		t(1099) = 2.72 *p* = .007		(1090) = 2.57 *p* = .010	
**By SDQ screening result with cut-offs by Goodman** [18]**)**												
Normal	686	17.9 (1.97)	686	17.6 (1.66)	686	16.7 (2.00)	686	17.4 (1.83)	685	17.1 (2.10)	680	17.5 (2.16)
Borderline disruptive behavior problems only	84	17.5 (2.16)	84	17.0 (1.81)	84	16.0 (2.27)	84	16.3 (1.96)	84	16.4 (2.04)	84	16.8 (2.37)
Borderline emotional problems only	43	17.1 (2.09)	42	16.5 (1.57)	42	15.5 (1.85)	42	16.7 (1.75)	42	16.6 (1.82)	41	16.2 (2.49)
Borderline disruptive behavior & emotional problems	18	17.2 (2.36)	18	15.3 (1.88)	18	15.1 (2.41)	18	15.3 (1.97)	18	14.5 (3.85)	18	15.6 (2.55)
Abnormal	273	17.1 (2.51)	272	15.8 (2.42)	272	14.8 (2.45)	272	15.6 (2.30)	272	15.3 (2.64)	269	15.9 (2.55)
	Chi2(4) = 31.14 *p* < .001		Chi2(4) = 141.87 *p* < .001		Chi2(4) = 140.91 *p* < .001		Chi2(4) = 143.37 *p* < .001		Chi2(4) = 107.53 *p* < .001		Chi2(4) = 95.59 *p* < .001	

Note. KINDL scores were calculated as sum scores ranging from 0 to 20. Quality of life domains assessed with the Kiddy-KINDL-R and Kid-/Kiddo-KINDL-R Quality of Life Questionnaire for Children at T0. ^1^Subjects who entered the project via other access routes than the screening at the pediatrician are excluded. SDQ = Strengths and Difficulties Questionnaire; n = number of participants; M = mean; SD = standard deviation

### Effectiveness prevention program

#### Sample characteristics

The analysis for the indicated prevention program effectiveness was based on the assigned group (Normal, Training, NoTraining, Abnormal) following the SDQ screening during pediatric health check-ups (based on PROMPt project adapted cut-offs) including the clinical appraisal of the pediatrician and the initial interview. Additionally, subjects who entered the project via other access routes than the screening at the pediatrician were included. The characteristics of the assigned group are shown in Table 3. For additional information by training group (Baghira, Tiger, NoBaghira, NoTiger) see Table S16 in the supplementary material. There were more female children in Normal and more male children in Training and Abnormal respectively more boys in Baghira, Tiger and NoBaghira and more girls in NoTiger. Children in NoTraining were younger than children in Training. Also, children in NoBaghira were younger than the ones in Baghira. Further, children in the Normal group had the lowest symptom (SDQ) scores and highest quality of life (KINDL) scores. From analyses’ results and visual inspection, the Training group had mostly similar scores as Abnormal and worse scores than the NoTraining group.

**Table 3:** Characteristics of the assigned groups

	Normal		Training		NoTraining		Abnormal		Group comparison	
	n = 1928		n = 337		n = 595		n = 85			
	[n = 907]		[n = 334]		[n = 146]		[n = 54]			
	n	% / M (SD)	n	% / M (SD)	n	% / M (SD)	n	% / M (SD)	Chi2 (df)	p
**Child‘s sex **[%]										
Female	1011	53.75	112	33.23	283	48.71	30	35.71	55.66 (3)	< 0.001
	[490]	[54.26]	[110]	[32.93]	[72]	[49.66]	[20]	[37.04]	[47.29 (3)]	[< 0.001]
Male	870	46.25	225	66.77	298	51.29	54	64.29		
	[413]	[45.74]	[224]	[67.07]	[73]	[50.34]	[34]	[62.96]		
**Child’s age at screening**										
Mean age [M (SD)]	1922	6.71 (1.96)	330	6.72 (1.83)	586	6.52 (1.91)	85	7.33 (2.03)	12.53 (3)	0.006
	[906]	[6.73 (1.95)]	[327]	[6.71 (1.83)]	[145]	[6.28 (1.8)]	[54]	[7.37 (2.03)]	[12.3 (3)]	[0.006]
By age group [%]										
3–6 years	979	50.78	165	48.96	320	53.78	30	35.29	10.74 (3)	0.013
	[456]	[50.28]	[164]	[49.1]	[87]	[59.59]	[19]	[35.19]	[10.16 (3)]	[0.017]
7–11 years	949	49.22	172	51.04	275	46.22	55	64.71		
	[451]	[49.72]	[170]	[50.9]	[59]	[40.41]	[35]	[64.81]		
**SDQ screening result with cut-offs by Goodman** [18])^1^ [%]										
Normal	1712	88.98	24	7.34	49	8.25	4	4.71	2039.58 (12)	< 0.001
	[800]	[88.59]	[23]	[7.1]	[13]	[8.97]	[1]	[1.85]	[1014.99 (12)]	[< 0.001]
Borderline disruptive behavior problems only	95	4.94	28	8.56	150	25.25	8	9.41		
	[48]	[5.32]	[28]	[8.64]	[29]	[20]	[5]	[9.26]		
Borderline emotional problems only	26	1.35	22	6.73	77	12.96	5	5.88		
	[15]	[1.66]	[22]	[6.79]	[24]	[16.55]	[3]	[5.56]		
Borderline disruptive behavior & emotional problems	8	0.42	12	3.67	25	4.21	2	2.35		
	[4]	[0.44]	[12]	[3.7]	[3]	[2.07]	[2]	[3.7]		
Abnormal	83	4.31	241	73.7	293	49.33	66	77.65		
	[36]	[3.99]	[239]	[73.77]	[76]	[52.41]	[43]	[79.63]		
**SDQ screening result with PROMPt project adapted cut-offs**^1^ [%]										
Normal	1712	88.98	24	7.34	49	8.25	4	4.71	2150.21 (12)	< 0.001
	[800]	[88.59]	[23]	[7.1]	[13]	[8.97]	[1]	[1.85]	[1061.56 (12)]	[< 0.001]
Borderline disruptive behavior problems only	46	2.39	71	21.71	135	22.73	9	10.59		
	[23]	[2.55]	[71]	[21.91]	[40]	[27.59]	[4]	[7.41]		
Borderline emotional problems only	133	6.91	98	29.97	271	45.62	15	17.65		
	[66]	[7.31]	[97]	[29.94]	[57]	[39.31]	[8]	[14.81]		
Borderline disruptive behavior & emotional problems	22	1.14	57	17.43	77	12.96	13	15.29		
	[8]	[0.89]	[57]	[17.59]	[15]	[10.34]	[9]	[16.67]		
Abnormal	11	0.57	77	23.55	62	10.44	44	51.76		
	[6]	[0.66]	[76]	[23.46]	[20]	[13.79]	[32]	[59.26]		
**SDQ conduct problems at screening including subjects who entered project via other access routes **[M (SD)]	1924	1.08 (1.09)	327	3.31 (1.98)	594	2.92 (1.64)	85	4.3 (2.49)	854.06 (3)	< 0.001
	[903]	[1.07 (1.06)]	[324]	[3.31 (1.98)]	[145]	[2.87 (1.7)]	[54]	[4.43 (2.34)]	[444.84 (3)]	[< 0.001]
**SDQ conduct problems at screening without subjects who entered project via other access routes **[M (SD)]	1923	1.08 (1.09)	231	3.31 (1.9)	580	2.89 (1.58)	82	4.32 (2.53)	795.72 (3)	< 0.001
	[902]	[1.06 (1.05)]	[229]	[3.32 (1.9)]	[136]	[2.85 (1.63)]	[52]	[4.49 (2.37)]	[398.2 (3)]	[< 0.001]
**SDQ emotional problems at screening including subjects who entered project via other access routes **[M (SD)]	1924	1.19 (1.31)	327	3.99 (2.4)	594	3.07 (2.14)	85	3.86 (2.41)	714.21 (3)	< 0.001
	[903]	[1.22 (1.3)]	[324]	[3.99 (2.39)]	[145]	[3.28 (2.29)]	[54]	[4.12 (2.49)]	[414.91 (3)]	[< 0.001]
**SDQ emotional problems at screening without subjects who entered project via other access routes **[M (SD)]	1923	1.19 (1.31)	231	3.72 (2.32)	580	3.03 (2.1)	82	3.89 (2.41)	605.04 (3)	< 0.001
	[902]	[1.22 (1.3)]	[229]	[3.7 (2.31)]	[136]	[3.12 (2.15)]	[52]	[4.13 (2.51)]	[315.28 (3)]	[< 0.001]
**KINDL total score at T0**^1^ [M (SD)]	750	103.9 (7.69)	327	94.04 (9.25)	95	99.9 (9)	48	90.58 (11.82)	274.78 (3)	< 0.001
	[748]	[103.91 (7.69)]	[327]	[94.04 (9.25)]	[94]	[100.17 (8.65)]	[48]	[90.58 (11.82)]	[274.96 (3)]	[< 0.001]
**KINDL physical well-being**^1^ [M (SD)]	752	17.91 (1.99)	328	17.02 (2.39)	95	17.59 (2.24)	48	16.42 (2.74)	48.37 (3)	< 0.001
	[748]	[17.91 (2)]	[328]	[17.02 (2.39)]	[94]	[17.61 (2.25)]	[48]	[16.42 (2.74)]	[47.84 (3)]	[< 0.001]
**KINDL emotional well-being**^1^ [M (SD)]	751	17.56 (1.67)	327	15.61 (2.16)	95	16.79 (2.06)	48	15.27 (2.81)	202.54 (3)	< 0.001
	[748]	[17.56 (1.67)]	[327]	[15.61 (2.16)]	[94]	[16.84 (2.01)]	[48]	[15.27 (2.81)]	[202.18 (3)]	[< 0.001]
**KINDL self-esteem**^1^ [M (SD)]	751	16.68 (1.96)	327	14.68 (2.27)	95	15.97 (2.23)	48	14.19 (3.09)	189.45 (3)	< 0.001
	[748]	[16.69 (1.96)]	[327]	[14.68 (2.27)]	[94]	[16.02 (2.18)]	[48]	[14.19 (3.09)]	[190.17 (3)]	[< 0.001]
**KINDL family**^1^ [M (SD)]	751	17.31 (1.79)	327	15.57 (2.28)	95	16.48 (1.94)	48	15.15 (2.41)	163.67 (3)	< 0.001
	[748]	[17.3 (1.79)]	[327]	[15.57 (2.28)]	[94]	[16.49 (1.95)]	[48]	[15.15 (2.41)]	[162.24 (3)]	[< 0.001]
**KINDL friends**^1^ [M (SD)]	750	17 (2.07)	327	15.2 (2.6)	95	16.42 (2.47)	48	14.44 (3.2)	145.51 (3)	< 0.001
	[748]	[17.01 (2.07)]	[327]	[15.2 (2.6)]	[94]	[16.52 (2.28)]	[48]	[14.44 (3.2)]	[146.93 (3)]	[< 0.001]
**KINDL everyday functioning**^1^[M (SD)]	744	17.44 (2.14)	325	15.99 (2.56)	94	16.69 (2.3)	48	15.13 (2.79)	106.38 (3)	< 0.001
	[744]	[17.44 (2.14)]	[325]	[15.99 (2.56)]	[94]	[16.69 (2.3)]	[48]	[15.13 (2.79)]	[106.38 (3)]	[< 0.001]

Note. Sample characteristics are presented without parentheses for the whole sample, that equals the SDQ analysis sample, and in square brackets for the KINDL analysis sample

^1^Subjects who entered the project via other access routes than the screening at the pediatrician are included. SDQ = Strengths and Difficulties Questionnaire, KINDL = Kiddy-KINDL-R and Kid-/Kiddo-KINDL-R Quality of Life Questionnaire for Children, Normal = children evaluated as normal with no recommendation for indicated prevention participation; Training = children who participated in an indicated prevention program after recommendation, NoTraining = children who did not participate in an indicated prevention program despite recommendation; Abnormal = children with abnormal or clinically significant disruptive behavior or emotional problems or that did not fullfill inclusion criteria for participation in an indicated prevention program; n = sample size/number of participants; M = mean; SD = standard deviation; df = degrees of freedom; p = p-value

#### Effectiveness of prevention program participation

**Fig. 2:**
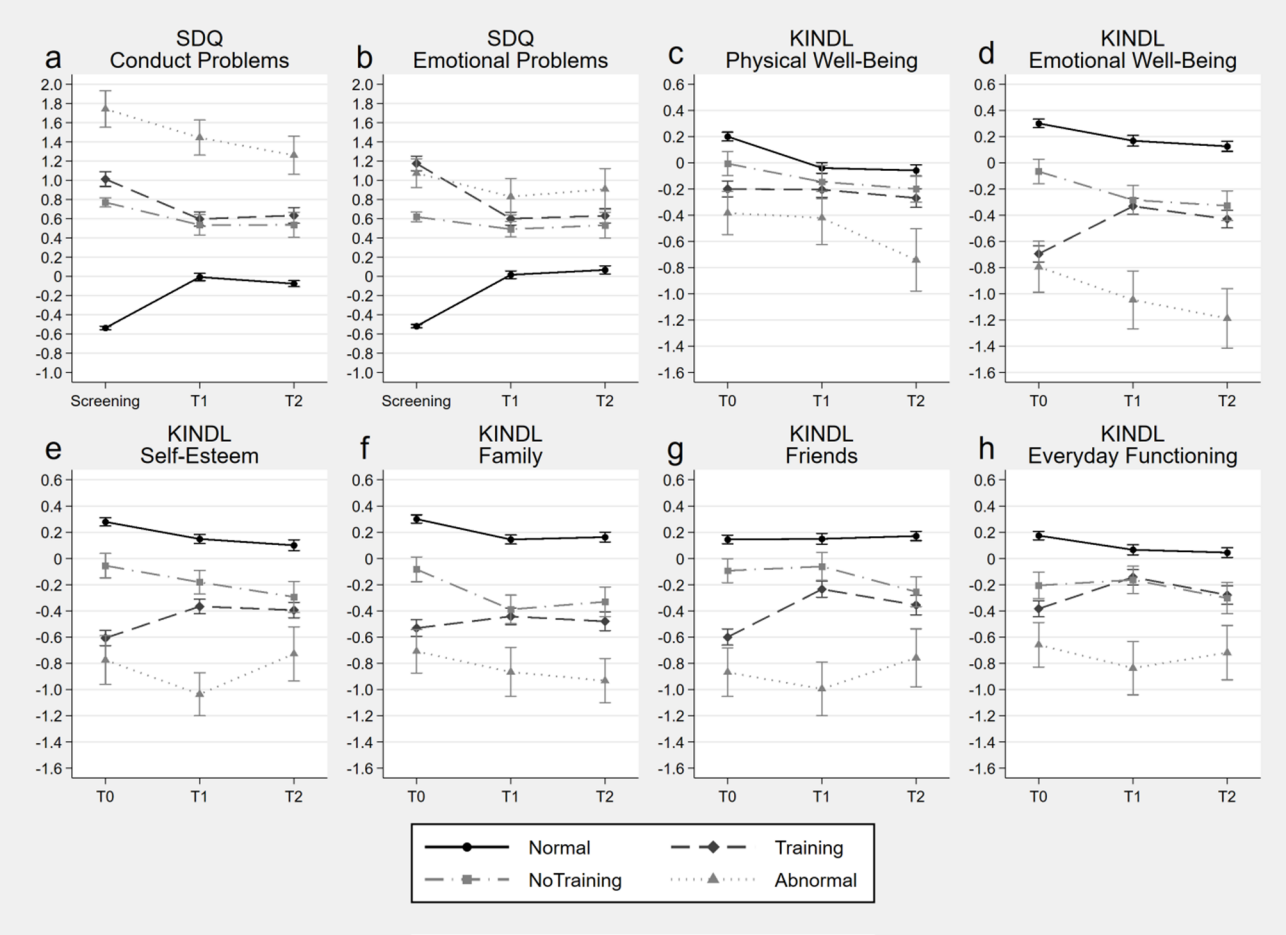
Estimated marginal means and standard errors based on the calculated mixed effect models for standardized measures by assigned group over the three assessment time points. SDQ = Strengths and Difficulties Questionnaire; KINDL = Kiddy-KINDL-R and Kid-/Kiddo-KINDL-R Quality of Life Questionnaire for Children; Normal = children evaluated as normal with no recommendation for indicated prevention participation; Training = children who participated in an indicated prevention program after recommendation; NoTraining = children who did not participate in an indicated prevention program despite recommendation; Abnormal = children with abnormal or clinically significant disruptive behavior or emotional problems or that did not fullfill inclusion criteria for participation in an indicated prevention program

**Fig. 3:**
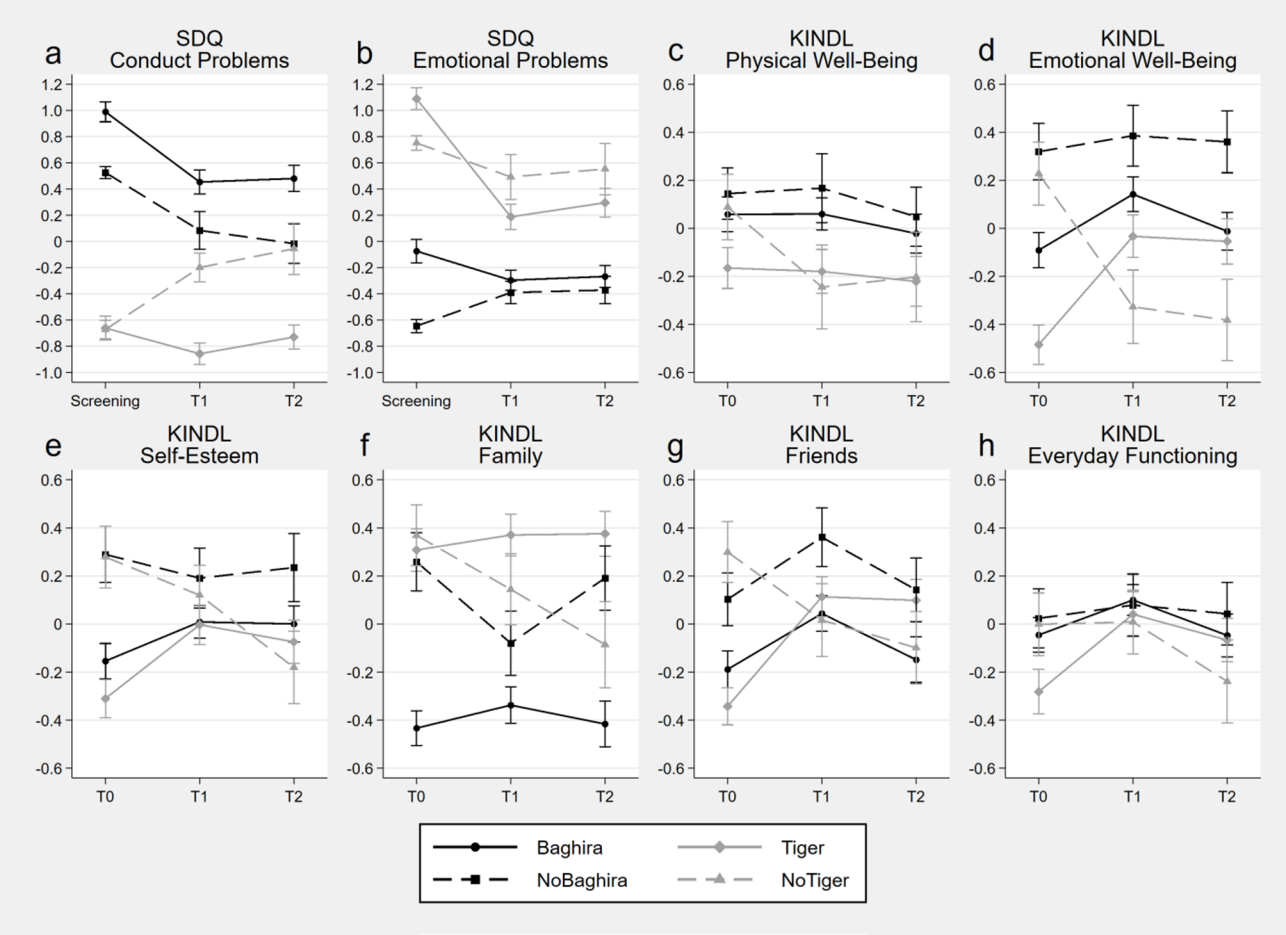
Estimated marginal means and standard errors based on the calculated mixed effect models for standardized measures by training group over the three assessment time points. SDQ = Strengths and Difficulties Questionnaire; KINDL = Kiddy-KINDL-R and Kid-/Kiddo-KINDL-R Quality of Life Questionnaire for Children; Baghira = children who participated in the Baghira training; Tiger = children who participated in the Tiger training; NoBaghira = children who did not participate in the Baghira training despite a recommendation including children with a recommendation for both trainings and a higher or equal SDQ conduct problems than emotional problems score; NoTiger = children who did not participate in the Tiger training despite a recommendation including children with a recommendation for both trainings and a higher SDQ emotional problems than conduct problems score

The results of the linear mixed effect models are shown in Table 4; Fig. 2 for analyses by assigned group and in Table S17 and Fig. 3 for the analyses by training group.

**Table 4:** Results of the calculated linear mixed effect models on timepoint by assigned group with multiple imputed data

	Model 1: Screening/T0 and Normal as reference						Model 2: Screening/T0 and Training as reference					
		β	SE	95% CI		*p*		β	SE	95% CI		*p*
**SDQ Conduct Problems**												
Measurement Time Point	T1	0.53	0.04	0.45	0.61	< 0.001	T1	-0.42	0.07	-0.55	-0.28	< 0.001
	T2	0.46	0.03	0.40	0.52	< 0.001	T2	-0.38	0.08	-0.53	-0.23	< 0.001
Group	Training	1.55	0.08	1.40	1.70	< 0.001	Normal	-1.55	0.08	-1.70	-1.40	< 0.001
	NoTraining	1.31	0.05	1.21	1.41	< 0.001	NoTraining	-0.24	0.09	-0.42	-0.07	0.007
	Abnormal	2.28	0.19	1.90	2.65	< 0.001	Abnormal	0.73	0.20	0.33	1.13	< 0.001
Time Point x Group	T1 x Training	-0.95	0.08	-1.10	-0.79	< 0.001	T1 x Normal	0.95	0.08	0.79	1.10	< 0.001
	T1 x NoTraining	-0.76	0.12	-1.01	-0.51	< 0.001	T1 x NoTraining	0.18	0.13	-0.08	0.44	0.168
	T1 x Abnormal	-0.83	0.18	-1.18	-0.48	< 0.001	T1 x Abnormal	0.12	0.19	-0.25	0.49	0.531
	T2 x Training	-0.84	0.09	-1.01	-0.67	< 0.001	T2 x Normal	0.84	0.09	0.67	1.01	< 0.001
	T2 x NoTraining	-0.69	0.13	-0.96	-0.43	< 0.001	T2 x NoTraining	0.14	0.15	-0.16	0.45	0.344
	T2 x Abnormal	-0.94	0.19	-1.33	-0.56	< 0.001	T2 x Abnormal	-0.10	0.21	-0.51	0.31	0.616
**SDQ Emotional Problems**												
Measurement Time Point	T1	0.53	0.04	0.45	0.62	< 0.001	T1	-0.57	0.07	-0.71	-0.44	< 0.001
	T2	0.59	0.04	0.50	0.67	< 0.001	T2	-0.54	0.08	-0.70	-0.39	< 0.001
Group	Training	1.69	0.08	1.54	1.85	< 0.001	Normal	-1.69	0.08	-1.85	-1.54	< 0.001
	NoTraining	1.14	0.05	1.03	1.24	< 0.001	NoTraining	-0.56	0.09	-0.74	-0.38	< 0.001
	Abnormal	1.59	0.15	1.30	1.89	< 0.001	Abnormal	-0.10	0.17	-0.43	0.23	0.548
Time Point x Group	T1 x Training	-1.11	0.08	-1.27	-0.95	< 0.001	T1 x Normal	1.11	0.08	0.95	1.27	< 0.001
	T1 x NoTraining	-0.66	0.09	-0.85	-0.48	< 0.001	T1 x NoTraining	0.45	0.11	0.24	0.66	< 0.001
	T1 x Abnormal	-0.78	0.19	-1.15	-0.41	< 0.001	T1 x Abnormal	0.33	0.20	-0.06	0.72	0.097
	T2 x Training	-1.13	0.09	-1.32	-0.94	< 0.001	T2 x Normal	1.13	0.09	0.94	1.32	< 0.001
	T2 x NoTraining	-0.67	0.16	-1.00	-0.34	< 0.001	T2 x NoTraining	0.46	0.16	0.14	0.78	0.007
	T2 x Abnormal	-0.75	0.22	-1.20	-0.30	0.001	T2 x Abnormal	0.38	0.23	-0.08	0.84	0.104
**KINDL Physical Well-Being**												
Measurement Time Point	T1	-0.24	0.05	-0.34	-0.14	< 0.001	T1	0.00	0.06	-0.13	0.12	0.940
	T2	-0.26	0.05	-0.36	-0.15	< 0.001	T2	-0.07	0.08	-0.22	0.09	0.380
Group	Training	-0.40	0.07	-0.54	-0.26	< 0.001	Normal	0.40	0.07	0.26	0.54	< 0.001
	NoTraining	-0.21	0.10	-0.39	-0.02	0.030	NoTraining	0.19	0.11	-0.02	0.41	0.078
	Abnormal	-0.58	0.17	-0.92	-0.25	0.001	Abnormal	-0.18	0.18	-0.53	0.16	0.296
Time Point x Group	T1 x Training	0.24	0.08	0.09	0.39	0.002	T1 x Normal	-0.24	0.08	-0.39	-0.09	0.002
	T1 x NoTraining	0.10	0.14	-0.18	0.38	0.471	T1 x NoTraining	-0.13	0.14	-0.42	0.15	0.347
	T1 x Abnormal	0.20	0.24	-0.27	0.68	0.400	T1 x Abnormal	-0.03	0.24	-0.51	0.44	0.892
	T2 x Training	0.19	0.09	0.00	0.38	0.045	T2 x Normal	-0.19	0.09	-0.38	0.00	0.045
	T2 x NoTraining	0.07	0.13	-0.19	0.33	0.620	T2 x NoTraining	-0.12	0.14	-0.40	0.15	0.379
	T2 x Abnormal	-0.10	0.25	-0.59	0.40	0.692	T2 x Abnormal	-0.29	0.26	-0.80	0.22	0.266
**KINDL Emotional Well-Being**												
Measurement Time Point	T1	-0.13	0.05	-0.22	-0.04	0.005	T1	0.36	0.07	0.23	0.50	< 0.001
	T2	-0.17	0.04	-0.26	-0.09	< 0.001	T2	0.27	0.07	0.12	0.41	< 0.001
Group	Training	-1.00	0.07	-1.13	-0.86	< 0.001	Normal	1.00	0.07	0.86	1.13	< 0.001
	NoTraining	-0.37	0.10	-0.56	-0.18	< 0.001	NoTraining	0.63	0.11	0.41	0.85	< 0.001
	Abnormal	-1.09	0.20	-1.48	-0.71	< 0.001	Abnormal	-0.10	0.20	-0.50	0.30	0.629
Time Point x Group	T1 x Training	0.50	0.08	0.34	0.65	< 0.001	T1 x Normal	-0.50	0.08	-0.65	-0.34	< 0.001
	T1 x NoTraining	-0.09	0.12	-0.33	0.16	0.487	T1 x NoTraining	-0.58	0.14	-0.85	-0.31	< 0.001
	T1 x Abnormal	-0.12	0.22	-0.55	0.31	0.579	T1 x Abnormal	-0.62	0.22	-1.05	-0.18	0.006
	T2 x Training	0.44	0.09	0.27	0.61	< 0.001	T2 x Normal	-0.44	0.09	-0.61	-0.27	< 0.001
	T2 x NoTraining	-0.09	0.13	-0.35	0.18	0.518	T2 x NoTraining	-0.53	0.15	-0.81	-0.24	< 0.001
	T2 x Abnormal	-0.22	0.19	-0.59	0.15	0.247	T2 x Abnormal	-0.66	0.20	-1.05	-0.27	0.001
**KINDL Self-Esteem**												
Measurement Time Point	T1	-0.13	0.04	-0.20	-0.06	0.001	T1	0.24	0.06	0.12	0.36	< 0.001
	T2	-0.18	0.04	-0.26	-0.09	< 0.001	T2	0.21	0.06	0.09	0.33	0.001
Group	Training	-0.89	0.07	-1.02	-0.76	< 0.001	Normal	0.89	0.07	0.76	1.02	< 0.001
	NoTraining	-0.33	0.10	-0.52	-0.15	0.001	NoTraining	0.55	0.11	0.33	0.77	< 0.001
	Abnormal	-1.06	0.19	-1.43	-0.68	< 0.001	Abnormal	-0.17	0.19	-0.55	0.21	0.386
Time Point x Group	T1 x Training	0.37	0.07	0.24	0.51	< 0.001	T1 x Normal	-0.37	0.07	-0.51	-0.24	< 0.001
	T1 x NoTraining	0.00	0.12	-0.23	0.24	0.971	T1 x NoTraining	-0.37	0.13	-0.63	-0.11	0.006
	T1 x Abnormal	-0.13	0.18	-0.48	0.22	0.468	T1 x Abnormal	-0.50	0.19	-0.87	-0.13	0.008
	T2 x Training	0.39	0.08	0.24	0.54	< 0.001	T2 x Normal	-0.39	0.08	-0.54	-0.24	< 0.001
	T2 x NoTraining	-0.06	0.15	-0.35	0.23	0.683	T2 x NoTraining	-0.45	0.15	-0.76	-0.15	0.005
	T2 x Abnormal	0.23	0.22	-0.20	0.65	0.300	T2 x Abnormal	-0.17	0.21	-0.58	0.25	0.433
**KINDL Family**												
Measurement Time Point	T1	-0.16	0.04	-0.23	-0.09	< 0.001	T1	0.09	0.06	-0.03	0.21	0.157
	T2	-0.14	0.04	-0.21	-0.06	< 0.001	T2	0.05	0.06	-0.07	0.18	0.426
Group	Training	-0.83	0.07	-0.97	-0.69	< 0.001	Normal	0.83	0.07	0.69	0.97	< 0.001
	NoTraining	-0.38	0.10	-0.58	-0.19	< 0.001	NoTraining	0.45	0.12	0.22	0.68	< 0.001
	Abnormal	-1.01	0.17	-1.34	-0.68	< 0.001	Abnormal	-0.18	0.18	-0.53	0.17	0.319
Time Point x Group	T1 x Training	0.25	0.07	0.10	0.39	0.001	T1 x Normal	-0.25	0.07	-0.39	-0.10	0.001
	T1 x NoTraining	-0.15	0.14	-0.43	0.13	0.288	T1 x NoTraining	-0.39	0.15	-0.70	-0.09	0.013
	T1 x Abnormal	0.00	0.20	-0.41	0.40	0.993	T1 x Abnormal	-0.25	0.21	-0.66	0.17	0.242
	T2 x Training	0.19	0.07	0.04	0.33	0.011	T2 x Normal	-0.19	0.07	-0.33	-0.04	0.011
	T2 x NoTraining	-0.11	0.12	-0.36	0.14	0.383	T2 x NoTraining	-0.30	0.14	-0.57	-0.03	0.030
	T2 x Abnormal	-0.09	0.19	-0.45	0.28	0.643	T2 x Abnormal	-0.28	0.19	-0.65	0.10	0.152
**KINDL Friends**												
Measurement Time Point	T1	0.01	0.04	-0.08	0.09	0.906	T1	0.37	0.07	0.24	0.49	< 0.001
	T2	0.03	0.04	-0.05	0.11	0.508	T2	0.24	0.08	0.09	0.40	0.002
Group	Training	-0.74	0.07	-0.88	-0.61	< 0.001	Normal	0.74	0.07	0.61	0.88	< 0.001
	NoTraining	-0.24	0.10	-0.43	-0.04	0.016	NoTraining	0.51	0.11	0.29	0.72	< 0.001
	Abnormal	-1.01	0.19	-1.38	-0.65	< 0.001	Abnormal	-0.27	0.19	-0.65	0.11	0.167
Time Point x Group	T1 x Training	0.36	0.08	0.20	0.52	< 0.001	T1 x Normal	-0.36	0.08	-0.52	-0.20	< 0.001
	T1 x NoTraining	0.03	0.12	-0.22	0.27	0.829	T1 x NoTraining	-0.33	0.14	-0.61	-0.06	0.018
	T1 x Abnormal	-0.13	0.20	-0.53	0.26	0.511	T1 x Abnormal	-0.49	0.21	-0.90	-0.08	0.019
	T2 x Training	0.22	0.08	0.05	0.38	0.009	T2 x Normal	-0.22	0.08	-0.38	-0.05	0.009
	T2 x NoTraining	-0.19	0.15	-0.49	0.11	0.210	T2 x NoTraining	-0.41	0.15	-0.71	-0.10	0.009
	T2 x Abnormal	0.08	0.19	-0.29	0.46	0.665	T2 x Abnormal	-0.13	0.20	-0.53	0.26	0.507
**KINDL Everyday Functioning**												
Measurement Time Point	T1	-0.11	0.04	-0.20	-0.02	0.016	T1	0.24	0.07	0.11	0.38	0.001
	T2	-0.13	0.04	-0.21	-0.05	0.002	T2	0.10	0.08	-0.05	0.26	0.188
Group	Training	-0.56	0.07	-0.70	-0.42	< 0.001	Normal	0.56	0.07	0.42	0.70	< 0.001
	NoTraining	-0.38	0.11	-0.60	-0.16	0.001	NoTraining	0.18	0.12	-0.06	0.41	0.136
	Abnormal	-0.83	0.17	-1.17	-0.49	< 0.001	Abnormal	-0.28	0.18	-0.63	0.08	0.125
Time Point x Group	T1 x Training	0.35	0.08	0.19	0.51	< 0.001	T1 x Normal	-0.35	0.08	-0.51	-0.19	< 0.001
	T1 x NoTraining	0.15	0.13	-0.10	0.40	0.239	T1 x NoTraining	-0.20	0.14	-0.48	0.08	0.160
	T1 x Abnormal	-0.07	0.21	-0.49	0.35	0.740	T1 x Abnormal	-0.42	0.22	-0.85	0.01	0.055
	T2 x Training	0.23	0.09	0.06	0.41	0.008	T2 x Normal	-0.23	0.09	-0.41	-0.06	0.008
	T2 x NoTraining	0.03	0.17	-0.30	0.37	0.846	T2 x NoTraining	-0.20	0.19	-0.59	0.19	0.299
	T2 x Abnormal	0.07	0.21	-0.34	0.48	0.736	T2 x Abnormal	-0.16	0.23	-0.61	0.28	0.468

Note. SDQ = Strengths and Difficulties Questionnaire, KINDL = Kiddy-KINDL-R and Kid-/Kiddo-KINDL-R Quality of Life Questionnaire for Children, Normal = children evaluated as normal with no recommendation for indicated prevention participation; Training = children who participated in an indicated prevention program after recommendation, NoTraining = children who did not participate in an indicated prevention program despite recommendation; Abnormal = children with abnormal or clinically significant disruptive behavior or emotional problems or that did not fullfill inclusion criteria for participation in an indicated prevention program; β = beta coefficient; SE = standard error; CI = confidene interval; p = p-value

*Effectiveness on Symptomatology* For the models for SDQ conduct problems (Fig. 2a), there was a symptom increase in the Normal group from Screening to T1 and Screening to T2 in contrast to a symptom decrease in all of the other assigned groups. Also, there were no differences in symptom reduction between the groups Training vs. NoTraining and Abnormal. In fact, there were interaction effects for Normal vs. Training, Normal vs. NoTraining and Normal vs. Abnormal by timepoints Screening to T1 and Screening to T2, but not for Training vs. NoTraining and Training vs. Abnormal. Further, the models by training group demonstrated interaction effects for Baghira vs. Tiger and Tiger vs. No Tiger by timepoint Screening to T1 and Screening to T2, but no interaction effect Baghira vs. NoBaghira by time point. It shows that the Baghira group exhibited a symptom reduction which was stronger than the one in the Tiger group, but similar to the NoBaghira group. Further, in contrast to the Tiger group, the SDQ conduct problems score increased in the NoTiger group from Screening to T1 and T2 (Fig. 3a).

For the model for SDQ emotional problems (Fig. 2b), there were interaction effects with Normal vs. Training, Normal vs. NoTraining, Normal vs. Abnormal and Training vs. NoTraining for both time points Screening to T1 and Screening to T2, but no interaction effects Training vs. Abnormal by timepoints. The Normal group showed a symptom increase whereas the Training and Abnormal groups showed a symptom reduction and the NoTraining group did not change. In addition, the symptom reduction from Screening to T1 and T2 was greater in the Training group compared to the NoTraining group and similar between Training and Abnormal. Also, we see interaction effects for Baghira vs. Tiger, Baghira vs. NoBaghira and Tiger vs. NoTiger by timepoint Screening to T1 and Screening to T2. Results showed a stronger symptom reduction in the Tiger group than in the NoTiger and the Baghira group and, in contrast, a symptom increase in the NoBaghira group (Fig. 3b).

*Effectiveness on Quality of Life* In all KINDL scales there were interaction effects for Normal vs. Training by T0 to T1 and T0 to T2. The Normal group decreased or did not change (KINDL friends) in the KINDL scores. In contrast, the Training group did not change (KINDL physical well-being) or increased in the KINDL scores. However, in all KINDL sub-scales, there were no interaction effects for Normal vs. NoTraining and Abnormal by any time point. That is, these groups did not differ in their change of the KINDL scores over time. Further, there were interaction effects Training vs. NoTraining by T0 to T1 and T0 to T2 for all KINDL sub-scales besides physical well-being and everyday functioning. They revealed an increase in quality of life in the Training group compared to a decrease or no change in the NoTraining group. For the contrast Training vs. Abnormal, there were only interaction effects for KINDL emotional well-being T0 to T1 and T0 to T2, self-esteem T0 to T1 and friends T0 to T1. At these, the quality of life scores decreased in the Abnormal group whereas the scores of the Training group improved. The results by training group revealed an interaction effect Baghira vs. Tiger from T0 to T2 for emotional well-being and friends, with the groups improving equally from T0 to T1, but the Tiger group showing greater improvement from T0 to T2, because the scores of the Baghira group returned to almost the T0 level from T1 to T2. In the other scores change, the Baghira and Tiger group did not differ. Regarding the contrast Baghira vs. NoBaghira, there was only one interaction effect for the KINDL family score from T0 to T1, where the NoBaghira group’s score decreased and the Baghira group’s score slightly increased. However, there were no differences in the quality of life score’s change between the Baghira and NoBaghira group in the other KINDL sub-scales. Regarding the contrast Tiger vs. NoTiger, there were interaction effects for emotional well-being, self-esteem and friends for T0 to T1 and T0 to T2 and family for T0 to T2, where the quality of life scores improved in the Tiger group, while they deteriorated in the NoTiger group.

## Discussion

The current study aimed (1) to provide prevalence estimates of disruptive behavior and emotional problems and their association with quality of life among children from the general population as screened during routine pediatric health check-ups and (2) to examine the effectiveness of two indicated child-based group prevention programs.

### Prevalence of disruptive behavior and emotional problems and association with quality of life

The prevalence estimates of disruptive behavior and emotional problems as screened using the SDQ as well as their age and sex distributions are comparable to previous studies in children from the general population in Germany (e.g. Hölling et al., [22]; Hölling et al., [23]. In contrast to Hölling et al. [22], our sample only yielded slightly higher rates of abnormal emotional problems scores and slightly lower rates of borderline conduct problems scores. These results, especially considering our high overall response rate at screening (86.4%), verify the high prevalence of disruptive behavior and emotional problems among children in the general population, both on a subclinical (borderline) and clinically-relevant (abnormal) level, indicating the need for early recognition and intervention efforts. The age differences with borderline disruptive behavior only being more frequent in younger children and borderline emotional problems, co-occurring borderline disruptive behavior and emotional problems and abnormal SDQ scores being more frequent in older children are in line with literature showing a later age of onset of emotional problems compared to disruptive behavior problems [24, 28], higher risks for subsequent co-occurring conditions in subjects with subthreshold mental health problems [15], as well as studies showing transitions of subthreshold to clinically relevant symptom expressions over time [8, 15]. The age-span between 5 and 10 years can thus be considered a risk phase for first emergence and early progression of psychopathology. This time window is therefore suitable for indicated prevention and early intervention.

The descriptive statistics of the KINDL scores reveal similar results as found by Ravens-Sieberer et al. [36] in a normative sample of German children. We found lower quality of life scores in older children compared to younger children matching Ravens-Sieberer et al. [36, 42], as well as lower physical well-being in girls compared to boys like Ravens-Sieberer et al. [36]. Additionally, we found sex differences for the other KINDL sub-scales besides emotional well-being, with higher scores in girls than boys, which was not found by Ravens-Sieberer et al. [36]. The results of the analysis examining the association of SDQ and KINDL scores confirm the hypothesis that quality of life is reduced in children with disruptive behavior or emotional problems, which is evident in all quality of life domains of the KINDL. This is in line with previous work and even expands it: Ravens-Sieberer et al. [36] found that quality of life is reduced in children with borderline SDQ scores and even further reduced in children with abnormal scores. Sharpe et al. [42] evidenced that children with co-occurring disruptive behavior and emotional problems exhibit lower quality of life than children with either of those problems alone. This current study implies an additive effect of co-occurring disruptive behavior and emotional problems, which already occurs on the subclinical symptom level. In fact, children with co-occurring borderline disruptive behavior and emotional problems mostly demonstrate greater reduced quality of life scores than children with abnormal SDQ scores. Along with arising mental health problems, these findings further emphasize the importance of (indicated) prevention.

### Effectiveness of indicated prevention program participation on symptomatology and quality of life

As hypothesized, we found decreases in the symptomatology and increases in the quality of life in children who participated in the indicated prevention program. Unexpectedly, non-participating children who had been screened as normal or abnormal and children not participating despite a prevention indication also exhibited changes over time. Children screened as normal showed an increase in disruptive behavior and emotional problems. Given the low SDQ scores of this group at screening, this could be explained by regression to the mean, increased awareness through the project, or – more likely – by the fact that first incidence of disruptive behavior and emotional problems increases around the time of the sample’s age range (Normal group’s age at screening: M = 6.71 SD = 1.96) [24, 28]. Additionally, adverse effects of the Corona pandemic [38] may have contributed to this deterioration. The analogous slight decreases of quality of life scores in the Normal group are plausible given its association with symptomatology. Also, other studies found symptomatology increases as predictor for quality of life and its changes [33]. Nevertheless, the Normal group’s symptomatology scores stay below and quality of life scores stay above the scores of the other assigned groups at all measurement time points. The Abnormal group shows a different course than the Normal group. On the one hand, the symptomatology decreases, which can be due to spontaneous improvements or improvements through treatment endorsed by the study design. On the other hand, the quality of life scores do not change or decrease either, especially in physical and emotional well-being. Despite the symptom decrease, the Abnormal group still exhibits the highest SDQ scores and symptom decrease seems not sufficient to counteract the adverse effects on the quality of life resulting in keeping the low quality of life scores. Besides insufficient improvement in symptomatology, this could also be due to long-term deterioration of quality of life, persistent impairments, or additional factors not included in our analyses, e.g. parental stress that impact the quality of life. Children in the Training group show significantly greater symptom reduction in emotional problems than the ones in NoTraining with effect sizes of β = 0.45 at for Screening to T1 and β = 0.46 for Screening to T2. Additionally, children of the Training group improve in almost all quality of life dimensions whereas NoTraining children decrease in their scores with small to medium effect sizes (β = 0.30–0.58). This demonstrates that participation in the indicated prevention offered was effective. However, our differentiated analyses showed that the results for the two indicated prevention programs Baghira training and Tiger training differ. The improvements in the Tiger group compared to the worsening in the NoTiger group with predominantly medium to large effect sizes (β = 0.47–1.04) demonstrate the effectiveness of the Tiger training as an indicated prevention program, which is in line with Ahrens-Eipper et al. [2, 54]. In contrast, there are barely interaction effects between the groups Baghira and NoBaghira, albeit some improvements, especially in the emotional problems symptomatology (effect sizes of β = 0.48 at for Screening to T1 and β = 0.47 for Screening to T2), are present in the Baghira group. These differences in effectiveness between the two indicated prevention programs might be due to differences in program content. Also, differences in allocation to the Tiger or Baghira training could have occurred. For example, the Baghira training might have been recommended more often for children whose symptoms would improve by themselves over time and families correctly estimated that the training participation was not necessary. Fittingly, one of the main reasons for refusal of training participation was “no demand/necessity” [51]. However, the Baghira group improved as expected and the contradictory part of the results concerns the NoBaghira group. The question arises as to why the NoBaghira group improves to a similar extent as the Baghira group. A possible answer could be the non-randomized design of the study, which could be linked to two possible biases. First, the children in the Training group exhibited worse (i.e. more unfavorable) initial scores at Screening/T0 than the NoTraining group. Moreover, the initial scores of the Training group were more similar to those of the Abnormal than to those of the NoTraining group. This might be due to the fact that higher symptom severity is associated with a higher level of parental problem recognition and help-seeking behavior [10]. As there is mixed evidence on the role of initial symptom severity on intervention effects [31], and given that individuals with lower symptom expressions are more likely to naturalistically improve than those with higher symptom scores [53], it seems plausible that differences between the groups do not become visible in this non-randomized study design. Additionally, regression to the mean could account for part of the symptom reduction. Albeit, this would also be true for the NoTraining group and calculated percent of regression to the mean are similar or even slightly higher in the NoTraining group. However, the role of these effects regarding the Baghira and Tiger training as well as specific domains and overall quality of life needs further investigation. Second, although intervention offers during the Corona pandemic were rare, we cannot entirely rule out that children of the NoTraining group participated in other interventions and could thus also exhibit intervention effects. Albeit, it is unlikely that this concerns a large proportion of the NoTraining group, as many families refused to participate due to “no demand/necessity” [51]. Hence, for now we cannot prove the effectiveness of the Baghira training as an indicated prevention program for disruptive behavior. Yet, its effectiveness might nevertheless be given, because the Baghira group exhibited improvements over the course of the training. Also, effectiveness of the Baghira training in combination with the Triple-P-parent training for clinical cases was shown by Wettach and Aebi [52]. However, as the improvement cannot clearly be attributed to the training participation further research is needed to determine its effectiveness. Additionally, the result regarding the SDQ emotional problems scale should be discussed. Here, we find the Baghira training to be effective as emotional problem symptomatology decreases in the Baghira group but increases in the NoBaghira group. The former could be explained by the fact that the Baghira training addresses factors that are also shared with emotional problems like peer processes, biases in information processing and attentional control. The latter could be due to longitudinal progression from early disruptive behavior to anxiety [28].

In summary, findings of this current study indicate a high prevalence of disruptive behavior and emotional problems in children from the general population and links to impaired quality of life. Identification of such problems, e.g. via systematic screenings in routine health care, and allocation to indicated prevention is promising for improving public health, given that preventive program participation effectively reduces symptomatology and increases quality of life. This could be clearly demonstrated in the current study for the Tiger training. Regarding the Baghira training, effectiveness is implicated, but further research is needed.

### Limitations

The current study is not without limitations. First, this implementation study followed an observational naturalistic design. Therefore, conclusions can only be drawn about the effectiveness, but not about the efficacy of the indicated prevention programs which would require a randomized controlled design. In addition, as no randomization was employed, the Training and NoTraining groups differed in initial symptom levels, impairing conclusions on effectiveness. However, the study adds important findings regarding the course of symptomatology and quality of life in children with or without preventive program participation under ecologically valid conditions. Second, almost all trainings and questionnaire evaluations took place during the Corona pandemic and its effects on the results cannot be determined. Although, Gorbeña et al. [21] demonstrated that an intervention to improve mental health was as good during the Corona pandemic as before the pandemic, adverse effects on the effectiveness of the Baghira and Tiger training cannot be excluded. The pandemic had a great burden on mental health and quality of life in children and parents [38]. The great distress might have impaired the training effects and contact restrictions might have hindered practicing the program content. Also, adjustments in the indicated prevention programs were necessary, e.g. smaller groups and exercises without physical contact. Third, questionnaire data were derived from one parent (or caregiver). The SDQ and KINDL each show medium correlations between their parent- and self-report versions [17, 20]. Thus, results could slightly differ when using self-report data. Future research should additionally consider if the child itself perceived changes through training as well as clinical evaluations of the symptomatology. Fourth, although a self-reported current emotional or disruptive behavior ICD-10 diagnosis was an exclusion criterion for program participation, we cannot rule out the possibility that children with clinical disorders participated since the initial interview did not include a diagnostic interview. Fifth, results might not be transferable to other regions and cultures, although the sample can be considered as representative for the German population (urban areas) of children aged 5–10 years since the participation rates for the regular health check-ups at the pediatricians are high [40] and the response rate of the current study was also high (86.4%). Yet, families with low socioeconomic status or of non-German nationality are likely slightly underrepresented, as they show somewhat lower participation rates in these regular health check-ups [40], and families with insufficient knowledge of the German language were indirectly excluded from the current study, given that the study was conducted with German study materials only. Although, study groups of the sample did not differ regarding these characteristics, prevalences might have been somewhat higher, as literature shows that they often belong to the risk group for mental health problems and need for prevention [4]. Sixth, in this longitudinal study, there were high dropout rates and missing values in the data set, especially in the NoTraining and Abnormal groups. Hence, we analyzed the missing pattern and performed multiple imputation. The results of the mixed models with and without prior multiple imputation are comparable and suggest reliable findings. Of note, very high retention was observed in the Training group. Seventh, no correction for multiple testing regarding program effectiveness was applied. Thus, results should be interpreted with caution and confirmed in further research.

## Conclusion

In sum, disruptive behavior and emotional problems are frequent in children and are associated with impaired quality of life. Indicated child-based prevention programs are able to reduce symptomatology and strengthen quality of life in affected children which possibly counteracts the development of full-blown disorders. Regular screening in routine pediatric health check-ups might be a viable way for early identification of children who might profit from participation in indicated prevention programs. Thus, decreases in the incidence of psychopathology and improvements in quality of life could be achieved on a population level in the future. However, considerably work is needed to determine which indicated prevention offers are particularly promising to achieve such important goal. The current study evidenced that the Tiger training, which primarily addresses emotional problems/anxiety, is effective. For the Baghira training, primarily addressing disruptive behavior problems, preliminary beneficial effects were shown, but due to the missing differences in trajectories compared to NoBaghira, further randomized controlled evaluations are needed to determine whether it is truly effective. Further research should also examine the longitudinal course of children with emotional and especially disruptive behavior problems to reliably identify children at risk for an unfavorable course to improve prediction and allocation to indicated prevention programs. The implementation of screenings during the regular health check-ups and recommendation of participation in prevention programs seems substantiated. Thus, future research should further evaluate the best practice for that in combination with controlled evaluations which indicated prevention programs are most efficacious and effective for which risk group.

## Supplementary Information

Below is the link to the electronic supplementary material.

Supplementary Material 1

## Data Availability

Availability of data and materials: The data supporting the findings of this study are available from the senior author upon reasonable request.

## Abbreviations

Abnormal: Children with abnormal or clinically significant emotional or disruptive behavior problems or that did not fullfill inclusion criteria for participation in a prevention program
b: Regression coefficient
Baghira: children who participated in the Baghira training
Baghira training: Indicated group program for children with disruptive behavior problems, adapted from the Baghira group training
CI: Confidence interval
df: Degrees of freedom
KINDL/KINDL-R/Kiddy-KINDL-R/Kid-/Kiddo-KINDL-R: Revidierter Fragebogen für Kinder und Jugendliche zur Erfassung der gesundheitsbezogenen Lebensqualität, German parent versions of the KINDL-R Quality of Life Questionnaire for Children
M: Mean
MAR: Missing at random
MCAR: Missing completely at random
n: Number of participants
NoBaghira: Children who did not participate in the Baghira training despite a recommendation including children with a recommendation for both trainings and a higher or equal SDQ conduct problems than emotional problems score
Normal: Children evaluated as normal with no recommendation for prevention participation
NoTiger: Children who did not participate in the Tiger training despite a recommendation including children with a recommendation for both trainings and a higher SDQ emotional problems than conduct problems score
NoTraining: Children who did not participate in an indicated prevention program despite recommendation
p: Probability value
PROMPt: Primärindikative und optimierte Zuweisung zu gezielten Maßnahmen bei emotionalen und Verhaltensauffälligkeiten bei Kindern (Primary Indicative and Optimized Referral to Targeted Interventions for Emotional and Behavioral Problems in Children)
SD: Standard deviation
SDQ: Strengths and Difficulties Questionnaire
SE: Standard error
Tiger: Children who participated in the tiger training
Tiger training: Indicated group program for shy and anxious children “Mutig werden mit Til Tiger” (Becoming brave with Til Tiger)
Training: Children who participated in an indicated prevention program after recommendation
TUD: TUD Dresden University of Technology
α: Cronbachs Alpha, level of internal consistency
β: Standardized regression coefficient
