# Supplementary material for: Disruptive behavior and emotional problems in children screened in routine health care: prevalence and effectiveness of indicated prevention
Source: Child Adolesc Psychiatry Ment Health. 2025 Aug 14;19:93. doi: 10.1186/s13034-025-00949-7 (PMC12351797; doi:10.1186/s13034-025-00949-7)
Supplement: Supplementary file 1 — Supplementary Material 1 [file 13034_2025_949_MOESM1_ESM.docx]

**Supplementary Material**

Table S1. Overview of available data in the total sample by assigned group.

|  |  | **Normal** | |  | **Training** | |  | **NoTraining** | |  | **Abnormal** | |
| --- | --- | --- | --- | --- | --- | --- | --- | --- | --- | --- | --- | --- |
|  |  | n = 1932 | |  | n = 337 | |  | n = 597 | |  | n = 85 | |
|  |  | **n** | **%** |  | **n** | **%** |  | **n** | **%** |  | **n** | **%** |
| **SDQ emotional problems score available** |  |  |  |  |  |  |  |  |  |  |  |  |
| Screening |  | 1924 | 99.6 |  | 327 | 97.0 |  | 594 | 99.5 |  | 85 | 100.0 |
| T1 |  | 662 | 34.3 |  | 312 | 92.6 |  | 77 | 12.9 |  | 35 | 41.2 |
| T2 |  | 622 | 32.2 |  | 249 | 73.9 |  | 80 | 13.4 |  | 35 | 41.2 |
| Screening & T1 |  | 660 | 34.2 |  | 302 | 89.6 |  | 76 | 12.7 |  | 35 | 41.2 |
| Screening & T2 |  | 618 | 32.0 |  | 243 | 72.1 |  | 79 | 13.2 |  | 35 | 41.2 |
| T1 & T2 |  | 504 | 26.1 |  | 240 | 71.2 |  | 48 | 8.0 |  | 26 | 30.6 |
| at least one measurement time point |  | 1928 | 99.8 |  | 337 | 100.0 |  | 595 | 99.7 |  | 85 | 100.0 |
| at least two measurement time points |  | 778 | 40.3 |  | 317 | 94.1 |  | 109 | 18.3 |  | 44 | 51.8 |
| all three measurement time points |  | 502 | 26.0 |  | 234 | 69.4 |  | 47 | 7.9 |  | 26 | 30.6 |
| **SDQ conduct problems score available** |  |  |  |  |  |  |  |  |  |  |  |  |
| Screening |  | 1924 | 99.6 |  | 327 | 97.0 |  | 594 | 99.5 |  | 85 | 100.0 |
| T1 |  | 662 | 34.3 |  | 312 | 92.6 |  | 77 | 12.9 |  | 35 | 41.2 |
| T2 |  | 623 | 32.2 |  | 249 | 73.9 |  | 80 | 13.4 |  | 35 | 41.2 |
| Screening & T1 |  | 660 | 34.2 |  | 302 | 89.6 |  | 76 | 12.7 |  | 35 | 41.2 |
| Screening & T2 |  | 619 | 32.0 |  | 243 | 72.1 |  | 79 | 13.2 |  | 35 | 41.2 |
| T1 & T2 |  | 505 | 26.1 |  | 240 | 71.2 |  | 48 | 8.0 |  | 26 | 30.6 |
| at least one measurement time point |  | 1928 | 99.8 |  | 337 | 100.0 |  | 595 | 99.7 |  | 85 | 100.0 |
| at least two measurement time points |  | 778 | 40.3 |  | 317 | 94.1 |  | 109 | 18.3 |  | 44 | 51.8 |
| all three measurement time points |  | 503 | 26.0 |  | 234 | 69.4 |  | 47 | 7.9 |  | 26 | 30.6 |
| **KINDL physical well-being score available** |  |  |  |  |  |  |  |  |  |  |  |  |
| T0 |  | 752 | 38.9 |  | 328 | 97.3 |  | 95 | 15.9 |  | 48 | 56.5 |
| T1 |  | 658 | 34.1 |  | 311 | 92.3 |  | 77 | 12.9 |  | 35 | 41.2 |
| T2 |  | 620 | 32.1 |  | 246 | 73.0 |  | 79 | 13.2 |  | 35 | 41.2 |
| T0 & T1 |  | 556 | 28.8 |  | 305 | 90.5 |  | 47 | 7.9 |  | 31 | 36.5 |
| T0 & T2 |  | 515 | 26.7 |  | 242 | 71.8 |  | 43 | 7.2 |  | 31 | 36.5 |
| T1 & T2 |  | 503 | 26.0 |  | 236 | 70.0 |  | 47 | 7.9 |  | 26 | 30.6 |
| at least one measurement time point |  | 911 | 47.2 |  | 334 | 99.1 |  | 148 | 24.8 |  | 54 | 63.5 |
| at least two measurement time points |  | 664 | 34.4 |  | 319 | 94.7 |  | 69 | 11.6 |  | 40 | 47.1 |
| all three measurement time points |  | 455 | 23.6 |  | 232 | 68.8 |  | 34 | 5.7 |  | 24 | 28.2 |
| **KINDL emotional well-being score available** |  |  |  |  |  |  |  |  |  |  |  |  |
| T0 |  | 751 | 38.9 |  | 327 | 97.0 |  | 95 | 15.9 |  | 48 | 56.5 |
| T1 |  | 658 | 34.1 |  | 311 | 92.3 |  | 77 | 12.9 |  | 35 | 41.2 |
| T2 |  | 620 | 32.1 |  | 246 | 73.0 |  | 79 | 13.2 |  | 35 | 41.2 |
| T0 & T1 |  | 556 | 28.8 |  | 304 | 90.2 |  | 47 | 7.9 |  | 31 | 36.5 |
| T0 & T2 |  | 515 | 26.7 |  | 242 | 71.8 |  | 43 | 7.2 |  | 31 | 36.5 |
| T1 & T2 |  | 503 | 26.0 |  | 236 | 70.0 |  | 47 | 7.9 |  | 26 | 30.6 |
| at least one measurement time point |  | 910 | 47.1 |  | 334 | 99.1 |  | 148 | 24.8 |  | 54 | 63.5 |
| at least two measurement time point |  | 664 | 34.4 |  | 318 | 94.4 |  | 69 | 11.6 |  | 40 | 47.1 |
| all three measurement time points |  | 455 | 23.6 |  | 232 | 68.8 |  | 34 | 5.7 |  | 24 | 28.2 |
| **KINDL self-esteem score available** |  |  |  |  |  |  |  |  |  |  |  |  |
| T0 |  | 751 | 38.9 |  | 327 | 97.0 |  | 95 | 15.9 |  | 48 | 56.5 |
| T1 |  | 658 | 34.1 |  | 311 | 92.3 |  | 77 | 12.9 |  | 35 | 41.2 |
| T2 |  | 620 | 32.1 |  | 246 | 73.0 |  | 79 | 13.2 |  | 35 | 41.2 |
| T0 & T1 |  | 556 | 28.8 |  | 304 | 90.2 |  | 47 | 7.9 |  | 31 | 36.5 |
| T0 & T2 |  | 515 | 26.7 |  | 242 | 71.8 |  | 43 | 7.2 |  | 31 | 36.5 |
| T1 & T2 |  | 503 | 26.0 |  | 236 | 70.0 |  | 47 | 7.9 |  | 26 | 30.6 |
| at least one measurement time point |  | 910 | 47.1 |  | 334 | 99.1 |  | 148 | 24.8 |  | 54 | 63.5 |
| at least two measurement time point |  | 664 | 34.4 |  | 318 | 94.4 |  | 69 | 11.6 |  | 40 | 47.1 |
| all three measurement time points |  | 455 | 23.6 |  | 232 | 68.8 |  | 34 | 5.7 |  | 24 | 28.2 |
| **KINDL familiy score available** |  |  |  |  |  |  |  |  |  |  |  |  |
| T0 |  | 751 | 38.9 |  | 327 | 97.0 |  | 95 | 15.9 |  | 48 | 56.5 |
| T1 |  | 658 | 34.1 |  | 311 | 92.3 |  | 77 | 12.9 |  | 35 | 41.2 |
| T2 |  | 620 | 32.1 |  | 246 | 73.0 |  | 79 | 13.2 |  | 35 | 41.2 |
| T0 & T1 |  | 556 | 28.8 |  | 304 | 90.2 |  | 47 | 7.9 |  | 31 | 36.5 |
| T0 & T2 |  | 515 | 26.7 |  | 242 | 71.8 |  | 43 | 7.2 |  | 31 | 36.5 |
| T1 & T2 |  | 503 | 26.0 |  | 236 | 70.0 |  | 47 | 7.9 |  | 26 | 30.6 |
| at least one measurement time point |  | 910 | 47.1 |  | 334 | 99.1 |  | 148 | 24.8 |  | 54 | 63.5 |
| at least two measurement time point |  | 664 | 34.4 |  | 318 | 94.4 |  | 69 | 11.6 |  | 40 | 47.1 |
| all three measurement time points |  | 455 | 23.6 |  | 232 | 68.8 |  | 34 | 5.7 |  | 24 | 28.2 |
| **KINDL friends score available** |  |  |  |  |  |  |  |  |  |  |  |  |
| T0 |  | 750 | 38.8 |  | 327 | 97.0 |  | 95 | 15.9 |  | 48 | 56.5 |
| T1 |  | 658 | 34.1 |  | 311 | 92.3 |  | 77 | 12.9 |  | 35 | 41.2 |
| T2 |  | 620 | 32.1 |  | 246 | 73.0 |  | 79 | 13.2 |  | 35 | 41.2 |
| T0 & T1 |  | 556 | 28.8 |  | 304 | 90.2 |  | 47 | 7.9 |  | 31 | 36.5 |
| T0 & T2 |  | 515 | 26.7 |  | 242 | 71.8 |  | 43 | 7.2 |  | 31 | 36.5 |
| T1 & T2 |  | 503 | 26.0 |  | 236 | 70.0 |  | 47 | 7.9 |  | 26 | 30.6 |
| at least one measurement time point |  | 909 | 47.0 |  | 334 | 99.1 |  | 148 | 24.8 |  | 54 | 63.5 |
| at least two measurement time point |  | 664 | 34.4 |  | 318 | 94.4 |  | 69 | 11.6 |  | 40 | 47.1 |
| all three measurement time points |  | 455 | 23.6 |  | 232 | 68.8 |  | 34 | 5.7 |  | 24 | 28.2 |
| **KINDL everyday functioning score available** |  |  |  |  |  |  |  |  |  |  |  |  |
| T0 |  | 744 | 38.5 |  | 325 | 96.4 |  | 94 | 15.7 |  | 48 | 56.5 |
| T1 |  | 655 | 33.9 |  | 310 | 92.0 |  | 75 | 12.6 |  | 35 | 41.2 |
| T2 |  | 615 | 31.8 |  | 245 | 72.7 |  | 78 | 13.1 |  | 34 | 40.0 |
| T0 & T1 |  | 550 | 28.5 |  | 301 | 89.3 |  | 46 | 7.7 |  | 31 | 36.5 |
| T0 & T2 |  | 507 | 26.2 |  | 239 | 70.9 |  | 42 | 7.0 |  | 30 | 35.3 |
| T1 & T2 |  | 497 | 25.7 |  | 234 | 69.4 |  | 45 | 7.5 |  | 25 | 29.4 |
| at least one measurement time point |  | 907 | 46.9 |  | 334 | 99.1 |  | 146 | 24.5 |  | 54 | 63.5 |
| at least two measurement time point |  | 660 | 34.2 |  | 318 | 94.4 |  | 69 | 11.6 |  | 40 | 47.1 |
| all three measurement time points |  | 447 | 23.1 |  | 228 | 67.7 |  | 32 | 5.4 |  | 23 | 27.1 |

*Note.* Due to high dropout rates, especially between screening and T0, separate analysis samples were build for SDQ and KINDL analyses, with only subjects included who had data of the respective sub-scales available at least at one measurement time point. SDQ = Strengths and Difficulties Questionnaire, KINDL = Kiddy-KINDL-R and Kid-/Kiddo-KINDL-R Quality of Life Questionnaire for Children, Normal = children evaluated as normal with no recommendation for indicated prevention participation; Training = children who participated in an indicated prevention program after recommendation, NoTraining = children who did not participate in an indicated prevention program despite recommendation; Abnormal = children with abnormal or clinically significant disruptive behavior or emotional problems or that did not fullfill inclusion criteria for participation in an indicated prevention program; n = sample size/number of participants; M = mean; SD = standard deviation; df = degrees of freedom; p = p-value

Table S2. Overview of available data in the total sample by training group.

|  |  | **Baghira** | |  | **Tiger** | |  | **NoBaghira** | |  | **NoTiger** | |
| --- | --- | --- | --- | --- | --- | --- | --- | --- | --- | --- | --- | --- |
|  |  | n = 192 | |  | n = 145 | |  | n = 351 | |  | n = 246 | |
|  |  | **n** | **%** |  | **n** | **%** |  | **n** | **%** |  | **n** | **%** |
| **SDQ emotional problems score available** |  |  |  |  |  |  |  |  |  |  |  |  |
| Screening |  | 187 | 97.4 |  | 140 | 96.6 |  | 350 | 99.7 |  | 244 | 99.2 |
| T1 |  | 178 | 92.7 |  | 134 | 92.4 |  | 42 | 12.0 |  | 35 | 14.2 |
| T2 |  | 140 | 72.9 |  | 109 | 75.2 |  | 50 | 14.2 |  | 30 | 12.2 |
| Screening & T1 |  | 173 | 90.1 |  | 129 | 89.0 |  | 42 | 12.0 |  | 34 | 13.8 |
| Screening & T2 |  | 137 | 71.4 |  | 106 | 73.1 |  | 50 | 14.2 |  | 29 | 11.8 |
| T1 & T2 |  | 136 | 70.8 |  | 104 | 71.7 |  | 28 | 8.0 |  | 20 | 8.1 |
| at least one measurement time point |  | 192 | 100.0 |  | 145 | 100.0 |  | 350 | 99.7 |  | 245 | 99.6 |
| at least two measurement time points |  | 180 | 93.8 |  | 137 | 94.5 |  | 64 | 18.2 |  | 45 | 18.3 |
| all three measurement time points |  | 133 | 69.3 |  | 101 | 69.7 |  | 28 | 8.0 |  | 19 | 7.7 |
| **SDQ conduct problems score available** |  |  |  |  |  |  |  |  |  |  |  |  |
| Screening |  | 187 | 97.4 |  | 140 | 96.6 |  | 350 | 99.7 |  | 244 | 99.2 |
| T1 |  | 178 | 92.7 |  | 134 | 92.4 |  | 42 | 12.0 |  | 35 | 14.2 |
| T2 |  | 140 | 72.9 |  | 109 | 75.2 |  | 50 | 14.2 |  | 30 | 12.2 |
| Screening & T1 |  | 173 | 90.1 |  | 129 | 89.0 |  | 42 | 12.0 |  | 34 | 13.8 |
| Screening & T2 |  | 137 | 71.4 |  | 106 | 73.1 |  | 50 | 14.2 |  | 29 | 11.8 |
| T1 & T2 |  | 136 | 70.8 |  | 104 | 71.7 |  | 28 | 8.0 |  | 20 | 8.1 |
| at least one measurement time point |  | 192 | 100.0 |  | 145 | 100.0 |  | 350 | 99.7 |  | 245 | 99.6 |
| at least two measurement time points |  | 180 | 93.8 |  | 137 | 94.5 |  | 64 | 18.2 |  | 45 | 18.3 |
| all three measurement time points |  | 133 | 69.3 |  | 101 | 69.7 |  | 28 | 8.0 |  | 19 | 7.7 |
| **KINDL physical well-being score available** |  |  |  |  |  |  |  |  |  |  |  |  |
| T0 |  | 186 | 96.9 |  | 142 | 97.9 |  | 52 | 14.8 |  | 43 | 17.5 |
| T1 |  | 177 | 92.2 |  | 134 | 92.4 |  | 42 | 12.0 |  | 35 | 14.2 |
| T2 |  | 139 | 72.4 |  | 107 | 73.8 |  | 50 | 14.2 |  | 29 | 11.8 |
| T0 & T1 |  | 172 | 89.6 |  | 133 | 91.7 |  | 25 | 7.1 |  | 22 | 8.9 |
| T0 & T2 |  | 135 | 70.3 |  | 107 | 73.8 |  | 24 | 6.8 |  | 19 | 7.7 |
| T1 & T2 |  | 134 | 69.8 |  | 102 | 70.3 |  | 28 | 8.0 |  | 19 | 7.7 |
| at least one measurement time point |  | 191 | 99.5 |  | 143 | 98.6 |  | 85 | 24.2 |  | 63 | 25.6 |
| at least two measurement time points |  | 181 | 94.3 |  | 138 | 95.2 |  | 41 | 11.7 |  | 28 | 11.4 |
| all three measurement time points |  | 130 | 67.7 |  | 102 | 70.3 |  | 18 | 5.1 |  | 16 | 6.5 |
| **KINDL emotional well-being score available** |  |  |  |  |  |  |  |  |  |  |  |  |
| T0 |  | 186 | 96.9 |  | 141 | 97.2 |  | 52 | 14.8 |  | 43 | 17.5 |
| T1 |  | 177 | 92.2 |  | 134 | 92.4 |  | 42 | 12.0 |  | 35 | 14.2 |
| T2 |  | 139 | 72.4 |  | 107 | 73.8 |  | 50 | 14.2 |  | 29 | 11.8 |
| T0 & T1 |  | 172 | 89.6 |  | 132 | 91.0 |  | 25 | 7.1 |  | 22 | 8.9 |
| T0 & T2 |  | 135 | 70.3 |  | 107 | 73.8 |  | 24 | 6.8 |  | 19 | 7.7 |
| T1 & T2 |  | 134 | 69.8 |  | 102 | 70.3 |  | 28 | 8.0 |  | 19 | 7.7 |
| at least one measurement time point |  | 191 | 99.5 |  | 143 | 98.6 |  | 85 | 24.2 |  | 63 | 25.6 |
| at least two measurement time point |  | 181 | 94.3 |  | 137 | 94.5 |  | 41 | 11.7 |  | 28 | 11.4 |
| all three measurement time points |  | 130 | 67.7 |  | 102 | 70.3 |  | 18 | 5.1 |  | 16 | 6.5 |
| **KINDL self-esteem score available** |  |  |  |  |  |  |  |  |  |  |  |  |
| T0 |  | 186 | 96.9 |  | 141 | 97.2 |  | 52 | 14.8 |  | 43 | 17.5 |
| T1 |  | 177 | 92.2 |  | 134 | 92.4 |  | 42 | 12.0 |  | 35 | 14.2 |
| T2 |  | 139 | 72.4 |  | 107 | 73.8 |  | 50 | 14.2 |  | 29 | 11.8 |
| T0 & T1 |  | 172 | 89.6 |  | 132 | 91.0 |  | 25 | 7.1 |  | 22 | 8.9 |
| T0 & T2 |  | 135 | 70.3 |  | 107 | 73.8 |  | 24 | 6.8 |  | 19 | 7.7 |
| T1 & T2 |  | 134 | 69.8 |  | 102 | 70.3 |  | 28 | 8.0 |  | 19 | 7.7 |
| at least one measurement time point |  | 191 | 99.5 |  | 143 | 98.6 |  | 85 | 24.2 |  | 63 | 25.6 |
| at least two measurement time point |  | 181 | 94.3 |  | 137 | 94.5 |  | 41 | 11.7 |  | 28 | 11.4 |
| all three measurement time points |  | 130 | 67.7 |  | 102 | 70.3 |  | 18 | 5.1 |  | 16 | 6.5 |
| **KINDL familiy score available** |  |  |  |  |  |  |  |  |  |  |  |  |
| T0 |  | 186 | 96.9 |  | 141 | 97.2 |  | 52 | 14.8 |  | 43 | 17.5 |
| T1 |  | 177 | 92.2 |  | 134 | 92.4 |  | 42 | 12.0 |  | 35 | 14.2 |
| T2 |  | 139 | 72.4 |  | 107 | 73.8 |  | 50 | 14.2 |  | 29 | 11.8 |
| T0 & T1 |  | 172 | 89.6 |  | 132 | 91.0 |  | 25 | 7.1 |  | 22 | 8.9 |
| T0 & T2 |  | 135 | 70.3 |  | 107 | 73.8 |  | 24 | 6.8 |  | 19 | 7.7 |
| T1 & T2 |  | 134 | 69.8 |  | 102 | 70.3 |  | 28 | 8.0 |  | 19 | 7.7 |
| at least one measurement time point |  | 191 | 99.5 |  | 143 | 98.6 |  | 85 | 24.2 |  | 63 | 25.6 |
| at least two measurement time point |  | 181 | 94.3 |  | 137 | 94.5 |  | 41 | 11.7 |  | 28 | 11.4 |
| all three measurement time points |  | 130 | 67.7 |  | 102 | 70.3 |  | 18 | 5.1 |  | 16 | 6.5 |
| **KINDL friends score available** |  |  |  |  |  |  |  |  |  |  |  |  |
| T0 |  | 186 | 96.9 |  | 141 | 97.2 |  | 52 | 14.8 |  | 43 | 17.5 |
| T1 |  | 177 | 92.2 |  | 134 | 92.4 |  | 42 | 12.0 |  | 35 | 14.2 |
| T2 |  | 139 | 72.4 |  | 107 | 73.8 |  | 50 | 14.2 |  | 29 | 11.8 |
| T0 & T1 |  | 172 | 89.6 |  | 132 | 91.0 |  | 25 | 7.1 |  | 22 | 8.9 |
| T0 & T2 |  | 135 | 70.3 |  | 107 | 73.8 |  | 24 | 6.8 |  | 19 | 7.7 |
| T1 & T2 |  | 134 | 69.8 |  | 102 | 70.3 |  | 28 | 8.0 |  | 19 | 7.7 |
| at least one measurement time point |  | 191 | 99.5 |  | 143 | 98.6 |  | 85 | 24.2 |  | 63 | 25.6 |
| at least two measurement time point |  | 181 | 94.3 |  | 137 | 94.5 |  | 41 | 11.7 |  | 28 | 11.4 |
| all three measurement time points |  | 130 | 67.7 |  | 102 | 70.3 |  | 18 | 5.1 |  | 16 | 6.5 |
| **KINDL everyday functioning score available** |  |  |  |  |  |  |  |  |  |  |  |  |
| T0 |  | 185 | 96.4 |  | 140 | 96.6 |  | 51 | 14.5 |  | 43 | 17.5 |
| T1 |  | 176 | 91.7 |  | 134 | 92.4 |  | 40 | 11.4 |  | 35 | 14.2 |
| T2 |  | 138 | 71.9 |  | 107 | 73.8 |  | 49 | 14.0 |  | 29 | 11.8 |
| T0 & T1 |  | 170 | 88.5 |  | 131 | 90.3 |  | 24 | 6.8 |  | 22 | 8.9 |
| T0 & T2 |  | 133 | 69.3 |  | 106 | 73.1 |  | 23 | 6.6 |  | 19 | 7.7 |
| T1 & T2 |  | 132 | 68.8 |  | 102 | 70.3 |  | 26 | 7.4 |  | 19 | 7.7 |
| at least one measurement time point |  | 191 | 99.5 |  | 143 | 98.6 |  | 83 | 23.6 |  | 63 | 25.6 |
| at least two measurement time point |  | 181 | 94.3 |  | 137 | 94.5 |  | 41 | 11.7 |  | 28 | 11.4 |
| all three measurement time points |  | 127 | 66.1 |  | 101 | 69.7 |  | 16 | 4.6 |  | 16 | 6.5 |

*Note.* Due to high dropout rates, especially between screening and T0, separate analysis samples were build for SDQ and KINDL analyses, with only subjects included who had data of the respective sub-scales available at least at one measurement time point. SDQ = Strengths and Difficulties Questionnaire, KINDL = Kiddy-KINDL-R and Kid-/Kiddo-KINDL-R Quality of Life Questionnaire for Children, Baghira = children who participated in the Baghira training; Tiger = children who participated in the Tiger training; NoBaghira = children who did not participate in the Baghira training despite a recommendation including children with a recommendation for both trainings and a higher or equal SDQ conduct problems than emotional problems score; NoTiger = children who did not participate in the Tiger training despite a recommendation including children with a recommendation for both trainings and a higher SDQ emotional problems than conduct problems score; n = sample size/number of participants; M = mean; SD = standard deviation; df = degrees of freedom; p = p-value

Table S3. Overview of available data in the analysis samples by assigned group.

|  |  | **Normal** | |  | **Training** | |  | **NoTraining** | |  | **Abnormal** | |
| --- | --- | --- | --- | --- | --- | --- | --- | --- | --- | --- | --- | --- |
|  |  | **n** | **%** |  | **n** | **%** |  | **n** | **%** |  | **n** | **%** |
| **Total SDQ analysis sample** |  | 1928 |  |  | 337 |  |  | 595 |  |  | 85 |  |
| **SDQ emotional problems score available** |  |  |  |  |  |  |  |  |  |  |  |  |
| Screening |  | 1924 | 99.8 |  | 327 | 97.0 |  | 594 | 99.8 |  | 85 | 100.0 |
| T1 |  | 662 | 34.3 |  | 312 | 92.6 |  | 77 | 12.9 |  | 35 | 41.2 |
| T2 |  | 622 | 32.3 |  | 249 | 73.9 |  | 80 | 13.4 |  | 35 | 41.2 |
| Screening & T1 |  | 660 | 34.2 |  | 302 | 89.6 |  | 76 | 12.8 |  | 35 | 41.2 |
| Screening & T2 |  | 618 | 32.1 |  | 243 | 72.1 |  | 79 | 13.3 |  | 35 | 41.2 |
| T1 & T2 |  | 504 | 26.1 |  | 240 | 71.2 |  | 48 | 8.1 |  | 26 | 30.6 |
| at least one measurement time point |  | 1928 | 100.0 |  | 337 | 100.0 |  | 595 | 100.0 |  | 85 | 100.0 |
| at least two measurement time points |  | 778 | 40.4 |  | 317 | 94.1 |  | 109 | 18.3 |  | 44 | 51.8 |
| all three measurement time points |  | 502 | 26.0 |  | 234 | 69.4 |  | 47 | 7.9 |  | 26 | 30.6 |
| **SDQ conduct problems score available** |  |  |  |  |  |  |  |  |  |  |  |  |
| Screening |  | 1924 | 99.8 |  | 327 | 97.0 |  | 594 | 99.8 |  | 85 | 100.0 |
| T1 |  | 662 | 34.3 |  | 312 | 92.6 |  | 77 | 12.9 |  | 35 | 41.2 |
| T2 |  | 623 | 32.3 |  | 249 | 73.9 |  | 80 | 13.4 |  | 35 | 41.2 |
| Screening & T1 |  | 660 | 34.2 |  | 302 | 89.6 |  | 76 | 12.8 |  | 35 | 41.2 |
| Screening & T2 |  | 619 | 32.1 |  | 243 | 72.1 |  | 79 | 13.3 |  | 35 | 41.2 |
| T1 & T2 |  | 505 | 26.2 |  | 240 | 71.2 |  | 48 | 8.1 |  | 26 | 30.6 |
| at least one measurement time point |  | 1928 | 100.0 |  | 337 | 100.0 |  | 595 | 100.0 |  | 85 | 100.0 |
| at least two measurement time points |  | 778 | 40.4 |  | 317 | 94.1 |  | 109 | 18.3 |  | 44 | 51.8 |
| all three measurement time points |  | 503 | 26.1 |  | 234 | 69.4 |  | 47 | 7.9 |  | 26 | 30.6 |
| **Total KINDL analysis sample** |  | 907 |  |  | 334 |  |  | 146 |  |  | 54 |  |
| **KINDL physical well-being score available** |  |  |  |  |  |  |  |  |  |  |  |  |
| T0 |  | 748 | 82.5 |  | 328 | 98.2 |  | 94 | 64.4 |  | 48 | 88.9 |
| T1 |  | 658 | 72.5 |  | 311 | 93.1 |  | 76 | 52.1 |  | 35 | 64.8 |
| T2 |  | 620 | 68.4 |  | 246 | 73.7 |  | 79 | 54.1 |  | 35 | 64.8 |
| T0 & T1 |  | 556 | 61.3 |  | 305 | 91.3 |  | 47 | 32.2 |  | 31 | 57.4 |
| T0 & T2 |  | 515 | 56.8 |  | 242 | 72.5 |  | 43 | 29.5 |  | 31 | 57.4 |
| T1 & T2 |  | 503 | 55.5 |  | 236 | 70.7 |  | 47 | 32.2 |  | 26 | 48.1 |
| at least one measurement time point |  | 907 | 100.0 |  | 334 | 100.0 |  | 146 | 100.0 |  | 54 | 100.0 |
| at least two measurement time points |  | 664 | 73.2 |  | 319 | 95.5 |  | 69 | 47.3 |  | 40 | 74.1 |
| all three measurement time points |  | 455 | 50.2 |  | 232 | 69.5 |  | 34 | 23.3 |  | 24 | 44.4 |
| **KINDL emotional well-being score available** |  |  |  |  |  |  |  |  |  |  |  |  |
| T0 |  | 748 | 82.5 |  | 327 | 97.9 |  | 94 | 64.4 |  | 48 | 88.9 |
| T1 |  | 658 | 72.5 |  | 311 | 93.1 |  | 76 | 52.1 |  | 35 | 64.8 |
| T2 |  | 620 | 68.4 |  | 246 | 73.7 |  | 79 | 54.1 |  | 35 | 64.8 |
| T0 & T1 |  | 556 | 61.3 |  | 304 | 91.0 |  | 47 | 32.2 |  | 31 | 57.4 |
| T0 & T2 |  | 515 | 56.8 |  | 242 | 72.5 |  | 43 | 29.5 |  | 31 | 57.4 |
| T1 & T2 |  | 503 | 55.5 |  | 236 | 70.7 |  | 47 | 32.2 |  | 26 | 48.1 |
| at least one measurement time point |  | 907 | 100.0 |  | 334 | 100.0 |  | 146 | 100.0 |  | 54 | 100.0 |
| at least two measurement time points |  | 664 | 73.2 |  | 318 | 95.2 |  | 69 | 47.3 |  | 40 | 74.1 |
| all three measurement time points |  | 455 | 50.2 |  | 232 | 69.5 |  | 34 | 23.3 |  | 24 | 44.4 |
| **KINDL self-esteem score available** |  |  |  |  |  |  |  |  |  |  |  |  |
| T0 |  | 748 | 82.5 |  | 327 | 97.9 |  | 94 | 64.4 |  | 48 | 88.9 |
| T1 |  | 658 | 72.5 |  | 311 | 93.1 |  | 76 | 52.1 |  | 35 | 64.8 |
| T2 |  | 620 | 68.4 |  | 246 | 73.7 |  | 79 | 54.1 |  | 35 | 64.8 |
| T0 & T1 |  | 556 | 61.3 |  | 304 | 91.0 |  | 47 | 32.2 |  | 31 | 57.4 |
| T0 & T2 |  | 515 | 56.8 |  | 242 | 72.5 |  | 43 | 29.5 |  | 31 | 57.4 |
| T1 & T2 |  | 503 | 55.5 |  | 236 | 70.7 |  | 47 | 32.2 |  | 26 | 48.1 |
| at least one measurement time point |  | 907 | 100.0 |  | 334 | 100.0 |  | 146 | 100.0 |  | 54 | 100.0 |
| at least two measurement time point |  | 664 | 73.2 |  | 318 | 95.2 |  | 69 | 47.3 |  | 40 | 74.1 |
| all three measurement time points |  | 455 | 50.2 |  | 232 | 69.5 |  | 34 | 23.3 |  | 24 | 44.4 |
| **KINDL familiy score available** |  |  |  |  |  |  |  |  |  |  |  |  |
| T0 |  | 748 | 82.5 |  | 327 | 97.9 |  | 94 | 64.4 |  | 48 | 88.9 |
| T1 |  | 658 | 72.5 |  | 311 | 93.1 |  | 76 | 52.1 |  | 35 | 64.8 |
| T2 |  | 620 | 68.4 |  | 246 | 73.7 |  | 79 | 54.1 |  | 35 | 64.8 |
| T0 & T1 |  | 556 | 61.3 |  | 304 | 91.0 |  | 47 | 32.2 |  | 31 | 57.4 |
| T0 & T2 |  | 515 | 56.8 |  | 242 | 72.5 |  | 43 | 29.5 |  | 31 | 57.4 |
| T1 & T2 |  | 503 | 55.5 |  | 236 | 70.7 |  | 47 | 32.2 |  | 26 | 48.1 |
| at least one measurement time point |  | 907 | 100.0 |  | 334 | 100.0 |  | 146 | 100.0 |  | 54 | 100.0 |
| at least two measurement time point |  | 664 | 73.2 |  | 318 | 95.2 |  | 69 | 47.3 |  | 40 | 74.1 |
| all three measurement time points |  | 455 | 50.2 |  | 232 | 69.5 |  | 34 | 23.3 |  | 24 | 44.4 |
| **KINDL friends score available** |  |  |  |  |  |  |  |  |  |  |  |  |
| T0 |  | 748 | 82.5 |  | 327 | 97.9 |  | 94 | 64.4 |  | 48 | 88.9 |
| T1 |  | 658 | 72.5 |  | 311 | 93.1 |  | 76 | 52.1 |  | 35 | 64.8 |
| T2 |  | 620 | 68.4 |  | 246 | 73.7 |  | 79 | 54.1 |  | 35 | 64.8 |
| T0 & T1 |  | 556 | 61.3 |  | 304 | 91.0 |  | 47 | 32.2 |  | 31 | 57.4 |
| T0 & T2 |  | 515 | 56.8 |  | 242 | 72.5 |  | 43 | 29.5 |  | 31 | 57.4 |
| T1 & T2 |  | 503 | 55.5 |  | 236 | 70.7 |  | 47 | 32.2 |  | 26 | 48.1 |
| at least one measurement time point |  | 907 | 100.0 |  | 334 | 100.0 |  | 146 | 100.0 |  | 54 | 100.0 |
| at least two measurement time point |  | 664 | 73.2 |  | 318 | 95.2 |  | 69 | 47.3 |  | 40 | 74.1 |
| all three measurement time points |  | 455 | 50.2 |  | 232 | 69.5 |  | 34 | 23.3 |  | 24 | 44.4 |
| **KINDL everyday functioning score available** |  |  |  |  |  |  |  |  |  |  |  |  |
| T0 |  | 744 | 82.0 |  | 325 | 97.3 |  | 94 | 64.4 |  | 48 | 88.9 |
| T1 |  | 655 | 72.2 |  | 310 | 92.8 |  | 75 | 51.4 |  | 35 | 64.8 |
| T2 |  | 615 | 67.8 |  | 245 | 73.4 |  | 78 | 53.4 |  | 34 | 63.0 |
| T0 & T1 |  | 550 | 60.6 |  | 301 | 90.1 |  | 46 | 31.5 |  | 31 | 57.4 |
| T0 & T2 |  | 507 | 55.9 |  | 239 | 71.6 |  | 42 | 28.8 |  | 30 | 55.6 |
| T1 & T2 |  | 497 | 54.8 |  | 234 | 70.1 |  | 45 | 30.8 |  | 25 | 46.3 |
| at least one measurement time point |  | 907 | 100.0 |  | 334 | 100.0 |  | 146 | 100.0 |  | 54 | 100.0 |
| at least two measurement time point |  | 660 | 72.8 |  | 318 | 95.2 |  | 69 | 47.3 |  | 40 | 74.1 |
| all three measurement time points |  | 447 | 49.3 |  | 228 | 68.3 |  | 32 | 21.9 |  | 23 | 42.6 |

*Note.* Due to high dropout rates, especially between screening and T0, separate analysis samples were build for SDQ and KINDL analyses, with only subjects included who had data of the respective sub-scales available at least at one measurement time point. SDQ = Strengths and Difficulties Questionnaire, KINDL = Kiddy-KINDL-R and Kid-/Kiddo-KINDL-R Quality of Life Questionnaire for Children, Normal = children evaluated as normal with no recommendation for indicated prevention participation; Training = children who participated in an indicated prevention program after recommendation, NoTraining = children who did not participate in an indicated prevention program despite recommendation; Abnormal = children with abnormal or clinically significant disruptive behavior or emotional problems or that did not fullfill inclusion criteria for participation in an indicated prevention program; n = sample size/number of participants; M = mean; SD = standard deviation; df = degrees of freedom; p = p-value

Table S4. Overview of available data in the analysis samples by training group.

|  |  | **Baghira** | |  | **Tiger** | |  | **NoBaghira** | |  | **NoTiger** | |
| --- | --- | --- | --- | --- | --- | --- | --- | --- | --- | --- | --- | --- |
|  |  | **n** | **%** |  | **n** | **%** |  | **n** | **%** |  | **n** | **%** |
| **Total SDQ analysis sample** |  | 192 |  |  | 145 |  |  | 350 |  |  | 245 |  |
| **SDQ emotional problems score available** |  |  |  |  |  |  |  |  |  |  |  |  |
| Screening |  | 187 | 97.4 |  | 140 | 96.6 |  | 350 | 100.0 |  | 244 | 99.6 |
| T1 |  | 178 | 92.7 |  | 134 | 92.4 |  | 42 | 12.0 |  | 35 | 14.3 |
| T2 |  | 140 | 72.9 |  | 109 | 75.2 |  | 50 | 14.3 |  | 30 | 12.2 |
| Screening & T1 |  | 173 | 90.1 |  | 129 | 89.0 |  | 42 | 12.0 |  | 34 | 13.9 |
| Screening & T2 |  | 137 | 71.4 |  | 106 | 73.1 |  | 50 | 14.3 |  | 29 | 11.8 |
| T1 & T2 |  | 136 | 70.8 |  | 104 | 71.7 |  | 28 | 8.0 |  | 20 | 8.2 |
| at least one measurement time point |  | 192 | 100.0 |  | 145 | 100.0 |  | 350 | 100.0 |  | 245 | 100.0 |
| at least two measurement time points |  | 180 | 93.8 |  | 137 | 94.5 |  | 64 | 18.3 |  | 45 | 18.4 |
| all three measurement time points |  | 133 | 69.3 |  | 101 | 69.7 |  | 28 | 8.0 |  | 19 | 7.8 |
| **SDQ conduct problems score available** |  |  |  |  |  |  |  |  |  |  |  |  |
| Screening |  | 187 | 97.4 |  | 140 | 96.6 |  | 350 | 100.0 |  | 244 | 99.6 |
| T1 |  | 178 | 92.7 |  | 134 | 92.4 |  | 42 | 12.0 |  | 35 | 14.3 |
| T2 |  | 140 | 72.9 |  | 109 | 75.2 |  | 50 | 14.3 |  | 30 | 12.2 |
| Screening & T1 |  | 173 | 90.1 |  | 129 | 89.0 |  | 42 | 12.0 |  | 34 | 13.9 |
| Screening & T2 |  | 137 | 71.4 |  | 106 | 73.1 |  | 50 | 14.3 |  | 29 | 11.8 |
| T1 & T2 |  | 136 | 70.8 |  | 104 | 71.7 |  | 28 | 8.0 |  | 20 | 8.2 |
| at least one measurement time point |  | 192 | 100.0 |  | 145 | 100.0 |  | 350 | 100.0 |  | 245 | 100.0 |
| at least two measurement time points |  | 180 | 93.8 |  | 137 | 94.5 |  | 64 | 18.3 |  | 45 | 18.4 |
| all three measurement time points |  | 133 | 69.3 |  | 101 | 69.7 |  | 28 | 8.0 |  | 19 | 7.8 |
| **Total KINDL analysis sample** |  | 191 |  |  | 143 |  |  | 83 |  |  | 63 |  |
| **KINDL physical well-being score available** |  |  |  |  |  |  |  |  |  |  |  |  |
| T0 |  | 186 | 97.4 |  | 142 | 99.3 |  | 51 | 61.4 |  | 43 | 68.3 |
| T1 |  | 177 | 92.7 |  | 134 | 93.7 |  | 41 | 49.4 |  | 35 | 55.6 |
| T2 |  | 139 | 72.8 |  | 107 | 74.8 |  | 50 | 60.2 |  | 29 | 46.0 |
| T0 & T1 |  | 172 | 90.1 |  | 133 | 93.0 |  | 25 | 30.1 |  | 22 | 34.9 |
| T0 & T2 |  | 135 | 70.7 |  | 107 | 74.8 |  | 24 | 28.9 |  | 19 | 30.2 |
| T1 & T2 |  | 134 | 70.2 |  | 102 | 71.3 |  | 28 | 33.7 |  | 19 | 30.2 |
| at least one measurement time point |  | 191 | 100.0 |  | 143 | 100.0 |  | 83 | 100.0 |  | 63 | 100.0 |
| at least two measurement time points |  | 181 | 94.8 |  | 138 | 96.5 |  | 41 | 49.4 |  | 28 | 44.4 |
| all three measurement time points |  | 130 | 68.1 |  | 102 | 71.3 |  | 18 | 21.7 |  | 16 | 25.4 |
| **KINDL emotional well-being score available** |  |  |  |  |  |  |  |  |  |  |  |  |
| T0 |  | 186 | 97.4 |  | 141 | 98.6 |  | 51 | 61.4 |  | 43 | 68.3 |
| T1 |  | 177 | 92.7 |  | 134 | 93.7 |  | 41 | 49.4 |  | 35 | 55.6 |
| T2 |  | 139 | 72.8 |  | 107 | 74.8 |  | 50 | 60.2 |  | 29 | 46.0 |
| T0 & T1 |  | 172 | 90.1 |  | 132 | 92.3 |  | 25 | 30.1 |  | 22 | 34.9 |
| T0 & T2 |  | 135 | 70.7 |  | 107 | 74.8 |  | 24 | 28.9 |  | 19 | 30.2 |
| T1 & T2 |  | 134 | 70.2 |  | 102 | 71.3 |  | 28 | 33.7 |  | 19 | 30.2 |
| at least one measurement time point |  | 191 | 100.0 |  | 143 | 100.0 |  | 83 | 100.0 |  | 63 | 100.0 |
| at least two measurement time points |  | 181 | 94.8 |  | 137 | 95.8 |  | 41 | 49.4 |  | 28 | 44.4 |
| all three measurement time points |  | 130 | 68.1 |  | 102 | 71.3 |  | 18 | 21.7 |  | 16 | 25.4 |
| **KINDL self-esteem score available** |  |  |  |  |  |  |  |  |  |  |  |  |
| T0 |  | 186 | 97.4 |  | 141 | 98.6 |  | 51 | 61.4 |  | 43 | 68.3 |
| T1 |  | 177 | 92.7 |  | 134 | 93.7 |  | 41 | 49.4 |  | 35 | 55.6 |
| T2 |  | 139 | 72.8 |  | 107 | 74.8 |  | 50 | 60.2 |  | 29 | 46.0 |
| T0 & T1 |  | 172 | 90.1 |  | 132 | 92.3 |  | 25 | 30.1 |  | 22 | 34.9 |
| T0 & T2 |  | 135 | 70.7 |  | 107 | 74.8 |  | 24 | 28.9 |  | 19 | 30.2 |
| T1 & T2 |  | 134 | 70.2 |  | 102 | 71.3 |  | 28 | 33.7 |  | 19 | 30.2 |
| at least one measurement time point |  | 191 | 100.0 |  | 143 | 100.0 |  | 83 | 100.0 |  | 63 | 100.0 |
| at least two measurement time point |  | 181 | 94.8 |  | 137 | 95.8 |  | 41 | 49.4 |  | 28 | 44.4 |
| all three measurement time points |  | 130 | 68.1 |  | 102 | 71.3 |  | 18 | 21.7 |  | 16 | 25.4 |
| **KINDL familiy score available** |  |  |  |  |  |  |  |  |  |  |  |  |
| T0 |  | 186 | 97.4 |  | 141 | 98.6 |  | 51 | 61.4 |  | 43 | 68.3 |
| T1 |  | 177 | 92.7 |  | 134 | 93.7 |  | 41 | 49.4 |  | 35 | 55.6 |
| T2 |  | 139 | 72.8 |  | 107 | 74.8 |  | 50 | 60.2 |  | 29 | 46.0 |
| T0 & T1 |  | 172 | 90.1 |  | 132 | 92.3 |  | 25 | 30.1 |  | 22 | 34.9 |
| T0 & T2 |  | 135 | 70.7 |  | 107 | 74.8 |  | 24 | 28.9 |  | 19 | 30.2 |
| T1 & T2 |  | 134 | 70.2 |  | 102 | 71.3 |  | 28 | 33.7 |  | 19 | 30.2 |
| at least one measurement time point |  | 191 | 100.0 |  | 143 | 100.0 |  | 83 | 100.0 |  | 63 | 100.0 |
| at least two measurement time point |  | 181 | 94.8 |  | 137 | 95.8 |  | 41 | 49.4 |  | 28 | 44.4 |
| all three measurement time points |  | 130 | 68.1 |  | 102 | 71.3 |  | 18 | 21.7 |  | 16 | 25.4 |
| **KINDL friends score available** |  |  |  |  |  |  |  |  |  |  |  |  |
| T0 |  | 186 | 97.4 |  | 141 | 98.6 |  | 51 | 61.4 |  | 43 | 68.3 |
| T1 |  | 177 | 92.7 |  | 134 | 93.7 |  | 41 | 49.4 |  | 35 | 55.6 |
| T2 |  | 139 | 72.8 |  | 107 | 74.8 |  | 50 | 60.2 |  | 29 | 46.0 |
| T0 & T1 |  | 172 | 90.1 |  | 132 | 92.3 |  | 25 | 30.1 |  | 22 | 34.9 |
| T0 & T2 |  | 135 | 70.7 |  | 107 | 74.8 |  | 24 | 28.9 |  | 19 | 30.2 |
| T1 & T2 |  | 134 | 70.2 |  | 102 | 71.3 |  | 28 | 33.7 |  | 19 | 30.2 |
| at least one measurement time point |  | 191 | 100.0 |  | 143 | 100.0 |  | 83 | 100.0 |  | 63 | 100.0 |
| at least two measurement time point |  | 181 | 94.8 |  | 137 | 95.8 |  | 41 | 49.4 |  | 28 | 44.4 |
| all three measurement time points |  | 130 | 68.1 |  | 102 | 71.3 |  | 18 | 21.7 |  | 16 | 25.4 |
| **KINDL everyday functioning score available** |  |  |  |  |  |  |  |  |  |  |  |  |
| T0 |  | 185 | 96.9 |  | 140 | 97.9 |  | 51 | 61.4 |  | 43 | 68.3 |
| T1 |  | 176 | 92.1 |  | 134 | 93.7 |  | 40 | 48.2 |  | 35 | 55.6 |
| T2 |  | 138 | 72.3 |  | 107 | 74.8 |  | 49 | 59.0 |  | 29 | 46.0 |
| T0 & T1 |  | 170 | 89.0 |  | 131 | 91.6 |  | 24 | 28.9 |  | 22 | 34.9 |
| T0 & T2 |  | 133 | 69.6 |  | 106 | 74.1 |  | 23 | 27.7 |  | 19 | 30.2 |
| T1 & T2 |  | 132 | 69.1 |  | 102 | 71.3 |  | 26 | 31.3 |  | 19 | 30.2 |
| at least one measurement time point |  | 191 | 100.0 |  | 143 | 100.0 |  | 83 | 100.0 |  | 63 | 100.0 |
| at least two measurement time point |  | 181 | 94.8 |  | 137 | 95.8 |  | 41 | 49.4 |  | 28 | 44.4 |
| all three measurement time points |  | 127 | 66.5 |  | 101 | 70.6 |  | 16 | 19.3 |  | 16 | 25.4 |

*Note.* Due to high dropout rates, especially between screening and T0, separate analysis samples were build for SDQ and KINDL analyses, with only subjects included who had data of the respective sub-scales available at least at one measurement time point. SDQ = Strengths and Difficulties Questionnaire, KINDL = Kiddy-KINDL-R and Kid-/Kiddo-KINDL-R Quality of Life Questionnaire for Children, Baghira = children who participated in the Baghira training; Tiger = children who participated in the Tiger training; NoBaghira = children who did not participate in the Baghira training despite a recommendation including children with a recommendation for both trainings and a higher or equal SDQ conduct problems than emotional problems score; NoTiger = children who did not participate in the Tiger training despite a recommendation including children with a recommendation for both trainings and a higher SDQ emotional problems than conduct problems score; n = sample size/number of participants; M = mean; SD = standard deviation; df = degrees of freedom; p = p-value

Table S5. Comparison of children excluded from vs. included in the KINDL analysis sample

|  |  | **Excluded** | |  | **Included** | |  | **Comparison** | |
| --- | --- | --- | --- | --- | --- | --- | --- | --- | --- |
|  |  | n = 1510 | |  | n = 1441 | |  |  | |
|  |  | **n** | **% /**  **M (SD)** |  | **n** | **% /**  **M (SD)** |  | **t (df) /**  **Chi2 (df)** | **p** |
| **Assigned group** |  |  |  |  |  |  |  |  |  |
| Normal |  | 1025 | 67.9 |  | 907 | 62.9 |  | 493.01 (3) | < .001 |
| Training |  | 3 | 0.2 |  | 334 | 23.2 |  |  |  |
| NoTraining |  | 451 | 29.9 |  | 146 | 10.1 |  |  |  |
| Abnormal |  | 31 | 2.1 |  | 54 | 3.7 |  |  |  |
| **Training group** |  |  |  |  |  |  |  |  |  |
| Baghira |  | 1 | 0.2 |  | 191 | 39.8 |  | 480.82 (3) | < .001 |
| Tiger |  | 2 | 0.4 |  | 143 | 29.8 |  |  |  |
| NoBaghira |  | 268 | 59.0 |  | 83 | 17.3 |  |  |  |
| NoTiger |  | 183 | 40.3 |  | 63 | 13.1 |  |  |  |
| **Child’s sex** |  |  |  |  |  |  |  |  |  |
| female |  | 748 | 51.5 |  | 692 | 48.2 |  | 3.13 (1) | .077 |
| male |  | 705 | 48.5 |  | 744 | 51.8 |  |  |  |
| **Child’s age at screening** |  | 1494 | 6.7 (1.97) |  | 1432 | 6.7 (1.92) |  | -0.45 (2924) | .650 |
| **Nationality child** |  |  |  |  |  |  |  |  |  |
| German |  | 974 | 99.2 |  | 1115 | 98.8 |  | 0.61 (1) | .436 |
| Other |  | 8 | 0.8 |  | 13 | 1.2 |  | 0.61 (1) | .436 |
| **Nationality mother** |  |  |  |  |  |  |  |  |  |
| German |  | 942 | 95.7 |  | 1098 | 97.1 |  | 2.81 (1) | .094 |
| Other |  | 42 | 4.3 |  | 33 | 2.9 |  | 2.81 (1) | .094 |
| **Nationality father** |  |  |  |  |  |  |  |  |  |
| German |  | 879 | 95.8 |  | 1041 | 97.3 |  | 3.54 (1) | .060 |
| Other |  | 39 | 4.2 |  | 29 | 2.7 |  | 3.54 (1) | .060 |
| **Parents' monthly net income** |  |  |  |  |  |  |  |  |  |
| less than 1000€ |  | 9 | 1.1 |  | 17 | 1.7 |  | 15.73 (4) | .003 |
| 1000-2000€ |  | 149 | 18.1 |  | 128 | 12.5 |  |  |  |
| 2000-3000€ |  | 167 | 20.3 |  | 188 | 18.4 |  |  |  |
| 3000-4000€ |  | 277 | 33.7 |  | 363 | 35.5 |  |  |  |
| more than 4000€ |  | 221 | 26.9 |  | 326 | 31.9 |  |  |  |
| **SDQ emotional problems at screening** |  | 1504 | 1.8 (1.84) |  | 1426 | 2.2 (2.17) |  | -5.55 (2928) | < .001 |
| **SDQ conduct problems at screening** |  | 1504 | 1.7 (1.61) |  | 1426 | 1.9 (1.82) |  | -2.72 (2928) | .007 |

*Note.* Included = data of each KINDL sub-scale was available at least at one measurement time point; Excluded = data of a KINDL sub-scale was not available at any measuremnt time point; KINDL = Kiddy-KINDL-R and Kid-/Kiddo-KINDL-R Quality of Life Questionnaire for Children, SDQ = Strengths and Difficulties Questionnaire; Normal = children evaluated as normal with no recommendation for indicated prevention participation; Training = children who participated in an indicated prevention program after recommendation, NoTraining = children who did not participate in an indicated prevention program despite recommendation; Abnormal = children with abnormal or clinically significant disruptive behavior or emotional problems or that did not fullfill inclusion criteria for participation in an indicated prevention program; Baghira = children who participated in the Baghira training; Tiger = children who participated in the Tiger training; NoBaghira = children who did not participate in the Baghira training despite a recommendation including children with a recommendation for both trainings and a higher or equal SDQ conduct problems than emotional problems score; NoTiger = children who did not participate in the Tiger training despite a recommendation including children with a recommendation for both trainings and a higher SDQ emotional problems than conduct problems score; n = sample size/number of participants; M = mean; SD = standard deviation; df = degrees of freedom; p = p-value

Table S6. Comparison of children with complete vs. incomplete data sets in the SDQ analysis sample

|  |  | **Non-completer** | |  | **Completer** | |  | **Comparison** | |
| --- | --- | --- | --- | --- | --- | --- | --- | --- | --- |
|  |  | n = 2136 | |  | n = 809 | |  |  | |
|  |  | **n** | **% /**  **M (SD)** |  | **n** | **% /**  **M (SD)** |  | **t (df) /**  **Chi2 (df)** | **p** |
| **Assigned group** |  |  |  |  |  |  |  |  |  |
| Normal |  | 1426 | 66.8 |  | 502 | 62.1 |  | 414.67 (3) | < .001 |
| Training |  | 103 | 4.8 |  | 234 | 28.9 |  |  |  |
| No Training |  | 548 | 25.7 |  | 47 | 5.8 |  |  |  |
| Abnormal |  | 59 | 2.8 |  | 26 | 3.2 |  |  |  |
| **Training group** |  |  |  |  |  |  |  |  |  |
| Baghira |  | 59 | 9.1 |  | 133 | 47.33 |  | 386.87 (3) | < .001 |
| Tiger |  | 44 | 6.8 |  | 101 | 35.94 |  |  |  |
| NoBaghira |  | 322 | 49.5 |  | 28 | 9.96 |  |  |  |
| NoTiger |  | 226 | 34.7 |  | 19 | 6.76 |  |  |  |
| **Child’s sex** |  |  |  |  |  |  |  |  |  |
| female |  | 1065 | 51.3 |  | 371 | 45.9 |  | 6.81 (1) | .009 |
| male |  | 1010 | 48.7 |  | 437 | 54.1 |  |  |  |
| **Child’s age at screening** |  | 2115 | 6.7 (1.95) |  | 808 | 6.8 (1.94) |  | -1.34 (2921) | .180 |
| **Nationality child** |  |  |  |  |  |  |  |  |  |
| German |  | 1439 | 99.1 |  | 647 | 98.8 |  | 0.49 (1) | .486 |
| Other |  | 13 | 0.9 |  | 8 | 1.2 |  |  |  |
| **Nationality mother** |  |  |  |  |  |  |  |  |  |
| German |  | 1393 | 96.0 |  | 644 | 97.4 |  | 2.69 (1) | .101 |
| Other |  | 58 | 4.0 |  | 17 | 2.6 |  |  |  |
| **Nationality father** |  |  |  |  |  |  |  |  |  |
| German |  | 1303 | 96.3 |  | 614 | 97.2 |  | 0.93 (1) | .334 |
| Other |  | 50 | 3.7 |  | 18 | 2.8 |  |  |  |
| **Parents' monthly net income** |  |  |  |  |  |  |  |  |  |
| less than 1000€ |  | 20 | 1.6 |  | 6 | 1.0 |  | 11.80 (4) | .019 |
| 1000-2000€ |  | 195 | 15.8 |  | 80 | 13.2 |  |  |  |
| 2000-3000€ |  | 253 | 20.4 |  | 102 | 16.9 |  |  |  |
| 3000-4000€ |  | 430 | 34.7 |  | 210 | 34.7 |  |  |  |
| more than 4000€ |  | 340 | 27.5 |  | 207 | 34.2 |  |  |  |
| **SDQ emotional problems at screening** |  | 2121 | 1.8 (1.91) |  | 809 | 2.3 (2.24) |  | -5.21 (2928) | < .001 |
| **SDQ conduct problems at screening** |  | 2121 | 1.8 (1.66) |  | 809 | 1.9 (1.86) |  | -2.27 (2928) | .024 |

*Note.* Non-Completer = data of SDQ emotional problems and conduct problems sub-scales were available at least at one but not all measurement time points; Completer = data of SDQ emotional problems and conduct problems sub-scales were available at all measuremnt time points; SDQ = Strengths and Difficulties Questionnaire; Normal = children evaluated as normal with no recommendation for indicated prevention participation; Training = children who participated in an indicated prevention program after recommendation, NoTraining = children who did not participate in an indicated prevention program despite recommendation; Abnormal = children with abnormal or clinically significant disruptive behavior or emotional problems or that did not fullfill inclusion criteria for participation in an indicated prevention program; Baghira = children who participated in the Baghira training; Tiger = children who participated in the Tiger training; NoBaghira = children who did not participate in the Baghira training despite a recommendation including children with a recommendation for both trainings and a higher or equal SDQ conduct problems than emotional problems score; NoTiger = children who did not participate in the Tiger training despite a recommendation including children with a recommendation for both trainings and a higher SDQ emotional problems than conduct problems score; n = sample size/number of participants; M = mean; SD = standard deviation; df = degrees of freedom; p = p-value

Table S7. Comparison of children with complete vs. incomplete data sets in the KINDL analysis sample

|  |  | **Non-completer** | |  | **Completer** | |  | **Comparison** | |
| --- | --- | --- | --- | --- | --- | --- | --- | --- | --- |
|  |  | n = 711 | |  | n = 730 | |  |  | |
|  |  | **n** | **% /**  **M (SD)** |  | **n** | **% /**  **M (SD)** |  | **t (df) /**  **Chi2 (df)** | **p** |
| **Assigned group** |  |  |  |  |  |  |  |  |  |
| Normal |  | 460 | 64.7 |  | 447 | 61.2 |  | 91.75 (3) | < .001 |
| Training |  | 106 | 14.9 |  | 228 | 31.2 |  |  |  |
| No Training |  | 114 | 16.0 |  | 32 | 4.4 |  |  |  |
| Abnormal |  | 31 | 4.4 |  | 23 | 3.2 |  |  |  |
| **Training group** |  |  |  |  |  |  |  |  |  |
| Baghira |  | 64 | 29.1 |  | 127 | 48.8 |  | 89.00 (3) | < .001 |
| Tiger |  | 42 | 19.1 |  | 101 | 38.8 |  |  |  |
| NoBaghira |  | 67 | 30.5 |  | 16 | 6.2 |  |  |  |
| NoTiger |  | 47 | 21.4 |  | 16 | 6.2 |  |  |  |
| **Child’s sex** |  |  |  |  |  |  |  |  |  |
| female |  | 356 | 50.4 |  | 336 | 46.1 |  | 2.61 (1) | .106 |
| male |  | 351 | 49.6 |  | 393 | 53.9 |  |  |  |
| **Child’s age at screening** |  | 706 | 6.6 (1.89) |  | 726 | 6.8 (1.95) |  | -1.54 (1430) | .124 |
| **Nationality child** |  |  |  |  |  |  |  |  |  |
| German |  | 524 | 98.9 |  | 591 | 98.8 |  | 0.00 (1) | .952 |
| Other |  | 6 | 1.1 |  | 7 | 1.2 |  |  |  |
| **Nationality mother** |  |  |  |  |  |  |  |  |  |
| German |  | 509 | 96.6 |  | 589 | 97.5 |  | 0.86 (1) | .353 |
| Other |  | 18 | 3.4 |  | 15 | 2.5 |  |  |  |
| **Nationality father** |  |  |  |  |  |  |  |  |  |
| German |  | 478 | 97.0 |  | 563 | 97.6 |  | 0.38 (1) | .536 |
| Other |  | 15 | 3.0 |  | 14 | 2.4 |  |  |  |
| **Parents' monthly net income** |  |  |  |  |  |  |  |  |  |
| less than 1000€ |  | 12 | 2.6 |  | 5 | 0.9 |  | 7.52 (4) | .111 |
| 1000-2000€ |  | 55 | 11.8 |  | 73 | 13.1 |  |  |  |
| 2000-3000€ |  | 93 | 20.0 |  | 95 | 17.1 |  |  |  |
| 3000-4000€ |  | 168 | 36.1 |  | 195 | 35.0 |  |  |  |
| more than 4000€ |  | 137 | 29.5 |  | 189 | 33.9 |  |  |  |
| **KINDL physical well-being at T0** |  | 488 | 17.5 (2.37) |  | 730 | 17.6 (2.09) |  | -1.08 (1216) | .281 |
| **KINDL emotional well-being at T0** |  | 487 | 17.0 (2.12) |  | 730 | 16.8 (2.08) |  | 1.39 (1215) | .165 |
| **KINDL self-esteem at T0** |  | 487 | 16.0 (2.29) |  | 730 | 16.0 (2.33) |  | 0.44 (1215) | .657 |
| **KINDL family at T0** |  | 487 | 16.6 (2.22) |  | 730 | 16.7 (2.07) |  | -1.09 (1215) | .275 |
| **KINDL friends at T0** |  | 487 | 16.4 (2.37) |  | 730 | 16.4 (2.50) |  | 0.50 (1215) | .621 |
| **KINDL everyday functioning at T0** |  | 481 | 16.9 (2.40) |  | 730 | 16.9 (2.42) |  | -0.53 (1209) | .596 |

*Note.* Non-Completer = data of the KINDL sub-scales were available at least at one but not all measurement time points; Completer = data of the KINDL sub-scales were available at all measuremnt time points; KINDL = Kiddy-KINDL-R and Kid-/Kiddo-KINDL-R Quality of Life Questionnaire for Children, Normal = children evaluated as normal with no recommendation for indicated prevention participation; Training = children who participated in an indicated prevention program after recommendation, NoTraining = children who did not participate in an indicated prevention program despite recommendation; Abnormal = children with abnormal or clinically significant disruptive behavior or emotional problems or that did not fullfill inclusion criteria for participation in an indicated prevention program; Baghira = children who participated in the Baghira training; Tiger = children who participated in the Tiger training; NoBaghira = children who did not participate in the Baghira training despite a recommendation including children with a recommendation for both trainings and a higher or equal SDQ conduct problems than emotional problems score; NoTiger = children who did not participate in the Tiger training despite a recommendation including children with a recommendation for both trainings and a higher SDQ emotional problems than conduct problems score; n = sample size/number of participants; M = mean; SD = standard deviation; df = degrees of freedom; p = p-value

Table S8. Six-month prevalence of disruptive behavior and emotional problems in children from the general population^1^ using PROMPt Project adapted SDQ cut-offs.

|  |  | **Total** | |  | **By child’s sex**^2^ | | | |  | **By child’s age** | | | |
| --- | --- | --- | --- | --- | --- | --- | --- | --- | --- | --- | --- | --- | --- |
|  |  | (n = 2825) | |  | **female**  (n = 1403) | | **male**  (n = 1360) | |  | **3-6 years**  (n = 1439) | | **7-11 years**  (n = 1386) | |
|  |  | **n** | **%** |  | **n** | **%** | **n** | **%** |  | **n** | **%** | **n** | **%** |
| **SDQ conduct problems** |  |  |  |  |  |  |  |  |  |  |  |  |  |
| normal (score 0-2) |  | 2055 | 72.7 |  | 1096 | 78.1 | 906 | 66.6 |  | 1016 | 70.6 | 1039 | 75.0 |
| borderline (score 3-5) |  | 683 | 24.2 |  | 283 | 20.2 | 392 | 28.8 |  | 393 | 27.3 | 290 | 20.9 |
| abnormal (score 6-10) |  | 87 | 3.1 |  | 24 | 1.7 | 62 | 4.6 |  | 30 | 2.1 | 57 | 4.1 |
|  |  |  |  |  | Chi2(2) = 51.77, p < .001 | | | |  | Chi2(2) = 23.18, p < .001 | | | |
| **SDQ emotional problems** |  |  |  |  |  |  |  |  |  |  |  |  |  |
| normal (score 0-3) |  | 2316 | 82.0 |  | 1126 | 80.3 | 1136 | 83.5 |  | 1232 | 85.6 | 1084 | 78.2 |
| borderline (score 4-6) |  | 425 | 15.0 |  | 233 | 16.6 | 186 | 13.7 |  | 176 | 12.2 | 249 | 18.0 |
| abnormal (score 7-10) |  | 84 | 3.0 |  | 44 | 3.1 | 38 | 2.8 |  | 31 | 2.2 | 53 | 3.8 |
|  |  |  |  |  | Chi2(2) = 5.09, p = .079 | | | |  | Chi2(2) = 26.77, p < .001 | | | |
| **SDQ conduct problems and emotional problems combined** |  |  |  |  |  |  |  |  |  |  |  |  |  |
| normal (= normal in both scores) |  | 1781 | 63.0 |  | 929 | 66.2 | 805 | 59.2 |  | 893 | 62.1 | 888 | 64.1 |
| borderline disruptive behavior problems only (= borderline conduct problems score and normal emotional problems score) |  | 492 | 17.4 |  | 189 | 13.5 | 296 | 21.8 |  | 319 | 22.2 | 173 | 12.5 |
| borderline emotional problems only (= borderline emotional problems score and normal conduct problems score) |  | 238 | 8.4 |  | 145 | 10.3 | 89 | 6.5 |  | 109 | 7.6 | 129 | 9.3 |
| borderline disruptive behavior & emotional problems (= borderline in both scores) |  | 157 | 5.6 |  | 79 | 5.6 | 77 | 5.7 |  | 60 | 4.2 | 97 | 7.0 |
| abnormal (= abnormal in either one of the scores) |  | 157 | 5.6 |  | 61 | 4.3 | 93 | 6.8 |  | 58 | 4.0 | 99 | 7.1 |
|  |  |  |  |  | Chi2(4) = 51.98, p < .001 | | | |  | Chi2(4) = 63.47, p < .001 | | | |

*Note.* ^1^Subjects who entered the project via other access routes than the screening at the pediatrician are excluded. ^2^Sex of 62 children is unknown. SDQ = Strengths and Difficulties Questionnaire; n = number of participants; p = p-value

Table S9. Six-month prevalence of disruptive behavior and emotional problems in children from the general population including subjects who entered the project via other access routes than the screening at the pediatrician using SDQ cut-offs by Goodman (1997).

|  |  | **Total** | |  | **By child’s sex**^1^ | | | |  | **By child’s age** | | | |
| --- | --- | --- | --- | --- | --- | --- | --- | --- | --- | --- | --- | --- | --- |
|  |  | (n = 2939) | |  | **female**  (n = 1432) | | **male**  (n = 1445) | |  | **3-6 years**  (n = 1494) | | **7-11 years**  (n = 1445) | |
|  |  | **n** | **%** |  | **n** | **%** | **n** | **%** |  | **n** | **%** | **n** | **%** |
| **SDQ conduct problems** |  |  |  |  |  |  |  |  |  |  |  |  |  |
| normal (score 0-2) |  | 2099 | 71.4 |  | 1113 | 77.7 | 933 | 64.6 |  | 1040 | 69.6 | 1059 | 73.3 |
| borderline (score 3) |  | 381 | 13.0 |  | 162 | 11.3 | 214 | 14.8 |  | 218 | 14.6 | 163 | 11.3 |
| abnormal (score 4-10) |  | 459 | 15.6 |  | 157 | 11.0 | 298 | 20.6 |  | 236 | 15.8 | 223 | 15.4 |
|  |  |  |  |  | Chi2(2) = 66.66, p < .001 | | | |  | Chi2(2) = 7.66, p = .022 | | | |
| **SDQ emotional problems** |  |  |  |  |  |  |  |  |  |  |  |  |  |
| normal (score 0-3) |  | 2358 | 80.2 |  | 1133 | 79.1 | 1171 | 81.0 |  | 1251 | 83.7 | 1107 | 76.6 |
| borderline (score 4) |  | 218 | 7.4 |  | 115 | 8.0 | 98 | 6.8 |  | 89 | 6.0 | 129 | 8.9 |
| abnormal (score 5-10) |  | 363 | 12.4 |  | 184 | 12.8 | 176 | 12.2 |  | 154 | 10.3 | 209 | 14.5 |
|  |  |  |  |  | Chi2(2) = 2.10, p = .349 | | | |  | Chi2(2) = 23.66, p < .001 | | | |
| **SDQ conduct problems and emotional problems combined** |  |  |  |  |  |  |  |  |  |  |  |  |  |
| normal (= normal in both scores) |  | 1791 | 60.9 |  | 932 | 65.1 | 812 | 56.2 |  | 898 | 60.1 | 893 | 61.8 |
| borderline disruptive behavior problems only (= borderline conduct problems score and normal emotional problems score) |  | 282 | 9.6 |  | 114 | 8.0 | 164 | 11.3 |  | 183 | 12.2 | 99 | 6.9 |
| borderline emotional problems only (= borderline emotional problems score and normal conduct problems score) |  | 131 | 4.5 |  | 78 | 5.4 | 50 | 3.5 |  | 61 | 4.1 | 70 | 4.8 |
| borderline disruptive behavior & emotional problems (= borderline in both scores) |  | 47 | 1.6 |  | 21 | 1.5 | 25 | 1.7 |  | 12 | 0.8 | 35 | 2.4 |
| abnormal (= abnormal in either one of the scores) |  | 688 | 23.4 |  | 287 | 20.0 | 394 | 27.3 |  | 340 | 22.8 | 348 | 24.1 |
|  |  |  |  |  | Chi2(4) = 40.48, p < .001 | | | |  | Chi2(4) = 36.20, p < .001 | | | |

*Note.* ^1^Sex of 62 children is unknown. SDQ = Strengths and Difficulties Questionnaire; n = number of participants; p = p-value

Table S10. Six-month prevalence of disruptive behavior and emotional problems combined in children from the general population including subjects who entered the project via other access routes than the screening at the pediatrician using PROMPt Project adapted SDQ cut-offs.

|  |  | **Total** | |  | **By child’s sex**^1^ | | | |  | **By child’s age** | | | |
| --- | --- | --- | --- | --- | --- | --- | --- | --- | --- | --- | --- | --- | --- |
|  |  | (n = 2939) | |  | **female**  (n = 1432) | | **male**  (n = 1445) | |  | **3-6 years**  (n = 1494) | | **7-11 years**  (n = 1445) | |
|  |  | **n** | **%** |  | **n** | **%** | **n** | **%** |  | **n** | **%** | **n** | **%** |
| **SDQ conduct problems** |  |  |  |  |  |  |  |  |  |  |  |  |  |
| normal (score 0-2) |  | 2099 | 71.4 |  | 1113 | 77.7 | 933 | 64.6 |  | 1040 | 69.6 | 1059 | 73.3 |
| borderline (score 3-5) |  | 737 | 25.1 |  | 294 | 20.5 | 435 | 30.1 |  | 419 | 28.0 | 318 | 22.0 |
| abnormal (score 6-10) |  | 103 | 3.5 |  | 25 | 1.7 | 77 | 5.3 |  | 35 | 2.3 | 68 | 4.7 |
|  |  |  |  |  | Chi2(2) = 69.56, p < .001 | | | |  | Chi2(2) = 23.78, p < .001 | | | |
| **SDQ emotional problems** |  |  |  |  |  |  |  |  |  |  |  |  |  |
| normal (score 0-3) |  | 2358 | 80.2 |  | 1133 | 79.1 | 1171 | 81.0 |  | 1251 | 83.7 | 1107 | 76.6 |
| borderline (score 4-6) |  | 468 | 15.9 |  | 245 | 17.1 | 217 | 15.0 |  | 197 | 13.2 | 271 | 18.8 |
| abnormal (score 7-10) |  | 113 | 3.8 |  | 54 | 3.8 | 57 | 3.9 |  | 46 | 3.1 | 67 | 4.6 |
|  |  |  |  |  | Chi2(2) = 2.35, p = .309 | | | |  | Chi2(2) = 23.59, p < .001 | | | |
| **SDQ conduct problems and emotional problems combined** |  |  |  |  |  |  |  |  |  |  |  |  |  |
| normal (= normal in both scores) |  | 1791 | 60.9 |  | 932 | 65.1 | 812 | 56.2 |  | 898 | 60.1 | 893 | 61.8 |
| borderline disruptive behavior problems only (= borderline conduct problems score and normal emotional problems score) |  | 518 | 17.6 |  | 192 | 13.4 | 319 | 22.1 |  | 333 | 22.3 | 185 | 12.8 |
| borderline emotional problems only (= borderline emotional problems score and normal conduct problems score) |  | 264 | 9.0 |  | 154 | 10.8 | 106 | 7.3 |  | 124 | 8.3 | 140 | 9.7 |
| borderline disruptive behavior & emotional problems (= borderline in both scores) |  | 169 | 5.8 |  | 82 | 5.7 | 86 | 6.0 |  | 63 | 4.2 | 106 | 7.3 |
| abnormal (= abnormal in either one of the scores) |  | 197 | 6.7 |  | 72 | 5.0 | 122 | 8.4 |  | 76 | 5.1 | 121 | 8.4 |
|  |  |  |  |  | Chi2(4) = 61.61, p < .001 | | | |  | Chi2(4) = 63.69, p < .001 | | | |

*Note.* ^1^Sex of 62 children is unknown. SDQ = Strengths and Difficulties Questionnaire; n = number of participants; p = p-value

Table S11. Quality of life in children from the general population including subjects who entered the project via other access routes than the screening at the pediatrician.

|  |  | **Physical  well-being** | |  | **Emotional  well-being** | |  | **Self-esteem** | |  | **Family** | |  | **Friends** | |  | **Everyday functioning** | |
| --- | --- | --- | --- | --- | --- | --- | --- | --- | --- | --- | --- | --- | --- | --- | --- | --- | --- | --- |
|  |  | **n** | **M (SD)** |  | **n** | **M (SD)** |  | **n** | **M (SD)** |  | **n** | **M (SD)** |  | **n** | **M (SD)** |  | **N** | **M (SD)** |
| **Total sample** |  | 1210 | 17.6 (2.21) |  | 1208 | 16.9 (2.10) |  | 1208 | 16.0 (2.33) |  | 1208 | 16.7 (2.13) |  | 1207 | 16.4 (2.46) |  | 1198 | 16.9 (2.41) |
| **By child’s sex** |  |  |  |  |  |  |  |  |  |  |  |  |  |  |  |  |  |  |
| female |  | 582 | 17.4 (2.28) |  | 581 | 17.0 (2.12) |  | 581 | 16.3 (2.27) |  | 581 | 17.0 (2.05) |  | 581 | 16.7 (2.28) |  | 577 | 17.2 (2.40) |
| male |  | 623 | 17.7 (2.14) |  | 622 | 16.8 (2.08) |  | 622 | 15.7 (2.35) |  | 622 | 16.4 (2.18) |  | 621 | 16.1 (2.59) |  | 616 | 16.6 (2.39) |
|  |  | t(1203) = -2.10  p = .036 | |  | t(1201) = 1.78  p = .075 | |  | t(1201) = 4.76  p < .001 | |  | t(1201) = 4.29  p < .001 | |  | t(1200) = 4.51  p < .001 | |  | t(1191) = 4.24  p < .001 | |
| **By age group** |  |  |  |  |  |  |  |  |  |  |  |  |  |  |  |  |  |  |
| 3-6 years |  | 610 | 17.8 (2.09) |  | 609 | 17.2 (1.97) |  | 609 | 16.3 (2.04) |  | 609 | 16.7 (2.04) |  | 609 | 16.6 (2.20) |  | 602 | 17.0 (2.45) |
| 7-11 years |  | 600 | 17.4 (2.31) |  | 599 | 16.6 (2.20) |  | 599 | 15.7 (2.56) |  | 599 | 16.7 (2.22) |  | 598 | 16.2 (2.69) |  | 596 | 16.7 (2.37) |
|  |  | t(1208) = 2.99  p = .003 | |  | t(1206) = 4.32  p < .001 | |  | t(1206) = 4.83  p < .001 | |  | t(1206) = 0.18  p = .861 | |  | t(1205) = 2.69  p = .007 | |  | (1196) = 2.17  p = .031 | |
| **By SDQ screening result with cut-offs by Goodman (1997)** |  |  |  |  |  |  |  |  |  |  |  |  |  |  |  |  |  |  |
| normal |  | 696 | 17.9 (1.96) |  | 696 | 17.6 (1.66) |  | 696 | 16.7 (2.00) |  | 696 | 17.4 (1.82) |  | 695 | 17.0 (2.11) |  | 690 | 17.5 (2.15) |
| borderline disruptive behavior problems only |  | 89 | 17.4 (2.27) |  | 89 | 17.0 (1.85) |  | 89 | 16.0 (2.30) |  | 89 | 16.2 (1.96) |  | 89 | 16.4 (2.01) |  | 89 | 16.8 (2.31) |
| borderline emotional problems only |  | 52 | 17.3 (2.01) |  | 51 | 16.4 (1.63) |  | 51 | 15.6 (1.83) |  | 51 | 16.9 (1.85) |  | 51 | 16.5 (1.97) |  | 50 | 16.3 (2.33) |
| borderline disruptive behavior & emotional problems |  | 19 | 16.7 (2.96) |  | 19 | 15.2 (1.98) |  | 19 | 14.6 (2.99) |  | 19 | 15.3 (1.92) |  | 19 | 14.4 (3.76) |  | 19 | 15.5 (2.50) |
| abnormal |  | 354 | 17.0 (2.48) |  | 353 | 15.7 (2.37) |  | 353 | 14.7 (2.37) |  | 353 | 15.5 (2.25) |  | 353 | 15.2 (2.68) |  | 350 | 15.9 (2.59) |
|  |  | Chi2(4) = 41.60  p < .001 | |  | Chi2(4) = 186.73  p < .001 | |  | Chi2(4) = 182.72  p < .001 | |  | Chi2(4) = 177.79  p < .001 | |  | Chi2(4) = 140.24  p < .001 | |  | Chi2(4) = 104.20  p < .001 | |

Note. KINDL scores were calculated as sum scores ranging from 0 to 20. Quality of life domains assessed with the Kiddy-KINDL-R and Kid-/Kiddo-KINDL-R Quality of Life Questionnaire for Children at T0. SDQ = Strengths and Difficulties Questionnaire; n = number of participants; M = mean; SD = standard deviation

Table S12. Quality of life in children from the general population^1^ with KINDL scores as percentile ranks.

|  |  | **Physical  well-being** | |  | **Emotional  well-being** | |  | **Self-Esteem** | |  | **Family** | |  | **Friends** | |  | **Everyday functioning** | |
| --- | --- | --- | --- | --- | --- | --- | --- | --- | --- | --- | --- | --- | --- | --- | --- | --- | --- | --- |
|  |  | **n** | **M (SD)** |  | **n** | **M (SD)** |  | **n** | **M (SD)** |  | **n** | **M (SD)** |  | **n** | **M (SD)** |  | **n** | **M (SD)** |
| **Total sample** |  | 1104 | 85.3 (13.57) |  | 1102 | 81.4 (12.80) |  | 1102 | 75.8 (14.37) |  | 1102 | 79.9 (13.20) |  | 1101 | 78.3 (14.96) |  | 1092 | 81.0 (14.98) |
| **By child’s sex** |  |  |  |  |  |  |  |  |  |  |  |  |  |  |  |  |  |  |
| female |  | 555 | 84.2 (14.14) |  | 554 | 81.9 (12.93) |  | 554 | 77.7 (13.97) |  | 554 | 81.2 (12.78) |  | 554 | 80.0 (13.97) |  | 550 | 82.8 (14.95) |
| male |  | 544 | 86.4 (12.92) |  | 543 | 80.9 (12.66) |  | 543 | 74.0 (14.56) |  | 543 | 78.5 (13.52) |  | 542 | 76.5 (15.73) |  | 537 | 79.3 (14.83) |
|  |  | t(1097) = -2.67  p = .008 | |  | t(1095) = 1.29  p = .196 | |  | t(1095) = 4.33  p < .001 | |  | t(1095) = 3.49  p < .001 | |  | t(1094) = 3.93  p < .001 | |  | t(1085) = 3.82  p < .001 | |
| **By age group** |  |  |  |  |  |  |  |  |  |  |  |  |  |  |  |  |  |  |
| 3-6 years |  | 557 | 86.4 (12.98) |  | 556 | 83.1 (11.88) |  | 556 | 77.8 (12.60) |  | 556 | 79.9 (12.66) |  | 556 | 79.5 (13.50) |  | 549 | 82.2 (15.08) |
| 7-11 years |  | 547 | 84.2 (14.07) |  | 546 | 79.6 (13.47) |  | 546 | 73.8 (15.73) |  | 546 | 79.9 (13.75) |  | 545 | 77.0 (16.24) |  | 543 | 79.8 (14.79) |
|  |  | t(1102) = 2.82  p = .005 | |  | t(1100) = 4.54  p < .001 | |  | t(1100) = 4.60  p < .001 | |  | t(1100) = -0.11  p = .911 | |  | t(1099) = 2.72  p = .007 | |  | (1090) = 2.57  p = .010 | |
| **By SDQ screening result with cut-offs by Goodman (1997)** |  |  |  |  |  |  |  |  |  |  |  |  |  |  |  |  |  |  |
| normal |  | 686 | 87.1 (12.30) |  | 686 | 85.0 (10.37) |  | 686 | 79.6 (12.50) |  | 686 | 83.5 (11.41) |  | 685 | 81.6 (13.13) |  | 680 | 84.2 (13.48) |
| borderline disruptive behavior problems only |  | 84 | 84.2 (13.53) |  | 84 | 81.5 (11.31) |  | 84 | 75.2 (14.17) |  | 84 | 76.6 (12.24) |  | 84 | 77.8 (12.77) |  | 84 | 80.2 (14.80) |
| borderline emotional problems only |  | 43 | 81.8 (13.07) |  | 42 | 78.1 (9.79) |  | 42 | 72.2 (11.57) |  | 42 | 79.6 (10.96) |  | 42 | 79.0 (11.37) |  | 41 | 76.5 (15.55) |
| borderline disruptive behavior & emotional problems |  | 18 | 82.3 (14.74) |  | 18 | 70.8 (11.74) |  | 18 | 69.1 (15.08) |  | 18 | 70.8 (12.31) |  | 18 | 65.6 (24.09) |  | 18 | 72.6 (15.92) |
| abnormal |  | 273 | 81.8 (15.71) |  | 272 | 73.6 (15.13) |  | 272 | 67.3 (15.29) |  | 272 | 72.4 (14.38) |  | 272 | 70.7 (16.48) |  | 269 | 74.3 (15.92) |
|  |  | Chi2(4) = 31.14  p < .001 | |  | Chi2(4) = 141.87  p < .001 | |  | Chi2(4) = 140.91  p < .001 | |  | Chi2(4) = 143.37  p < .001 | |  | Chi2(4) = 107.53  p < .001 | |  | Chi2(4) = 95.59  p < .001 | |

Note. KINDL scores were calculated as percentile ranks on a scale of 0 to 100. Quality of life domains assessed with the Kiddy-KINDL-R and Kid-/Kiddo-KINDL-R Quality of Life Questionnaire for Children at T0. ^1^Subjects who entered the project via other access routes than the screening at the pediatrician are excluded. SDQ = Strengths and Difficulties Questionnaire; n = number of participants; M = mean; SD = standard deviation

Table S13. Quality of life in children from the general population including subjects who entered the project via other access routes than the screening at the pediatrician with KINDL scores as percentile ranks.

|  |  | **Physical  well-being** | |  | **Emotional  well-being** | |  | **Self-esteem** | |  | **Family** | |  | **Friends** | |  | **Everyday functioning** | |
| --- | --- | --- | --- | --- | --- | --- | --- | --- | --- | --- | --- | --- | --- | --- | --- | --- | --- | --- |
|  |  | **n** | **M (SD)** |  | **n** | **M (SD)** |  | **n** | **M (SD)** |  | **n** | **M (SD)** |  | **n** | **M (SD)** |  | **n** | **M (SD)** |
| **Total sample** |  | 1210 | 84.9 (13.80) |  | 1208 | 80.6 (13.14) |  | 1208 | 75.0 (14.58) |  | 1208 | 79.4 (13.32) |  | 1207 | 77.4 (15.40) |  | 1198 | 80.6 (15.09) |
| **By child’s sex** |  |  |  |  |  |  |  |  |  |  |  |  |  |  |  |  |  |  |
| female |  | 582 | 84.0 (14.25) |  | 581 | 81.3 (13.27) |  | 581 | 77.1 (14.20) |  | 581 | 81.0 (12.80) |  | 581 | 79.4 (14.27) |  | 577 | 82.5 (15.03) |
| male |  | 623 | 85.7 (13.35) |  | 622 | 79.9 (12.98) |  | 622 | 73.1 (14.71) |  | 622 | 77.8 (13.63) |  | 621 | 75.5 (16.18) |  | 616 | 78.9 (14.96) |
|  |  | t(1203) = -2.10  p = .036 | |  | t(1201) = 1.78  p = .075 | |  | t(1201) = 4.76  p < .001 | |  | t(1201) = 4.29  p < .001 | |  | t(1200) = 4.51  p < .001 | |  | t(1191) = 4.24  p < .001 | |
| **By age group** |  |  |  |  |  |  |  |  |  |  |  |  |  |  |  |  |  |  |
| 3-6 years |  | 610 | 86.1 (13.03) |  | 609 | 82.2 (12.30) |  | 609 | 77.0 (12.72) |  | 609 | 79.5 (12.77) |  | 609 | 78.6 (13.77) |  | 602 | 81.6 (15.30) |
| 7-11 years |  | 600 | 83.7 (14.45) |  | 599 | 79.0 (13.76) |  | 599 | 72.9 (16.01) |  | 599 | 79.3 (13.86) |  | 598 | 76.2 (16.83) |  | 596 | 79.7 (14.82) |
|  |  | t(1208) = 2.99  p = .003 | |  | t(1206) = 4.32  p < .001 | |  | t(1206) = 4.83  p < .001 | |  | t(1206) = 0.18  p = .861 | |  | t(1205) = 2.69  p = .007 | |  | (1196) = 2.17  p = .031 | |
| **By SDQ screening result with cut-offs by Goodman (1997)** |  |  |  |  |  |  |  |  |  |  |  |  |  |  |  |  |  |  |
| normal |  | 696 | 87.2 (12.26) |  | 696 | 84.9 (10.35) |  | 696 | 79.6 (12.50) |  | 696 | 83.5 (11.36) |  | 695 | 81.5 (13.21) |  | 690 | 84.2 (13.42) |
| borderline disruptive behavior problems only |  | 89 | 83.6 (14.18) |  | 89 | 81.3 (11.58) |  | 89 | 74.7 (14.35) |  | 89 | 76.5 (12.22) |  | 89 | 77.4 (12.55) |  | 89 | 80.0 (14.45) |
| borderline emotional problems only |  | 52 | 83.4 (12.55) |  | 51 | 77.5 (10.16) |  | 51 | 72.8 (11.44) |  | 51 | 80.8 (11.58) |  | 51 | 78.1 (12.33) |  | 50 | 77.0 (14.59) |
| borderline disruptive behavior & emotional problems |  | 19 | 79.6 (18.50) |  | 19 | 69.7 (12.37) |  | 19 | 66.4 (18.66) |  | 19 | 70.7 (11.98) |  | 19 | 65.1 (23.51) |  | 19 | 72.0 (15.64) |
| abnormal |  | 354 | 81.4 (15.52) |  | 353 | 72.9 (14.83) |  | 353 | 66.8 (14.80) |  | 353 | 72.2 (14.04) |  | 353 | 69.8 (16.75) |  | 350 | 74.7 (16.20) |
|  |  | Chi2(4) = 41.60  p < .001 | |  | Chi2(4) = 186.73  p < .001 | |  | Chi2(4) = 182.72  p < .001 | |  | Chi2(4) = 177.79  p < .001 | |  | Chi2(4) = 140.24  p < .001 | |  | Chi2(4) = 140.20  p < .001 | |

Note. KINDL scores were calculated as percentile ranks on a scale of 0 to 100. Quality of life domains assessed with the Kiddy-KINDL-R and Kid-/Kiddo-KINDL-R Quality of Life Questionnaire for Children at T0. SDQ = Strengths and Difficulties Questionnaire; n = number of participants; M = mean; SD = standard deviation

Table S14. Results of the regression models for the association of disruptive behavior and emotional problems (SDQ) and quality of life (KINDL) in children from the general population^1^.

|  |  | **Borderline disruptive behavior problems only** | | | |  | **Borderline emotional problems only** | | | |  | **Borderline disruptive behavior and emotional problems both** | | | |  | **Abnormal** | | | |  | **Child's sex** | | | |  | **Child's age** | | | |
| --- | --- | --- | --- | --- | --- | --- | --- | --- | --- | --- | --- | --- | --- | --- | --- | --- | --- | --- | --- | --- | --- | --- | --- | --- | --- | --- | --- | --- | --- | --- |
|  |  | **b** | **95% CI** | | **p** |  | **b** | **95% CI** | | **p** |  | **b** | **95% CI** | | **p** |  | **b** | **95% CI** | | **p** |  | **b** | **95% CI** | | **p** |  | **b** | **95% CI** | | **p** |
| **Physical well-being** |  | -0.60 | -1.09 | -0.12 | .015 |  | -0.85 | -1.51 | -0.18 | .012 |  | -0.75 | -1.74 | 0.25 | .142 |  | -0.91 | -1.21 | -0.61 | < .001 |  | -0.44 | -0.70 | -0.19 | .001 |  | -0.11 | -0.17 | -0.04 | .001 |
| **Emotional well-being** |  | -0.63 | -1.05 | -0.20 | .004 |  | -1.16 | -1.75 | -0.57 | < .001 |  | -2.19 | -3.07 | -1.32 | < .001 |  | -1.83 | -2.10 | -1.57 | < .001 |  | -0.01 | -0.23 | 0.21 | .923 |  | -0.15 | -0.21 | -0.09 | < .001 |
| **Self-esteem** |  | -0.73 | -1.21 | -0.25 | .003 |  | -1.29 | -1.95 | -0.62 | < .001 |  | -1.59 | -2.58 | -0.61 | .002 |  | -1.92 | -2.22 | -1.63 | < .001 |  | 0.42 | 0.17 | 0.67 | .001 |  | -0.19 | -0.25 | -0.12 | < .001 |
| **Family** |  | -1.06 | -1.50 | -0.61 | < .001 |  | -0.75 | -1.37 | -0.13 | .019 |  | -2.02 | -2.94 | -1.10 | < .001 |  | -1.74 | -2.02 | -1.46 | < .001 |  | 0.25 | 0.01 | 0.48 | .041 |  | 0.00 | -0.06 | 0.06 | .969 |
| **Friends** |  | -0.59 | -1.11 | -0.08 | .024 |  | -0.49 | -1.20 | 0.23 | .180 |  | -2.49 | -3.55 | -1.43 | < .001 |  | -1.69 | -2.01 | -1.37 | < .001 |  | 0.39 | 0.12 | 0.66 | .005 |  | -0.10 | -0.17 | -0.03 | .004 |
| **Everyday functioning** |  | -0.63 | -1.15 | -0.11 | .018 |  | -1.27 | -2.00 | -0.54 | .001 |  | -1.80 | -2.87 | -0.73 | .001 |  | -1.53 | -1.86 | -1.21 | < .001 |  | 0.41 | 0.14 | 0.69 | .003 |  | -0.12 | -0.19 | -0.05 | .001 |

Note. The group with normal SDQ results, i.e. absence of disruptive behaviorand emotional problems is used as reference for model calculation. Child’s sex (0 = male, 1 = female) and child’s age are included as covariates. Groups of disruptive behavior and emotional problems are built based on SDQ cut-offs by Goodman (1997). ^1^Subjects who entered the project via other access routes than the screening at the pediatrician are excluded. SDQ = Strengths and Difficulties Questionnaire; KINDL = Kiddy-KINDL-R and Kid-/Kiddo-KINDL-R Quality of Life Questionnaire for Children; b = regression coefficient; CI = confidence interval; p = p-value

Table S15. Results of the regression models for the association of disruptive behavior or emotional problems (SDQ) and quality of life (KINDL) in children from the general population including subjects who entered the project via other access routes than the screening at the pediatrician.

|  |  | **Borderline disruptive behavior problems only** | | | |  | **Borderline emotional problems only** | | | |  | **Borderline disruptive behavior and emotional problems both** | | | |  | **Abnormal** | | | |  | **Child's sex** | | | |  | **Child's age** | | | |
| --- | --- | --- | --- | --- | --- | --- | --- | --- | --- | --- | --- | --- | --- | --- | --- | --- | --- | --- | --- | --- | --- | --- | --- | --- | --- | --- | --- | --- | --- | --- |
|  |  | **b** | **95% CI** | | **p** |  | **b** | **95% CI** | | **p** |  | **b** | **95% CI** | | **p** |  | **b** | **95% CI** | | **p** |  | **b** | **95% CI** | | **p** |  | **b** | **95% CI** | | **p** |
| **Physical well-being** |  | -0.70 | -1.18 | -0.23 | .004 |  | -0.60 | -1.22 | 0.01 | .054 |  | -1.18 | -2.16 | -0.20 | .019 |  | -1.00 | -1.28 | -0.72 | < .001 |  | -0.42 | -0.66 | -0.17 | .001 |  | -0.12 | -0.18 | -0.05 | < .001 |
| **Emotional well-being** |  | -0.68 | -1.10 | -0.25 | .002 |  | -1.25 | -1.79 | -0.71 | < .001 |  | -2.36 | -3.23 | -1.50 | < .001 |  | -1.94 | -2.19 | -1.70 | < .001 |  | -0.04 | -0.26 | 0.18 | .735 |  | -0.15 | -0.20 | -0.09 | < .001 |
| **Self-esteem** |  | -0.82 | -1.28 | -0.35 | .001 |  | -1.15 | -1.75 | -0.54 | < .001 |  | -1.99 | -2.95 | -1.02 | < .001 |  | -1.99 | -2.26 | -1.71 | < .001 |  | 0.36 | 0.12 | 0.60 | .004 |  | -0.19 | -0.26 | -0.13 | < .001 |
| **Family** |  | -1.07 | -1.51 | -0.63 | < .001 |  | -0.53 | -1.10 | 0.03 | .064 |  | -2.03 | -2.92 | -1.13 | < .001 |  | -1.77 | -2.02 | -1.51 | < .001 |  | 0.26 | 0.03 | 0.48 | .027 |  | -0.01 | -0.07 | 0.05 | .792 |
| **Friends** |  | -0.64 | -1.15 | -0.13 | .015 |  | -0.61 | -1.27 | 0.05 | .071 |  | -2.54 | -3.59 | -1.49 | < .001 |  | -1.82 | -2.12 | -1.52 | < .001 |  | 0.39 | 0.12 | 0.65 | .004 |  | -0.10 | -0.17 | -0.03 | .004 |
| **Everyday functioning** |  | -0.66 | -1.17 | -0.15 | .011 |  | -1.18 | -1.84 | -0.51 | .001 |  | -1.88 | -2.92 | -0.83 | < .001 |  | -1.47 | -1.77 | -1.17 | < .001 |  | 0.40 | 0.13 | 0.66 | .003 |  | -0.10 | -0.17 | -0.04 | .003 |

Note. The group with normal SDQ results, i.e. absence of disruptive behavior and emotional problems is used as reference for model calculation. Child’s sex (0 = male, 1 = female) and child’s age are included as covariates. Groups of disruptive behavior and emotional problems are built based on SDQ cut-offs by Goodman (1997). SDQ = Strengths and Difficulties Questionnaire; KINDL = Kiddy-KINDL-R and Kid-/Kiddo-KINDL-R Quality of Life Questionnaire for Children; b = regression coefficient; CI = confidence interval; p = p-value

Table S16. Characteristics of the training groups.

|  |  | **Baghira** | |  | **Tiger** | |  | **NoBaghira** | |  | **NoTiger** | |  | **Group comparison** | |
| --- | --- | --- | --- | --- | --- | --- | --- | --- | --- | --- | --- | --- | --- | --- | --- |
|  |  | n= 192 | |  | n = 145 | |  | n = 350 | |  | n = 245 | |  |  |  |
|  |  | [n = 191] | |  | [n = 143] | |  | [n = 83] | |  | [n = 63] | |  |  |  |
|  |  | **n** | **% / M (SD)** |  | **n** | **% / M (SD)** |  | **n** | **% / M (SD)** |  | **n** | **% / M (SD)** |  | **Chi2 (df)** | **p** |
| **Child’s sex** [%] |  |  |  |  |  |  |  |  |  |  |  |  |  |  |  |
| female |  | 45 | 23.44 |  | 67 | 46.21 |  | 143 | 41.81 |  | 140 | 58.58 |  | 54.44 (3) | < .001 |
|  |  | [45] | [23.56] |  | [65] | [45.45] |  | [35] | [42.17] |  | [37] | [59.68] |  | [33.26 (3)] | [< .001] |
| male |  | 147 | 76.56 |  | 78 | 53.79 |  | 199 | 58.19 |  | 99 | 41.42 |  |  |  |
|  |  | [146] | [76.44] |  | [78] | [54.55] |  | [48] | [57.83] |  | [25] | [40.32] |  |  |  |
| **Child’s age at screening** |  |  |  |  |  |  |  |  |  |  |  |  |  |  |  |
| mean age [M (SD)] |  | 187 | 6.83 (1.88) |  | 143 | 6.56 (1.76) |  | 346 | 6.22 (1.76) |  | 240 | 6.96 (2.03) |  | 23.61 (3) | < .001 |
|  |  | [186] | [6.82 (1.88)] |  | [141] | [6.56 (1.77)] |  | [83] | [6.07 (1.7)] |  | [62] | [6.56 (1.9)] |  | [9.65 (3)] | [.022] |
| by age group [%] |  |  |  |  |  |  |  |  |  |  |  |  |  |  |  |
| 3-6 years |  | 87 | 45.31 |  | 78 | 53.79 |  | 212 | 60.57 |  | 108 | 44.08 |  | 20.08 (3) | < .001 |
|  |  | [87] | [45.55] |  | [77] | [53.85] |  | [55] | [66.27] |  | [32] | [50.79] |  | [10.17 (3)] | [.017] |
| 7-11 years |  | 105 | 54.69 |  | 67 | 46.21 |  | 138 | 39.43 |  | 137 | 55.92 |  |  |  |
|  |  | [104] | [54.45] |  | [66] | [46.15] |  | [28] | [33.73] |  | [31] | [49.21] |  |  |  |
| **SDQ screening result with cut-offs by Goodman (1997) ^1^** [%] |  |  |  |  |  |  |  |  |  |  |  |  |  |  |  |
| normal |  | 9 | 4.81 |  | 15 | 10.71 |  | 25 | 7.14 |  | 24 | 9.84 |  | 334.71 (12) | < .001 |
|  |  | [9] | [4.84] |  | [14] | [10.14] |  | [6] | [7.23] |  | [7] | [11.29] |  | [129.82 (12)] | [< .001] |
| borderline disruptive behavior problems only |  | 23 | 12.3 |  | 5 | 3.57 |  | 145 | 41.43 |  | 5 | 2.05 |  |  |  |
|  |  | [23] | [12.37] |  | [5] | [3.62] |  | [29] | [34.94] |  | [0] | [0] |  |  |  |
| borderline emotional problems only |  | 2 | 1.07 |  | 20 | 14.29 |  | 3 | 0.86 |  | 74 | 30.33 |  |  |  |
|  |  | [2] | [1.08] |  | [20] | [14.49] |  | [2] | [2.41] |  | [22] | [35.48] |  |  |  |
| borderline disruptive behavior & emotional problems |  | 9 | 4.81 |  | 3 | 2.14 |  | 4 | 1.14 |  | 21 | 8.61 |  |  |  |
|  |  | [9] | [4.84] |  | [3] | [2.17] |  | [1] | [1.2] |  | [2] | [3.23] |  |  |  |
| abnormal |  | 144 | 77.01 |  | 97 | 69.29 |  | 173 | 49.43 |  | 120 | 49.18 |  |  |  |
|  |  | [143] | [76.88] |  | [96] | [69.57] |  | [45] | [54.22] |  | [31] | [50] |  |  |  |
| **SDQ screening result with PROMPt project adapted cut-offs^1^** [%] |  |  |  |  |  |  |  |  |  |  |  |  |  |  |  |
| normal |  | 9 | 4.81 |  | 15 | 10.71 |  | 25 | 7.14 |  | 24 | 9.84 |  | 532.98 (12) | < .001 |
|  |  | [9] | [4.84] |  | [14] | [10.14] |  | [6] | [7.23] |  | [7] | [11.29] |  | [229.89 (12)] | [< .001] |
| borderline disruptive behavior problems only |  | 4 | 2.14 |  | 67 | 47.86 |  | 4 | 1.14 |  | 131 | 53.69 |  |  |  |
|  |  | [4] | [2.15] |  | [67] | [48.55] |  | [3] | [3.61] |  | [37] | [59.68] |  |  |  |
| borderline emotional problems only |  | 89 | 47.59 |  | 9 | 6.43 |  | 262 | 74.86 |  | 9 | 3.69 |  |  |  |
|  |  | [88] | [47.31] |  | [9] | [6.52] |  | [57] | [68.67] |  | [0] | [0] |  |  |  |
| borderline disruptive behavior & emotional problems |  | 36 | 19.25 |  | 21 | 15 |  | 27 | 7.71 |  | 50 | 20.49 |  |  |  |
|  |  | [36] | [19.35] |  | [21] | [15.22] |  | [7] | [8.43] |  | [8] | [12.9] |  |  |  |
| abnormal |  | 49 | 26.2 |  | 28 | 20 |  | 32 | 9.14 |  | 30 | 12.3 |  |  |  |
|  |  | [49] | [26.34] |  | [27] | [19.57] |  | [10] | [12.05] |  | [10] | [16.13] |  |  |  |
| **SDQ conduct problems at screening including subjects who entered project via other access routes** [M (SD)] |  | 187 | 4.36 (1.54) |  | 140 | 1.9 (1.61) |  | 350 | 3.65 (1.24) |  | 244 | 1.88 (1.58) |  | 325.63 (3) | < .001 |
|  |  | [186] | [4.36 (1.54)] |  | [138] | [1.91 (1.61)] |  | [83] | [3.75 (1.41)] |  | [62] | [1.69 (1.28)] |  | [190.05 (3)] | [< .001] |
| **SDQ conduct problems at screening without subjects who entered project via other access routes** [M (SD)] |  | 143 | 4.18 (1.46) |  | 88 | 1.89 (1.67) |  | 342 | 3.61 (1.2) |  | 238 | 1.85 (1.5) |  | 268.29 (3) | < .001 |
|  |  | [143] | [4.18 (1.46)] |  | [86] | [1.9 (1.67)] |  | [79] | [3.68 (1.31)] |  | [57] | [1.69 (1.3)] |  | [136.8 (3)] | [< .001] |
| **SDQ emotional problems at screening including subjects who entered project via other access routes** [M (SD)] |  | 187 | 3.05 (2.32) |  | 140 | 5.23 (1.89) |  | 350 | 1.97 (1.76) |  | 244 | 4.66 (1.56) |  | 338.73 (3) | < .001 |
|  |  | [186] | [3.07 (2.31)] |  | [138] | [5.23 (1.88)] |  | [83] | [2.08 (1.93)] |  | [62] | [4.89 (1.67)] |  | [135.96 (3)] | [< .001] |
| **SDQ emotional problems at screening without subjects who entered project via other access routes** [M (SD)] |  | 143 | 2.85 (2.22) |  | 88 | 5.13 (1.72) |  | 342 | 1.93 (1.71) |  | 238 | 4.62 (1.5) |  | 312.77 (3) | < .001 |
|  |  | [143] | [2.85 (2.22)] |  | [86] | [5.12 (1.7)] |  | [79] | [1.94 (1.75)] |  | [57] | [4.75 (1.49)] |  | [120.69 (3)] | [< .001] |
| **KINDL total score at T0^1^** [M (SD)] |  | 186 | 94.46 (9.29) |  | 141 | 93.49 (9.2) |  | 52 | 100.37 (9.2) |  | 43 | 99.33 (8.81) |  | 30.96 (3) | < .001 |
|  |  | [186] | [94.46 (9.29)] |  | [141] | [93.49 (9.2)] |  | [51] | [100.88 (8.52)] |  | [43] | [99.33 (8.81)] |  | [33.22 (3)] | [< .001] |
| **KINDL physical well-being^1^** [M (SD)] |  | 186 | 17.26 (2.37) |  | 142 | 16.7 (2.4) |  | 52 | 17.71 (2.2) |  | 43 | 17.44 (2.31) |  | 10.84 (3) | .013 |
|  |  | [186] | [17.26 (2.37)] |  | [142] | [16.7 (2.4)] |  | [51] | [17.75 (2.21)] |  | [43] | [17.44 (2.31)] |  | [11.36 (3)] | [.010] |
| **KINDL emotional well-being^1^** [M (SD)] |  | 186 | 15.95 (2.2) |  | 141 | 15.16 (2.03) |  | 52 | 16.96 (2.06) |  | 43 | 16.58 (2.06) |  | 33.66 (3) | < .001 |
|  |  | [186] | [15.95 (2.2)] |  | [141] | [15.16 (2.03)] |  | [51] | [17.06 (1.95)] |  | [43] | [16.58 (2.06)] |  | [35.64 (3)] | [< .001] |
| **KINDL self-esteem^1^** [M (SD)] |  | 186 | 14.81 (2.38) |  | 141 | 14.5 (2.12) |  | 52 | 16.21 (2.23) |  | 43 | 15.67 (2.22) |  | 25.15 (3) | < .001 |
|  |  | [186] | [14.81 (2.38)] |  | [141] | [14.5 (2.12)] |  | [51] | [16.31 (2.12)] |  | [43] | [15.67 (2.22)] |  | [27.05 (3)] | [< .001] |
| **KINDL family^1^** [M (SD)] |  | 186 | 14.9 (2.08) |  | 141 | 16.45 (2.24) |  | 52 | 16.54 (1.97) |  | 43 | 16.42 (1.92) |  | 53.53 (3) | < .001 |
|  |  | [186] | [14.9 (2.08)] |  | [141] | [16.45 (2.24)] |  | [51] | [16.55 (1.99)] |  | [43] | [16.42 (1.92)] |  | [53.48 (3)] | [< .001] |
| **KINDL friends^1^** [M (SD)] |  | 186 | 15.35 (2.79) |  | 141 | 15 (2.31) |  | 52 | 16.23 (2.59) |  | 43 | 16.65 (2.33) |  | 23.48 (3) | < .001 |
|  |  | [186] | [15.35 (2.79)] |  | [141] | [15 (2.31)] |  | [51] | [16.41 (2.26)] |  | [43] | [16.65 (2.33)] |  | [24.94 (3)] | [< .001] |
| **KINDL everyday functioning^1^** [M (SD)] |  | 185 | 16.21 (2.42) |  | 140 | 15.7 (2.71) |  | 51 | 16.8 (2.32) |  | 43 | 16.56 (2.29) |  | 7.85 (3) | .049 |
|  |  | [185] | [16.21 (2.42)] |  | [140] | [15.7 (2.71)] |  | [51] | [16.8 (2.32)] |  | [43] | [16.56 (2.29)] |  | [7.85 (3)] | [.049] |

*Note.* Sample characteristics are presented without parentheses for the whole sample, that equals the SDQ analysis sample, and in square brackets for the KINDL analysis sample.

^1^Subjects who entered the project via other access routes than the screening at the pediatrician are included. SDQ = Strengths and Difficulties Questionnaire, KINDL = Kiddy-KINDL-R and Kid-/Kiddo-KINDL-R Quality of Life Questionnaire for Children, Baghira = children who participated in the Baghira training; Tiger = children who participated in the Tiger training; NoBaghira = children who did not participate in the Baghira training despite a recommendation including children with a recommendation for both trainings and a higher or equal SDQ conduct problems than emotional problems score; NoTiger = children who did not participate in the Tiger training despite a recommendation including children with a recommendation for both trainings and a higher SDQ emotional problems than conduct problems score; n = sample size/number of participants; M = mean; SD = standard deviation; df = degrees of freedom; p = p-value

Table S17. Results of the calculated linear mixed effect models on timepoint by training group with multiple imputed data.

|  |  | **Model 1: Screening/T0 and Baghira as reference** | | | | | |  | **Model 2: Screening/T0 and Tiger as reference** | | | | | |
| --- | --- | --- | --- | --- | --- | --- | --- | --- | --- | --- | --- | --- | --- | --- |
|  |  |  | **β** | **SE** | **95% CI** | | **p** |  |  | **β** | **SE** | **95% CI** | | **p** |
| **SDQ Conduct Problems** |  |  |  |  |  |  |  |  |  |  |  |  |  |  |
| Measurement Time Point |  | T1 | -0.54 | 0.09 | -0.72 | -0.35 | < .001 |  | T1 | -0.20 | 0.08 | -0.36 | -0.03 | .018 |
|  |  | T2 | -0.51 | 0.10 | -0.70 | -0.32 | < .001 |  | T2 | -0.07 | 0.10 | -0.26 | 0.12 | .475 |
| Group |  | Tiger | -1.65 | 0.12 | -1.89 | -1.42 | < .001 |  | Baghira | 1.65 | 0.12 | 1.42 | 1.89 | < .001 |
|  |  | NoBaghira | -0.46 | 0.09 | -0.64 | -0.29 | < .001 |  | NoBaghira | 1.19 | 0.10 | 0.99 | 1.39 | < .001 |
|  |  | NoTiger | -1.66 | 0.11 | -1.87 | -1.46 | < .001 |  | NoTiger | -0.01 | 0.12 | -0.24 | 0.22 | .923 |
| Time Point x Group |  | T1 x Tiger | 0.34 | 0.12 | 0.10 | 0.58 | .006 |  | T1 x Baghira | -0.34 | 0.12 | -0.58 | -0.10 | .006 |
|  |  | T1 x NoBaghira | 0.09 | 0.18 | -0.26 | 0.45 | .597 |  | T1 x NoBaghira | -0.25 | 0.17 | -0.59 | 0.10 | .153 |
|  |  | T1 x NoTiger | 1.01 | 0.14 | 0.73 | 1.29 | < .001 |  | T1 x NoTiger | 0.67 | 0.14 | 0.40 | 0.94 | < .001 |
|  |  | T2 x Tiger | 0.44 | 0.13 | 0.18 | 0.71 | .001 |  | T2 x Baghira | -0.44 | 0.13 | -0.71 | -0.18 | .001 |
|  |  | T2 x NoBaghira | -0.03 | 0.18 | -0.40 | 0.34 | .856 |  | T2 x NoBaghira | -0.47 | 0.18 | -0.84 | -0.11 | .013 |
|  |  | T2 x NoTiger | 1.13 | 0.22 | 0.67 | 1.58 | < .001 |  | T2 x NoTiger | 0.69 | 0.22 | 0.24 | 1.13 | .004 |
| **SDQ Emotional Problems** |  |  |  |  |  |  |  |  |  |  |  |  |  |  |
| Measurement Time Point |  | T1 | -0.22 | 0.08 | -0.38 | -0.06 | .007 |  | T1 | -0.90 | 0.09 | -1.09 | -0.72 | < .001 |
|  |  | T2 | -0.19 | 0.09 | -0.37 | -0.01 | .034 |  | T2 | -0.79 | 0.11 | -1.01 | -0.58 | < .001 |
| Group |  | Tiger | 1.16 | 0.12 | 0.92 | 1.41 | < .001 |  | Baghira | -1.16 | 0.12 | -1.41 | -0.92 | < .001 |
|  |  | NoBaghira | -0.57 | 0.10 | -0.77 | -0.37 | < .001 |  | NoBaghira | -1.74 | 0.10 | -1.93 | -1.54 | < .001 |
|  |  | NoTiger | 0.83 | 0.11 | 0.62 | 1.03 | < .001 |  | NoTiger | -0.34 | 0.10 | -0.53 | -0.14 | .001 |
| Time Point x Group |  | T1 x Tiger | -0.68 | 0.13 | -0.93 | -0.43 | < .001 |  | T1 x Baghira | 0.68 | 0.13 | 0.43 | 0.93 | < .001 |
|  |  | T1 x NoBaghira | 0.48 | 0.12 | 0.24 | 0.72 | < .001 |  | T1 x NoBaghira | 1.16 | 0.13 | 0.90 | 1.41 | < .001 |
|  |  | T1 x NoTiger | -0.04 | 0.19 | -0.42 | 0.35 | .846 |  | T1 x NoTiger | 0.64 | 0.20 | 0.24 | 1.04 | .002 |
|  |  | T2 x Tiger | -0.60 | 0.14 | -0.88 | -0.32 | < .001 |  | T2 x Baghira | 0.60 | 0.14 | 0.32 | 0.88 | < .001 |
|  |  | T2 x NoBaghira | 0.47 | 0.14 | 0.18 | 0.76 | .002 |  | T2 x NoBaghira | 1.07 | 0.14 | 0.79 | 1.34 | < .001 |
|  |  | T2 x NoTiger | -0.01 | 0.23 | -0.47 | 0.46 | .982 |  | T2 x NoTiger | 0.60 | 0.23 | 0.12 | 1.07 | .017 |
| **KINDL Physical Well-Being** |  |  |  |  |  |  |  |  |  |  |  |  |  |  |
| Measurement Time Point |  | T1 | 0.00 | 0.07 | -0.14 | 0.14 | .989 |  | T1 | -0.01 | 0.09 | -0.20 | 0.17 | .878 |
|  |  | T2 | -0.08 | 0.09 | -0.26 | 0.10 | .384 |  | T2 | -0.06 | 0.11 | -0.27 | 0.16 | .613 |
| Group |  | Tiger | -0.22 | 0.11 | -0.44 | 0.00 | .047 |  | Baghira | 0.22 | 0.11 | 0.00 | 0.44 | .047 |
|  |  | NoBaghira | 0.09 | 0.13 | -0.16 | 0.34 | .499 |  | NoBaghira | 0.31 | 0.14 | 0.04 | 0.58 | .023 |
|  |  | NoTiger | 0.03 | 0.16 | -0.28 | 0.34 | .851 |  | NoTiger | 0.25 | 0.16 | -0.06 | 0.57 | .113 |
| Time Point x Group |  | T1 x Tiger | -0.02 | 0.12 | -0.25 | 0.22 | .896 |  | T1 x Baghira | 0.02 | 0.12 | -0.22 | 0.25 | .896 |
|  |  | T1 x NoBaghira | 0.02 | 0.17 | -0.33 | 0.37 | .904 |  | T1 x NoBaghira | 0.04 | 0.19 | -0.33 | 0.41 | .845 |
|  |  | T1 x NoTiger | -0.33 | 0.18 | -0.70 | 0.03 | .070 |  | T1 x NoTiger | -0.32 | 0.19 | -0.70 | 0.06 | .099 |
|  |  | T2 x Tiger | 0.02 | 0.14 | -0.25 | 0.30 | .864 |  | T2 x Baghira | -0.02 | 0.14 | -0.30 | 0.25 | .864 |
|  |  | T2 x NoBaghira | -0.02 | 0.18 | -0.36 | 0.33 | .927 |  | T2 x NoBaghira | -0.04 | 0.19 | -0.41 | 0.33 | .829 |
|  |  | T2 x NoTiger | -0.21 | 0.23 | -0.67 | 0.25 | .365 |  | T2 x NoTiger | -0.23 | 0.24 | -0.70 | 0.23 | .321 |
| **KINDL Emotional Well-Being** |  |  |  |  |  |  |  |  |  |  |  |  |  |  |
| Measurement Time Point |  | T1 | 0.23 | 0.08 | 0.08 | 0.39 | .003 |  | T1 | 0.45 | 0.09 | 0.26 | 0.64 | < .001 |
|  |  | T2 | 0.08 | 0.09 | -0.10 | 0.26 | .384 |  | T2 | 0.43 | 0.10 | 0.24 | 0.62 | < .001 |
| Group |  | Tiger | -0.39 | 0.11 | -0.61 | -0.18 | < .001 |  | Baghira | 0.39 | 0.11 | 0.18 | 0.61 | < .001 |
|  |  | NoBaghira | 0.41 | 0.14 | 0.14 | 0.69 | .004 |  | NoBaghira | 0.80 | 0.14 | 0.52 | 1.08 | < .001 |
|  |  | NoTiger | 0.32 | 0.15 | 0.02 | 0.61 | .034 |  | NoTiger | 0.71 | 0.16 | 0.41 | 1.02 | < .001 |
| Time Point x Group |  | T1 x Tiger | 0.22 | 0.12 | -0.03 | 0.46 | .079 |  | T1 x Baghira | -0.22 | 0.12 | -0.46 | 0.03 | .079 |
|  |  | T1 x NoBaghira | -0.17 | 0.18 | -0.52 | 0.19 | .351 |  | T1 x NoBaghira | -0.38 | 0.18 | -0.74 | -0.03 | .033 |
|  |  | T1 x NoTiger | -0.79 | 0.17 | -1.12 | -0.46 | < .001 |  | T1 x NoTiger | -1.01 | 0.17 | -1.35 | -0.66 | < .001 |
|  |  | T2 x Tiger | 0.35 | 0.13 | 0.09 | 0.61 | .009 |  | T2 x Baghira | -0.35 | 0.13 | -0.61 | -0.09 | .009 |
|  |  | T2 x NoBaghira | -0.04 | 0.19 | -0.41 | 0.33 | .839 |  | T2 x NoBaghira | -0.39 | 0.19 | -0.76 | -0.02 | .040 |
|  |  | T2 x NoTiger | -0.69 | 0.22 | -1.13 | -0.24 | .003 |  | T2 x NoTiger | -1.04 | 0.22 | -1.47 | -0.61 | < .001 |
| **KINDL Self-Esteem** |  |  |  |  |  |  |  |  |  |  |  |  |  |  |
| Measurement Time Point |  | T1 | 0.16 | 0.08 | 0.01 | 0.31 | .033 |  | T1 | 0.31 | 0.09 | 0.14 | 0.48 | < .001 |
|  |  | T2 | 0.15 | 0.08 | 0.00 | 0.31 | .055 |  | T2 | 0.24 | 0.09 | 0.05 | 0.42 | .011 |
| Group |  | Tiger | -0.16 | 0.11 | -0.37 | 0.06 | .155 |  | Baghira | 0.16 | 0.11 | -0.06 | 0.37 | .155 |
|  |  | NoBaghira | 0.44 | 0.14 | 0.17 | 0.72 | .002 |  | NoBaghira | 0.60 | 0.14 | 0.32 | 0.88 | < .001 |
|  |  | NoTiger | 0.43 | 0.15 | 0.14 | 0.73 | .004 |  | NoTiger | 0.59 | 0.15 | 0.30 | 0.88 | < .001 |
| Time Point x Group |  | T1 x Tiger | 0.14 | 0.12 | -0.08 | 0.37 | .211 |  | T1 x Baghira | -0.14 | 0.12 | -0.37 | 0.08 | .211 |
|  |  | T1 x NoBaghira | -0.26 | 0.17 | -0.61 | 0.09 | .137 |  | T1 x NoBaghira | -0.41 | 0.18 | -0.76 | -0.05 | .027 |
|  |  | T1 x NoTiger | -0.32 | 0.17 | -0.65 | 0.01 | .057 |  | T1 x NoTiger | -0.47 | 0.17 | -0.81 | -0.12 | .008 |
|  |  | T2 x Tiger | 0.08 | 0.13 | -0.17 | 0.33 | .519 |  | T2 x Baghira | -0.08 | 0.13 | -0.33 | 0.17 | .519 |
|  |  | T2 x NoBaghira | -0.21 | 0.18 | -0.57 | 0.15 | .252 |  | T2 x NoBaghira | -0.29 | 0.20 | -0.68 | 0.10 | .143 |
|  |  | T2 x NoTiger | -0.61 | 0.19 | -0.98 | -0.25 | .001 |  | T2 x NoTiger | -0.70 | 0.19 | -1.06 | -0.33 | < .001 |
| **KINDL Family** |  |  |  |  |  |  |  |  |  |  |  |  |  |  |
| Measurement Time Point |  | T1 | 0.10 | 0.08 | -0.06 | 0.25 | .226 |  | T1 | 0.06 | 0.09 | -0.11 | 0.23 | .475 |
|  |  | T2 | 0.02 | 0.09 | -0.16 | 0.20 | .848 |  | T2 | 0.07 | 0.09 | -0.11 | 0.24 | .447 |
| Group |  | Tiger | 0.74 | 0.11 | 0.52 | 0.97 | < .001 |  | Baghira | -0.74 | 0.11 | -0.97 | -0.52 | < .001 |
|  |  | NoBaghira | 0.69 | 0.14 | 0.41 | 0.98 | < .001 |  | NoBaghira | -0.05 | 0.15 | -0.34 | 0.24 | .739 |
|  |  | NoTiger | 0.80 | 0.15 | 0.51 | 1.10 | < .001 |  | NoTiger | 0.06 | 0.15 | -0.24 | 0.36 | .692 |
| Time Point x Group |  | T1 x Tiger | -0.03 | 0.12 | -0.26 | 0.20 | .776 |  | T1 x Baghira | 0.03 | 0.12 | -0.20 | 0.26 | .776 |
|  |  | T1 x NoBaghira | -0.43 | 0.19 | -0.82 | -0.05 | .027 |  | T1 x NoBaghira | -0.40 | 0.19 | -0.78 | -0.02 | .039 |
|  |  | T1 x NoTiger | -0.32 | 0.18 | -0.68 | 0.03 | .076 |  | T1 x NoTiger | -0.29 | 0.18 | -0.65 | 0.08 | .120 |
|  |  | T2 x Tiger | 0.05 | 0.14 | -0.22 | 0.32 | .713 |  | T2 x Baghira | -0.05 | 0.14 | -0.32 | 0.22 | .713 |
|  |  | T2 x NoBaghira | -0.08 | 0.19 | -0.47 | 0.30 | .664 |  | T2 x NoBaghira | -0.13 | 0.18 | -0.49 | 0.22 | .450 |
|  |  | T2 x NoTiger | -0.47 | 0.20 | -0.86 | -0.08 | .018 |  | T2 x NoTiger | -0.52 | 0.21 | -0.94 | -0.10 | .016 |
| **KINDL Friends** |  |  |  |  |  |  |  |  |  |  |  |  |  |  |
| Measurement Time Point |  | T1 | 0.23 | 0.08 | 0.08 | 0.39 | .003 |  | T1 | 0.46 | 0.09 | 0.28 | 0.63 | < .001 |
|  |  | T2 | 0.04 | 0.10 | -0.15 | 0.23 | .675 |  | T2 | 0.44 | 0.10 | 0.25 | 0.64 | < .001 |
| Group |  | Tiger | -0.15 | 0.11 | -0.37 | 0.06 | .159 |  | Baghira | 0.15 | 0.11 | -0.06 | 0.37 | .159 |
|  |  | NoBaghira | 0.29 | 0.13 | 0.03 | 0.56 | .031 |  | NoBaghira | 0.45 | 0.13 | 0.18 | 0.71 | .001 |
|  |  | NoTiger | 0.49 | 0.15 | 0.19 | 0.78 | .001 |  | NoTiger | 0.64 | 0.15 | 0.35 | 0.93 | < .001 |
| Time Point x Group |  | T1 x Tiger | 0.22 | 0.12 | -0.01 | 0.46 | .058 |  | T1 x Baghira | -0.22 | 0.12 | -0.46 | 0.01 | .058 |
|  |  | T1 x NoBaghira | 0.03 | 0.17 | -0.31 | 0.36 | .879 |  | T1 x NoBaghira | -0.20 | 0.17 | -0.53 | 0.13 | .237 |
|  |  | T1 x NoTiger | -0.52 | 0.18 | -0.88 | -0.16 | .006 |  | T1 x NoTiger | -0.74 | 0.19 | -1.11 | -0.37 | < .001 |
|  |  | T2 x Tiger | 0.40 | 0.14 | 0.13 | 0.67 | .004 |  | T2 x Baghira | -0.40 | 0.14 | -0.67 | -0.13 | .004 |
|  |  | T2 x NoBaghira | 0.00 | 0.19 | -0.37 | 0.37 | .994 |  | T2 x NoBaghira | -0.40 | 0.18 | -0.77 | -0.04 | .032 |
|  |  | T2 x NoTiger | -0.44 | 0.20 | -0.83 | -0.05 | .029 |  | T2 x NoTiger | -0.84 | 0.19 | -1.21 | -0.47 | < .001 |
| **KINDL Everyday Functioning** |  |  |  |  |  |  |  |  |  |  |  |  |  |  |
| Measurement Time Point |  | T1 | 0.15 | 0.08 | -0.02 | 0.31 | .081 |  | T1 | 0.32 | 0.10 | 0.13 | 0.52 | .001 |
|  |  | T2 | 0.00 | 0.10 | -0.20 | 0.20 | .986 |  | T2 | 0.22 | 0.10 | 0.02 | 0.41 | .029 |
| Group |  | Tiger | -0.24 | 0.12 | -0.47 | -0.01 | .045 |  | Baghira | 0.24 | 0.12 | 0.01 | 0.47 | .045 |
|  |  | NoBaghira | 0.07 | 0.14 | -0.21 | 0.35 | .629 |  | NoBaghira | 0.31 | 0.15 | 0.00 | 0.61 | .048 |
|  |  | NoTiger | 0.04 | 0.15 | -0.25 | 0.34 | .768 |  | NoTiger | 0.28 | 0.16 | -0.03 | 0.59 | .079 |
| Time Point x Group |  | T1 x Tiger | 0.18 | 0.13 | -0.08 | 0.43 | .171 |  | T1 x Baghira | -0.18 | 0.13 | -0.43 | 0.08 | .171 |
|  |  | T1 x NoBaghira | -0.09 | 0.18 | -0.45 | 0.26 | .614 |  | T1 x NoBaghira | -0.27 | 0.18 | -0.63 | 0.09 | .145 |
|  |  | T1 x NoTiger | -0.14 | 0.17 | -0.46 | 0.19 | .415 |  | T1 x NoTiger | -0.31 | 0.17 | -0.65 | 0.03 | .070 |
|  |  | T2 x Tiger | 0.22 | 0.14 | -0.06 | 0.49 | .118 |  | T2 x Baghira | -0.22 | 0.14 | -0.49 | 0.06 | .118 |
|  |  | T2 x NoBaghira | 0.02 | 0.21 | -0.40 | 0.44 | .921 |  | T2 x NoBaghira | -0.20 | 0.20 | -0.59 | 0.20 | .322 |
|  |  | T2 x NoTiger | -0.24 | 0.25 | -0.74 | 0.27 | .352 |  | T2 x NoTiger | -0.45 | 0.24 | -0.94 | 0.04 | .068 |

*Note.* SDQ = Strengths and Difficulties Questionnaire, KINDL = Kiddy-KINDL-R and Kid-/Kiddo-KINDL-R Quality of Life Questionnaire for Children, Baghira = children who participated in the Baghira training; Tiger = children who participated in the Tiger training; NoBaghira = children who did not participate in the Baghira training despite a recommendation including children with a recommendation for both trainings and a higher or equal SDQ conduct problems than emotional problems score; NoTiger = children who did not participate in the Tiger training despite a recommendation including children with a recommendation for both trainings and a higher SDQ emotional problems than conduct problems score; β = beta coefficient; SE = standard error; CI = confidene interval; p = p-value

Table S18. Results of the calculated linear mixed effect models on timepoint by assigned group without prior multiple imputation.

|  |  | **Model 1: Screening/T0 and Normal as reference** | | | | | |  | **Model 2: Screening/T0 and Training as reference** | | | | | |
| --- | --- | --- | --- | --- | --- | --- | --- | --- | --- | --- | --- | --- | --- | --- |
|  |  |  | **β** | **SE** | **95% CI** | | **p** |  |  | **β** | **SE** | **95% CI** | | **p** |
| **SDQ Conduct Problems** |  |  |  |  |  |  |  |  |  |  |  |  |  |  |
| Measurement Time Point |  | T1 | 0.26 | 0.03 | 0.20 | 0.33 | < .001 |  | T1 | -0.41 | 0.07 | -0.54 | -0.27 | < .001 |
|  |  | T2 | 0.20 | 0.03 | 0.13 | 0.27 | < .001 |  | T2 | -0.38 | 0.07 | -0.52 | -0.24 | < .001 |
| Group |  | Training | 1.53 | 0.08 | 1.37 | 1.69 | < .001 |  | Normal | -1.53 | 0.08 | -1.69 | -1.37 | < .001 |
|  |  | NoTraining | 1.30 | 0.05 | 1.20 | 1.40 | < .001 |  | NoTraining | -0.23 | 0.09 | -0.41 | -0.05 | .011 |
|  |  | Abnormal | 2.28 | 0.19 | 1.90 | 2.66 | < .001 |  | Abnormal | 0.75 | 0.21 | 0.35 | 1.16 | < .001 |
| Time Point x Group |  | T1 x Training | -0.67 | 0.08 | -0.82 | -0.52 | < .001 |  | T1 x Normal | 0.67 | 0.08 | 0.52 | 0.82 | < .001 |
|  |  | T1 x NoTraining | -0.65 | 0.11 | -0.86 | -0.44 | < .001 |  | T1 x NoTraining | 0.02 | 0.12 | -0.22 | 0.26 | .858 |
|  |  | T1 x Abnormal | -0.61 | 0.19 | -0.99 | -0.24 | .001 |  | T1 x Abnormal | 0.06 | 0.20 | -0.34 | 0.45 | .783 |
|  |  | T2 x Training | -0.58 | 0.08 | -0.73 | -0.42 | < .001 |  | T2 x Normal | 0.58 | 0.08 | 0.42 | 0.73 | < .001 |
|  |  | T2 x NoTraining | -0.62 | 0.12 | -0.85 | -0.38 | < .001 |  | T2 x NoTraining | -0.04 | 0.14 | -0.31 | 0.23 | .768 |
|  |  | T2 x Abnormal | -0.55 | 0.21 | -0.97 | -0.13 | .010 |  | T2 x Abnormal | 0.03 | 0.22 | -0.41 | 0.46 | .899 |
| **SDQ Emotional Problems** |  |  |  |  |  |  |  |  |  |  |  |  |  |  |
| Measurement Time Point |  | T1 | 0.26 | 0.03 | 0.19 | 0.33 | < .001 |  | T1 | -0.58 | 0.07 | -0.72 | -0.44 | < .001 |
|  |  | T2 | 0.24 | 0.04 | 0.17 | 0.32 | < .001 |  | T2 | -0.55 | 0.08 | -0.72 | -0.39 | < .001 |
| Group |  | Training | 1.70 | 0.08 | 1.54 | 1.85 | < .001 |  | Normal | -1.70 | 0.08 | -1.85 | -1.54 | < .001 |
|  |  | NoTraining | 1.13 | 0.05 | 1.02 | 1.24 | < .001 |  | NoTraining | -0.56 | 0.09 | -0.75 | -0.38 | < .001 |
|  |  | Abnormal | 1.61 | 0.15 | 1.31 | 1.90 | < .001 |  | Abnormal | -0.09 | 0.17 | -0.42 | 0.24 | .598 |
| Time Point x Group |  | T1 x Training | -0.84 | 0.08 | -1.00 | -0.68 | < .001 |  | T1 x Normal | 0.84 | 0.08 | 0.68 | 1.00 | < .001 |
|  |  | T1 x NoTraining | -0.53 | 0.12 | -0.77 | -0.29 | < .001 |  | T1 x NoTraining | 0.31 | 0.14 | 0.04 | 0.58 | .025 |
|  |  | T1 x Abnormal | -0.53 | 0.20 | -0.92 | -0.14 | .008 |  | T1 x Abnormal | 0.31 | 0.21 | -0.10 | 0.72 | .138 |
|  |  | T2 x Training | -0.80 | 0.09 | -0.98 | -0.62 | < .001 |  | T2 x Normal | 0.80 | 0.09 | 0.62 | 0.98 | < .001 |
|  |  | T2 x NoTraining | -0.55 | 0.11 | -0.76 | -0.33 | < .001 |  | T2 x NoTraining | 0.25 | 0.13 | -0.01 | 0.51 | .056 |
|  |  | T2 x Abnormal | -0.34 | 0.24 | -0.81 | 0.13 | .157 |  | T2 x Abnormal | 0.46 | 0.25 | -0.03 | 0.95 | .065 |
| **KINDL Physical Well-Being** |  |  |  |  |  |  |  |  |  |  |  |  |  |  |
| Measurement Time Point |  | T1 | -0.19 | 0.04 | -0.27 | -0.10 | < .001 |  | T1 | 0.00 | 0.06 | -0.12 | 0.13 | .944 |
|  |  | T2 | -0.19 | 0.05 | -0.29 | -0.10 | < .001 |  | T2 | -0.01 | 0.08 | -0.16 | 0.14 | .912 |
| Group |  | Training | -0.43 | 0.07 | -0.56 | -0.29 | < .001 |  | Normal | 0.43 | 0.07 | 0.29 | 0.56 | < .001 |
|  |  | NoTraining | -0.19 | 0.11 | -0.41 | 0.02 | .079 |  | NoTraining | 0.23 | 0.12 | 0.00 | 0.47 | .053 |
|  |  | Abnormal | -0.62 | 0.18 | -0.96 | -0.27 | .001 |  | Abnormal | -0.19 | 0.19 | -0.55 | 0.17 | .307 |
| Time Point x Group |  | T1 x Training | 0.19 | 0.08 | 0.04 | 0.34 | .013 |  | T1 x Normal | -0.19 | 0.08 | -0.34 | -0.04 | .013 |
|  |  | T1 x NoTraining | 0.10 | 0.14 | -0.17 | 0.38 | .459 |  | T1 x NoTraining | -0.09 | 0.15 | -0.38 | 0.21 | .560 |
|  |  | T1 x Abnormal | 0.17 | 0.24 | -0.29 | 0.64 | .466 |  | T1 x Abnormal | -0.02 | 0.24 | -0.49 | 0.45 | .938 |
|  |  | T2 x Training | 0.18 | 0.09 | 0.01 | 0.36 | .042 |  | T2 x Normal | -0.18 | 0.09 | -0.36 | -0.01 | .042 |
|  |  | T2 x NoTraining | 0.05 | 0.14 | -0.23 | 0.33 | .735 |  | T2 x NoTraining | -0.14 | 0.15 | -0.44 | 0.17 | .383 |
|  |  | T2 x Abnormal | -0.22 | 0.25 | -0.71 | 0.28 | .390 |  | T2 x Abnormal | -0.40 | 0.26 | -0.91 | 0.11 | .123 |
| **KINDL Emotional Well-Being** |  |  |  |  |  |  |  |  |  |  |  |  |  |  |
| Measurement Time Point |  | T1 | -0.07 | 0.04 | -0.15 | 0.00 | .062 |  | T1 | 0.38 | 0.07 | 0.24 | 0.52 | < .001 |
|  |  | T2 | -0.07 | 0.04 | -0.15 | 0.00 | .067 |  | T2 | 0.33 | 0.08 | 0.17 | 0.48 | < .001 |
| Group |  | Training | -1.04 | 0.07 | -1.18 | -0.91 | < .001 |  | Normal | 1.04 | 0.07 | 0.91 | 1.18 | < .001 |
|  |  | NoTraining | -0.37 | 0.10 | -0.57 | -0.17 | < .001 |  | NoTraining | 0.67 | 0.12 | 0.44 | 0.90 | < .001 |
|  |  | Abnormal | -1.13 | 0.20 | -1.53 | -0.74 | < .001 |  | Abnormal | -0.09 | 0.21 | -0.50 | 0.32 | .670 |
| Time Point x Group |  | T1 x Training | 0.45 | 0.08 | 0.29 | 0.61 | < .001 |  | T1 x Normal | -0.45 | 0.08 | -0.61 | -0.29 | < .001 |
|  |  | T1 x NoTraining | -0.01 | 0.13 | -0.27 | 0.25 | .932 |  | T1 x NoTraining | -0.46 | 0.15 | -0.75 | -0.17 | .002 |
|  |  | T1 x Abnormal | -0.11 | 0.24 | -0.59 | 0.37 | .649 |  | T1 x Abnormal | -0.56 | 0.25 | -1.05 | -0.07 | .026 |
|  |  | T2 x Training | 0.40 | 0.09 | 0.23 | 0.58 | < .001 |  | T2 x Normal | -0.40 | 0.09 | -0.58 | -0.23 | < .001 |
|  |  | T2 x NoTraining | -0.07 | 0.15 | -0.36 | 0.21 | .609 |  | T2 x NoTraining | -0.48 | 0.16 | -0.79 | -0.16 | .003 |
|  |  | T2 x Abnormal | -0.32 | 0.21 | -0.72 | 0.09 | .123 |  | T2 x Abnormal | -0.72 | 0.22 | -1.15 | -0.29 | .001 |
| **KINDL Self-Esteem** |  |  |  |  |  |  |  |  |  |  |  |  |  |  |
| Measurement Time Point |  | T1 | -0.09 | 0.04 | -0.16 | -0.02 | .009 |  | T1 | 0.26 | 0.06 | 0.13 | 0.38 | < .001 |
|  |  | T2 | -0.10 | 0.04 | -0.17 | -0.03 | .006 |  | T2 | 0.25 | 0.07 | 0.13 | 0.38 | < .001 |
| Group |  | Training | -0.92 | 0.07 | -1.05 | -0.79 | < .001 |  | Normal | 0.92 | 0.07 | 0.79 | 1.05 | < .001 |
|  |  | NoTraining | -0.32 | 0.10 | -0.53 | -0.12 | .002 |  | NoTraining | 0.60 | 0.12 | 0.37 | 0.83 | < .001 |
|  |  | Abnormal | -1.08 | 0.20 | -1.46 | -0.69 | < .001 |  | Abnormal | -0.15 | 0.20 | -0.55 | 0.24 | .446 |
| Time Point x Group |  | T1 x Training | 0.35 | 0.07 | 0.21 | 0.49 | < .001 |  | T1 x Normal | -0.35 | 0.07 | -0.49 | -0.21 | < .001 |
|  |  | T1 x NoTraining | 0.01 | 0.12 | -0.22 | 0.25 | .911 |  | T1 x NoTraining | -0.33 | 0.13 | -0.59 | -0.08 | .011 |
|  |  | T1 x Abnormal | -0.16 | 0.20 | -0.56 | 0.23 | .424 |  | T1 x Abnormal | -0.51 | 0.21 | -0.92 | -0.10 | .014 |
|  |  | T2 x Training | 0.36 | 0.08 | 0.21 | 0.50 | < .001 |  | T2 x Normal | -0.36 | 0.08 | -0.50 | -0.21 | < .001 |
|  |  | T2 x NoTraining | -0.06 | 0.14 | -0.32 | 0.21 | .672 |  | T2 x NoTraining | -0.41 | 0.15 | -0.70 | -0.13 | .005 |
|  |  | T2 x Abnormal | 0.11 | 0.21 | -0.30 | 0.53 | .589 |  | T2 x Abnormal | -0.24 | 0.22 | -0.67 | 0.19 | .272 |
| **KINDL Family** |  |  |  |  |  |  |  |  |  |  |  |  |  |  |
| Measurement Time Point |  | T1 | -0.12 | 0.03 | -0.18 | -0.06 | < .001 |  | T1 | 0.11 | 0.07 | -0.02 | 0.24 | .085 |
|  |  | T2 | -0.07 | 0.04 | -0.14 | 0.00 | .056 |  | T2 | 0.10 | 0.07 | -0.04 | 0.23 | .160 |
| Group |  | Training | -0.87 | 0.07 | -1.01 | -0.72 | < .001 |  | Normal | 0.87 | 0.07 | 0.72 | 1.01 | < .001 |
|  |  | NoTraining | -0.36 | 0.10 | -0.57 | -0.16 | < .001 |  | NoTraining | 0.50 | 0.12 | 0.27 | 0.73 | < .001 |
|  |  | Abnormal | -1.04 | 0.17 | -1.38 | -0.70 | < .001 |  | Abnormal | -0.18 | 0.18 | -0.54 | 0.18 | .337 |
| Time Point x Group |  | T1 x Training | 0.23 | 0.07 | 0.09 | 0.38 | .001 |  | T1 x Normal | -0.23 | 0.07 | -0.38 | -0.09 | .001 |
|  |  | T1 x NoTraining | -0.13 | 0.11 | -0.35 | 0.09 | .263 |  | T1 x NoTraining | -0.36 | 0.13 | -0.60 | -0.11 | .004 |
|  |  | T1 x Abnormal | -0.05 | 0.22 | -0.47 | 0.37 | .816 |  | T1 x Abnormal | -0.28 | 0.22 | -0.72 | 0.15 | .205 |
|  |  | T2 x Training | 0.16 | 0.08 | 0.01 | 0.31 | .033 |  | T2 x Normal | -0.16 | 0.08 | -0.31 | -0.01 | .033 |
|  |  | T2 x NoTraining | -0.12 | 0.15 | -0.42 | 0.17 | .415 |  | T2 x NoTraining | -0.29 | 0.16 | -0.60 | 0.03 | .078 |
|  |  | T2 x Abnormal | -0.18 | 0.21 | -0.59 | 0.24 | .401 |  | T2 x Abnormal | -0.34 | 0.22 | -0.77 | 0.09 | .121 |
| **KINDL Friends** |  |  |  |  |  |  |  |  |  |  |  |  |  |  |
| Measurement Time Point |  | T1 | 0.06 | 0.04 | -0.02 | 0.14 | .153 |  | T1 | 0.40 | 0.06 | 0.27 | 0.53 | < .001 |
|  |  | T2 | 0.11 | 0.04 | 0.04 | 0.19 | .002 |  | T2 | 0.32 | 0.08 | 0.16 | 0.48 | < .001 |
| Group |  | Training | -0.79 | 0.07 | -0.93 | -0.65 | < .001 |  | Normal | 0.79 | 0.07 | 0.65 | 0.93 | < .001 |
|  |  | NoTraining | -0.22 | 0.10 | -0.42 | -0.02 | .032 |  | NoTraining | 0.57 | 0.12 | 0.34 | 0.80 | < .001 |
|  |  | Abnormal | -1.05 | 0.19 | -1.43 | -0.67 | < .001 |  | Abnormal | -0.26 | 0.20 | -0.65 | 0.13 | .191 |
| Time Point x Group |  | T1 x Training | 0.34 | 0.08 | 0.19 | 0.49 | < .001 |  | T1 x Normal | -0.34 | 0.08 | -0.49 | -0.19 | < .001 |
|  |  | T1 x NoTraining | 0.05 | 0.13 | -0.21 | 0.30 | .714 |  | T1 x NoTraining | -0.29 | 0.14 | -0.57 | -0.02 | .035 |
|  |  | T1 x Abnormal | -0.16 | 0.23 | -0.61 | 0.29 | .482 |  | T1 x Abnormal | -0.50 | 0.23 | -0.96 | -0.05 | .031 |
|  |  | T2 x Training | 0.21 | 0.09 | 0.03 | 0.38 | .020 |  | T2 x Normal | -0.21 | 0.09 | -0.38 | -0.03 | .020 |
|  |  | T2 x NoTraining | -0.19 | 0.15 | -0.48 | 0.09 | .188 |  | T2 x NoTraining | -0.40 | 0.16 | -0.72 | -0.08 | .014 |
|  |  | T2 x Abnormal | 0.02 | 0.21 | -0.40 | 0.44 | .932 |  | T2 x Abnormal | -0.19 | 0.23 | -0.63 | 0.26 | .404 |
| **KINDL Everyday Functioning** |  |  |  |  |  |  |  |  |  |  |  |  |  |  |
| Measurement Time Point |  | T1 | -0.07 | 0.04 | -0.15 | 0.00 | .047 |  | T1 | 0.27 | 0.07 | 0.13 | 0.41 | < .001 |
|  |  | T2 | -0.09 | 0.04 | -0.16 | -0.01 | .025 |  | T2 | 0.16 | 0.08 | 0.01 | 0.31 | .038 |
| Group |  | Training | -0.60 | 0.07 | -0.74 | -0.46 | < .001 |  | Normal | 0.60 | 0.07 | 0.46 | 0.74 | < .001 |
|  |  | NoTraining | -0.37 | 0.11 | -0.57 | -0.16 | .001 |  | NoTraining | 0.24 | 0.12 | 0.01 | 0.47 | .044 |
|  |  | Abnormal | -0.89 | 0.18 | -1.23 | -0.54 | < .001 |  | Abnormal | -0.28 | 0.18 | -0.64 | 0.07 | .118 |
| Time Point x Group |  | T1 x Training | 0.35 | 0.08 | 0.19 | 0.51 | < .001 |  | T1 x Normal | -0.35 | 0.08 | -0.51 | -0.19 | < .001 |
|  |  | T1 x NoTraining | 0.17 | 0.13 | -0.07 | 0.42 | .168 |  | T1 x NoTraining | -0.18 | 0.14 | -0.45 | 0.10 | .205 |
|  |  | T1 x Abnormal | 0.03 | 0.19 | -0.35 | 0.40 | .885 |  | T1 x Abnormal | -0.32 | 0.20 | -0.71 | 0.07 | .108 |
|  |  | T2 x Training | 0.25 | 0.09 | 0.08 | 0.42 | .004 |  | T2 x Normal | -0.25 | 0.09 | -0.42 | -0.08 | .004 |
|  |  | T2 x NoTraining | 0.02 | 0.15 | -0.28 | 0.31 | .920 |  | T2 x NoTraining | -0.23 | 0.16 | -0.55 | 0.09 | .157 |
|  |  | T2 x Abnormal | 0.11 | 0.23 | -0.33 | 0.55 | .628 |  | T2 x Abnormal | -0.14 | 0.24 | -0.60 | 0.32 | .560 |

Note. SDQ = Strengths and Difficulties Questionnaire, KINDL = Kiddy-KINDL-R and Kid-/Kiddo-KINDL-R Quality of Life Questionnaire for Children, Normal = children evaluated as normal with no recommendation for indicated prevention participation; Training = children who participated in an indicated prevention program after recommendation, NoTraining = children who did not participate in an indicated prevention program despite recommendation; Abnormal = children with abnormal or clinically significant disruptive behavior or emotional problems or that did not fullfill inclusion criteria for participation in an indicated prevention program; β = beta coefficient; SE = standard error; CI = confidene interval; p = p-value

Table S19. Results of the calculated linear mixed effect models on timepoint by training group without prior multiple impution.

|  |  | **Model 1: Screening/T0 and Baghira as reference** | | | | | |  | **Model 2: Screening/T0 and Tiger as reference** | | | | | |
| --- | --- | --- | --- | --- | --- | --- | --- | --- | --- | --- | --- | --- | --- | --- |
|  |  |  | **β** | **SE** | **95% CI** | | **p** |  |  | **β** | **SE** | **95% CI** | | **p** |
| **SDQ Conduct Problems** |  |  |  |  |  |  |  |  |  |  |  |  |  |  |
| Measurement Time Point |  | T1 | -0.53 | 0.10 | -0.72 | -0.34 | < .001 |  | T1 | -0.19 | 0.08 | -0.36 | -0.03 | .020 |
|  |  | T2 | -0.55 | 0.10 | -0.73 | -0.36 | < .001 |  | T2 | -0.12 | 0.10 | -0.31 | 0.07 | .222 |
| Group |  | Tiger | -1.64 | 0.12 | -1.88 | -1.41 | < .001 |  | Baghira | 1.64 | 0.12 | 1.41 | 1.88 | < .001 |
|  |  | NoBaghira | -0.45 | 0.09 | -0.63 | -0.27 | < .001 |  | NoBaghira | 1.20 | 0.10 | 0.99 | 1.40 | < .001 |
|  |  | NoTiger | -1.65 | 0.11 | -1.86 | -1.44 | < .001 |  | NoTiger | -0.01 | 0.12 | -0.24 | 0.22 | .943 |
| Time Point x Group |  | T1 x Tiger | 0.33 | 0.13 | 0.08 | 0.58 | .009 |  | T1 x Baghira | -0.33 | 0.13 | -0.58 | -0.08 | .009 |
|  |  | T1 x NoBaghira | -0.18 | 0.16 | -0.49 | 0.14 | .272 |  | T1 x NoBaghira | -0.51 | 0.15 | -0.81 | -0.21 | .001 |
|  |  | T1 x NoTiger | 0.64 | 0.17 | 0.30 | 0.97 | < .001 |  | T1 x NoTiger | 0.30 | 0.17 | -0.02 | 0.63 | .067 |
|  |  | T2 x Tiger | 0.43 | 0.14 | 0.16 | 0.70 | .002 |  | T2 x Baghira | -0.43 | 0.14 | -0.70 | -0.16 | .002 |
|  |  | T2 x NoBaghira | -0.26 | 0.17 | -0.59 | 0.08 | .137 |  | T2 x NoBaghira | -0.68 | 0.17 | -1.02 | -0.35 | < .001 |
|  |  | T2 x NoTiger | 0.79 | 0.17 | 0.45 | 1.13 | < .001 |  | T2 x NoTiger | 0.36 | 0.17 | 0.02 | 0.70 | .036 |
| **SDQ Emotional Problems** |  |  |  |  |  |  |  |  |  |  |  |  |  |  |
| Measurement Time Point |  | T1 | -0.24 | 0.09 | -0.41 | -0.07 | .005 |  | T1 | -0.91 | 0.10 | -1.10 | -0.72 | < .001 |
|  |  | T2 | -0.25 | 0.09 | -0.43 | -0.07 | .008 |  | T2 | -0.85 | 0.12 | -1.08 | -0.61 | < .001 |
| Group |  | Tiger | 1.18 | 0.13 | 0.93 | 1.42 | < .001 |  | Baghira | -1.18 | 0.13 | -1.42 | -0.93 | < .001 |
|  |  | NoBaghira | -0.56 | 0.10 | -0.76 | -0.35 | < .001 |  | NoBaghira | -1.74 | 0.10 | -1.93 | -1.54 | < .001 |
|  |  | NoTiger | 0.83 | 0.11 | 0.61 | 1.04 | < .001 |  | NoTiger | -0.35 | 0.10 | -0.55 | -0.15 | .001 |
| Time Point x Group |  | T1 x Tiger | -0.67 | 0.13 | -0.92 | -0.42 | < .001 |  | T1 x Baghira | 0.67 | 0.13 | 0.42 | 0.92 | < .001 |
|  |  | T1 x NoBaghira | 0.22 | 0.15 | -0.08 | 0.52 | .151 |  | T1 x NoBaghira | 0.89 | 0.16 | 0.58 | 1.20 | < .001 |
|  |  | T1 x NoTiger | -0.33 | 0.19 | -0.71 | 0.05 | .091 |  | T1 x NoTiger | 0.34 | 0.20 | -0.05 | 0.73 | .087 |
|  |  | T2 x Tiger | -0.60 | 0.15 | -0.89 | -0.30 | < .001 |  | T2 x Baghira | 0.60 | 0.15 | 0.30 | 0.89 | < .001 |
|  |  | T2 x NoBaghira | 0.24 | 0.15 | -0.05 | 0.52 | .107 |  | T2 x NoBaghira | 0.83 | 0.16 | 0.51 | 1.15 | < .001 |
|  |  | T2 x NoTiger | -0.37 | 0.18 | -0.73 | -0.01 | .045 |  | T2 x NoTiger | 0.23 | 0.20 | -0.17 | 0.62 | .258 |
| **KINDL Physical Well-Being** |  |  |  |  |  |  |  |  |  |  |  |  |  |  |
| Measurement Time Point |  | T1 | 0.00 | 0.07 | -0.14 | 0.14 | .996 |  | T1 | 0.01 | 0.10 | -0.18 | 0.20 | .916 |
|  |  | T2 | 0.00 | 0.09 | -0.18 | 0.19 | .972 |  | T2 | -0.02 | 0.10 | -0.22 | 0.19 | .868 |
| Group |  | Tiger | -0.24 | 0.11 | -0.46 | -0.01 | .039 |  | Baghira | 0.24 | 0.11 | 0.01 | 0.46 | .039 |
|  |  | NoBaghira | 0.15 | 0.14 | -0.13 | 0.43 | .300 |  | NoBaghira | 0.38 | 0.15 | 0.09 | 0.68 | .011 |
|  |  | NoTiger | 0.03 | 0.16 | -0.29 | 0.36 | .837 |  | NoTiger | 0.27 | 0.17 | -0.06 | 0.60 | .113 |
| Time Point x Group |  | T1 x Tiger | 0.01 | 0.12 | -0.23 | 0.25 | .931 |  | T1 x Baghira | -0.01 | 0.12 | -0.25 | 0.23 | .931 |
|  |  | T1 x NoBaghira | 0.11 | 0.17 | -0.23 | 0.44 | .531 |  | T1 x NoBaghira | 0.10 | 0.18 | -0.26 | 0.45 | .597 |
|  |  | T1 x NoTiger | -0.31 | 0.20 | -0.71 | 0.09 | .124 |  | T1 x NoTiger | -0.32 | 0.21 | -0.74 | 0.09 | .129 |
|  |  | T2 x Tiger | -0.02 | 0.14 | -0.30 | 0.26 | .883 |  | T2 x Baghira | 0.02 | 0.14 | -0.26 | 0.30 | .883 |
|  |  | T2 x NoBaghira | -0.08 | 0.17 | -0.42 | 0.25 | .623 |  | T2 x NoBaghira | -0.06 | 0.18 | -0.41 | 0.28 | .721 |
|  |  | T2 x NoTiger | -0.21 | 0.24 | -0.68 | 0.26 | .378 |  | T2 x NoTiger | -0.19 | 0.24 | -0.66 | 0.28 | .434 |
| **KINDL Emotional Well-Being** |  |  |  |  |  |  |  |  |  |  |  |  |  |  |
| Measurement Time Point |  | T1 | 0.25 | 0.08 | 0.08 | 0.41 | .003 |  | T1 | 0.46 | 0.10 | 0.27 | 0.65 | < .001 |
|  |  | T2 | 0.15 | 0.10 | -0.05 | 0.34 | .146 |  | T2 | 0.49 | 0.10 | 0.30 | 0.69 | < .001 |
| Group |  | Tiger | -0.40 | 0.11 | -0.62 | -0.18 | < .001 |  | Baghira | 0.40 | 0.11 | 0.18 | 0.62 | < .001 |
|  |  | NoBaghira | 0.46 | 0.14 | 0.19 | 0.73 | .001 |  | NoBaghira | 0.86 | 0.14 | 0.58 | 1.13 | < .001 |
|  |  | NoTiger | 0.34 | 0.16 | 0.03 | 0.65 | .032 |  | NoTiger | 0.74 | 0.16 | 0.42 | 1.05 | < .001 |
| Time Point x Group |  | T1 x Tiger | 0.21 | 0.13 | -0.04 | 0.47 | .093 |  | T1 x Baghira | -0.21 | 0.13 | -0.47 | 0.04 | .093 |
|  |  | T1 x NoBaghira | -0.06 | 0.15 | -0.35 | 0.24 | .711 |  | T1 x NoBaghira | -0.27 | 0.16 | -0.58 | 0.04 | .089 |
|  |  | T1 x NoTiger | -0.63 | 0.21 | -1.03 | -0.23 | .002 |  | T1 x NoTiger | -0.85 | 0.21 | -1.26 | -0.43 | < .001 |
|  |  | T2 x Tiger | 0.35 | 0.14 | 0.07 | 0.62 | .013 |  | T2 x Baghira | -0.35 | 0.14 | -0.62 | -0.07 | .013 |
|  |  | T2 x NoBaghira | -0.07 | 0.18 | -0.43 | 0.28 | .680 |  | T2 x NoBaghira | -0.42 | 0.18 | -0.78 | -0.07 | .019 |
|  |  | T2 x NoTiger | -0.58 | 0.22 | -1.02 | -0.15 | .009 |  | T2 x NoTiger | -0.93 | 0.22 | -1.37 | -0.50 | < .001 |
| **KINDL Self-Esteem** |  |  |  |  |  |  |  |  |  |  |  |  |  |  |
| Measurement Time Point |  | T1 | 0.16 | 0.08 | 0.01 | 0.32 | .040 |  | T1 | 0.34 | 0.09 | 0.17 | 0.51 | < .001 |
|  |  | T2 | 0.20 | 0.08 | 0.04 | 0.36 | .013 |  | T2 | 0.30 | 0.10 | 0.11 | 0.48 | .002 |
| Group |  | Tiger | -0.17 | 0.11 | -0.39 | 0.04 | .116 |  | Baghira | 0.17 | 0.11 | -0.04 | 0.39 | .116 |
|  |  | NoBaghira | 0.50 | 0.14 | 0.23 | 0.77 | < .001 |  | NoBaghira | 0.67 | 0.14 | 0.40 | 0.95 | < .001 |
|  |  | NoTiger | 0.45 | 0.17 | 0.12 | 0.78 | .007 |  | NoTiger | 0.63 | 0.17 | 0.30 | 0.95 | < .001 |
| Time Point x Group |  | T1 x Tiger | 0.18 | 0.12 | -0.05 | 0.41 | .125 |  | T1 x Baghira | -0.18 | 0.12 | -0.41 | 0.05 | .125 |
|  |  | T1 x NoBaghira | -0.19 | 0.15 | -0.50 | 0.11 | .209 |  | T1 x NoBaghira | -0.38 | 0.16 | -0.69 | -0.06 | .019 |
|  |  | T1 x NoTiger | -0.26 | 0.20 | -0.64 | 0.12 | .185 |  | T1 x NoTiger | -0.44 | 0.20 | -0.83 | -0.05 | .027 |
|  |  | T2 x Tiger | 0.10 | 0.12 | -0.15 | 0.34 | .445 |  | T2 x Baghira | -0.10 | 0.12 | -0.34 | 0.15 | .445 |
|  |  | T2 x NoBaghira | -0.22 | 0.16 | -0.54 | 0.10 | .186 |  | T2 x NoBaghira | -0.31 | 0.17 | -0.65 | 0.02 | .069 |
|  |  | T2 x NoTiger | -0.55 | 0.22 | -0.99 | -0.11 | .015 |  | T2 x NoTiger | -0.64 | 0.23 | -1.09 | -0.19 | .005 |
| **KINDL Family** |  |  |  |  |  |  |  |  |  |  |  |  |  |  |
| Measurement Time Point |  | T1 | 0.12 | 0.08 | -0.05 | 0.28 | .163 |  | T1 | 0.09 | 0.09 | -0.09 | 0.26 | .331 |
|  |  | T2 | 0.06 | 0.09 | -0.13 | 0.24 | .547 |  | T2 | 0.13 | 0.08 | -0.02 | 0.29 | .094 |
| Group |  | Tiger | 0.73 | 0.12 | 0.50 | 0.96 | < .001 |  | Baghira | -0.73 | 0.12 | -0.96 | -0.50 | < .001 |
|  |  | NoBaghira | 0.78 | 0.15 | 0.50 | 1.07 | < .001 |  | NoBaghira | 0.05 | 0.15 | -0.25 | 0.35 | .739 |
|  |  | NoTiger | 0.81 | 0.16 | 0.49 | 1.12 | < .001 |  | NoTiger | 0.08 | 0.17 | -0.25 | 0.40 | .649 |
| Time Point x Group |  | T1 x Tiger | -0.03 | 0.12 | -0.27 | 0.21 | .809 |  | T1 x Baghira | 0.03 | 0.12 | -0.21 | 0.27 | .809 |
|  |  | T1 x NoBaghira | -0.42 | 0.15 | -0.72 | -0.12 | .007 |  | T1 x NoBaghira | -0.39 | 0.16 | -0.70 | -0.08 | .013 |
|  |  | T1 x NoTiger | -0.23 | 0.18 | -0.58 | 0.13 | .216 |  | T1 x NoTiger | -0.20 | 0.19 | -0.56 | 0.17 | .289 |
|  |  | T2 x Tiger | 0.08 | 0.12 | -0.16 | 0.32 | .530 |  | T2 x Baghira | -0.08 | 0.12 | -0.32 | 0.16 | .530 |
|  |  | T2 x NoBaghira | -0.15 | 0.20 | -0.53 | 0.24 | .462 |  | T2 x NoBaghira | -0.22 | 0.19 | -0.60 | 0.15 | .246 |
|  |  | T2 x NoTiger | -0.36 | 0.24 | -0.83 | 0.11 | .131 |  | T2 x NoTiger | -0.44 | 0.23 | -0.89 | 0.02 | .061 |
| **KINDL Friends** |  |  |  |  |  |  |  |  |  |  |  |  |  |  |
| Measurement Time Point |  | T1 | 0.27 | 0.08 | 0.12 | 0.42 | .001 |  | T1 | 0.48 | 0.09 | 0.31 | 0.65 | < .001 |
|  |  | T2 | 0.12 | 0.10 | -0.07 | 0.31 | .223 |  | T2 | 0.51 | 0.10 | 0.30 | 0.71 | < .001 |
| Group |  | Tiger | -0.15 | 0.11 | -0.37 | 0.06 | .164 |  | Baghira | 0.15 | 0.11 | -0.06 | 0.37 | .164 |
|  |  | NoBaghira | 0.35 | 0.13 | 0.09 | 0.62 | .009 |  | NoBaghira | 0.50 | 0.14 | 0.24 | 0.77 | < .001 |
|  |  | NoTiger | 0.55 | 0.16 | 0.25 | 0.86 | < .001 |  | NoTiger | 0.71 | 0.15 | 0.40 | 1.01 | < .001 |
| Time Point x Group |  | T1 x Tiger | 0.21 | 0.12 | -0.02 | 0.44 | .068 |  | T1 x Baghira | -0.21 | 0.12 | -0.44 | 0.02 | .068 |
|  |  | T1 x NoBaghira | 0.10 | 0.15 | -0.19 | 0.40 | .503 |  | T1 x NoBaghira | -0.11 | 0.16 | -0.42 | 0.20 | .473 |
|  |  | T1 x NoTiger | -0.50 | 0.18 | -0.86 | -0.15 | .005 |  | T1 x NoTiger | -0.72 | 0.19 | -1.08 | -0.35 | < .001 |
|  |  | T2 x Tiger | 0.39 | 0.14 | 0.11 | 0.67 | .007 |  | T2 x Baghira | -0.39 | 0.14 | -0.67 | -0.11 | .007 |
|  |  | T2 x NoBaghira | -0.05 | 0.18 | -0.41 | 0.30 | .777 |  | T2 x NoBaghira | -0.44 | 0.19 | -0.80 | -0.08 | .018 |
|  |  | T2 x NoTiger | -0.37 | 0.24 | -0.83 | 0.09 | .119 |  | T2 x NoTiger | -0.75 | 0.24 | -1.22 | -0.29 | .002 |
| **KINDL Everyday Functioning** |  |  |  |  |  |  |  |  |  |  |  |  |  |  |
| Measurement Time Point |  | T1 | 0.18 | 0.08 | 0.02 | 0.35 | .032 |  | T1 | 0.35 | 0.10 | 0.14 | 0.55 | .001 |
|  |  | T2 | 0.06 | 0.10 | -0.14 | 0.25 | .564 |  | T2 | 0.27 | 0.10 | 0.08 | 0.46 | .006 |
| Group |  | Tiger | -0.27 | 0.12 | -0.50 | -0.03 | .024 |  | Baghira | 0.27 | 0.12 | 0.03 | 0.50 | .024 |
|  |  | NoBaghira | 0.13 | 0.14 | -0.14 | 0.41 | .348 |  | NoBaghira | 0.40 | 0.15 | 0.10 | 0.69 | .009 |
|  |  | NoTiger | 0.07 | 0.16 | -0.25 | 0.38 | .680 |  | NoTiger | 0.33 | 0.17 | 0.00 | 0.67 | .050 |
| Time Point x Group |  | T1 x Tiger | 0.16 | 0.13 | -0.10 | 0.42 | .219 |  | T1 x Baghira | -0.16 | 0.13 | -0.42 | 0.10 | .219 |
|  |  | T1 x NoBaghira | -0.08 | 0.17 | -0.40 | 0.24 | .632 |  | T1 x NoBaghira | -0.24 | 0.17 | -0.58 | 0.10 | .165 |
|  |  | T1 x NoTiger | -0.09 | 0.19 | -0.47 | 0.29 | .638 |  | T1 x NoTiger | -0.26 | 0.20 | -0.65 | 0.14 | .208 |
|  |  | T2 x Tiger | 0.21 | 0.14 | -0.06 | 0.48 | .132 |  | T2 x Baghira | -0.21 | 0.14 | -0.48 | 0.06 | .132 |
|  |  | T2 x NoBaghira | -0.04 | 0.19 | -0.42 | 0.34 | .839 |  | T2 x NoBaghira | -0.25 | 0.19 | -0.63 | 0.13 | .193 |
|  |  | T2 x NoTiger | -0.27 | 0.23 | -0.73 | 0.18 | .241 |  | T2 x NoTiger | -0.48 | 0.23 | -0.94 | -0.03 | .037 |

*Note.* SDQ = Strengths and Difficulties Questionnaire, KINDL = Kiddy-KINDL-R and Kid-/Kiddo-KINDL-R Quality of Life Questionnaire for Children, Baghira = children who participated in the Baghira training; Tiger = children who participated in the Tiger training; NoBaghira = children who did not participate in the Baghira training despite a recommendation including children with a recommendation for both trainings and a higher or equal SDQ conduct problems than emotional problems score; NoTiger = children who did not participate in the Tiger training despite a recommendation including children with a recommendation for both trainings and a higher SDQ emotional problems than conduct problems score; β = beta coefficient; SE = standard error; CI = confidene interval; p = p-value
